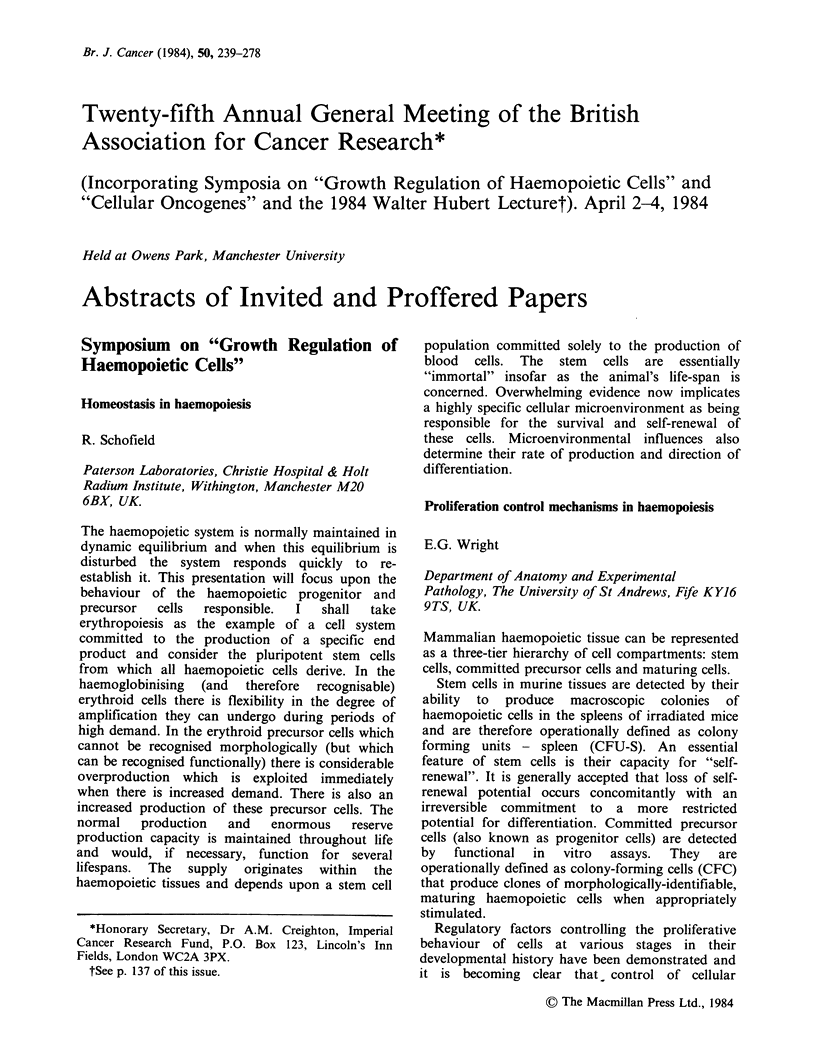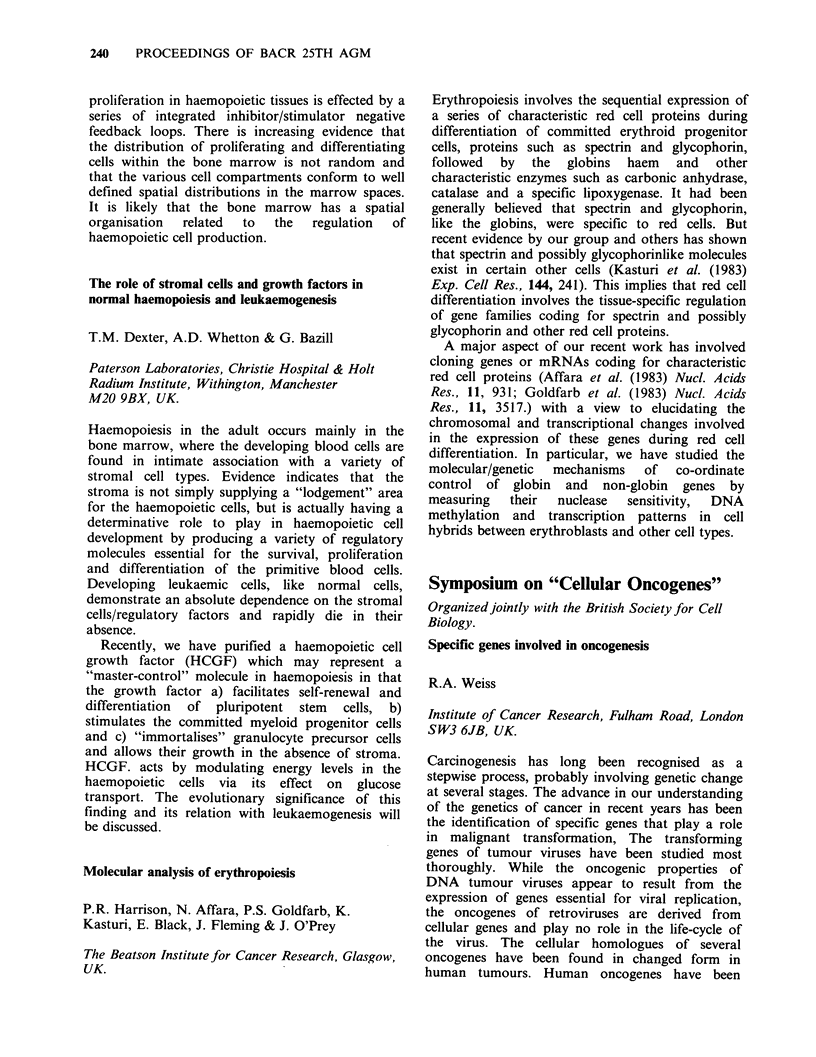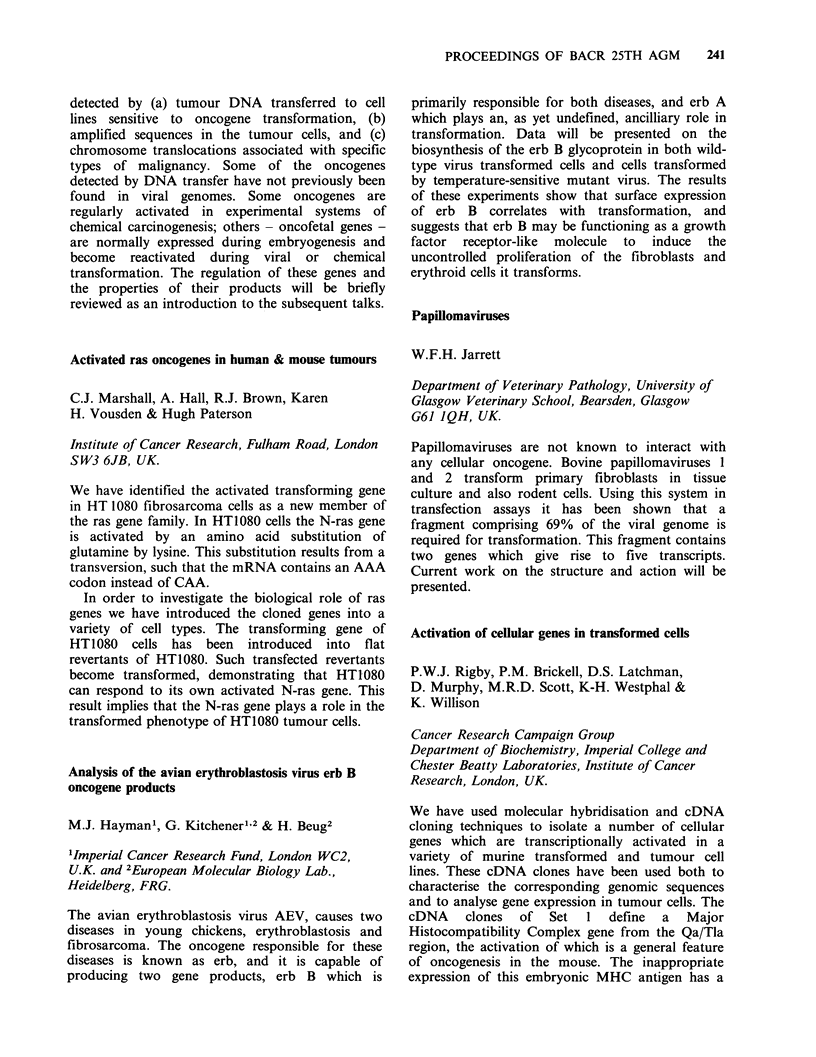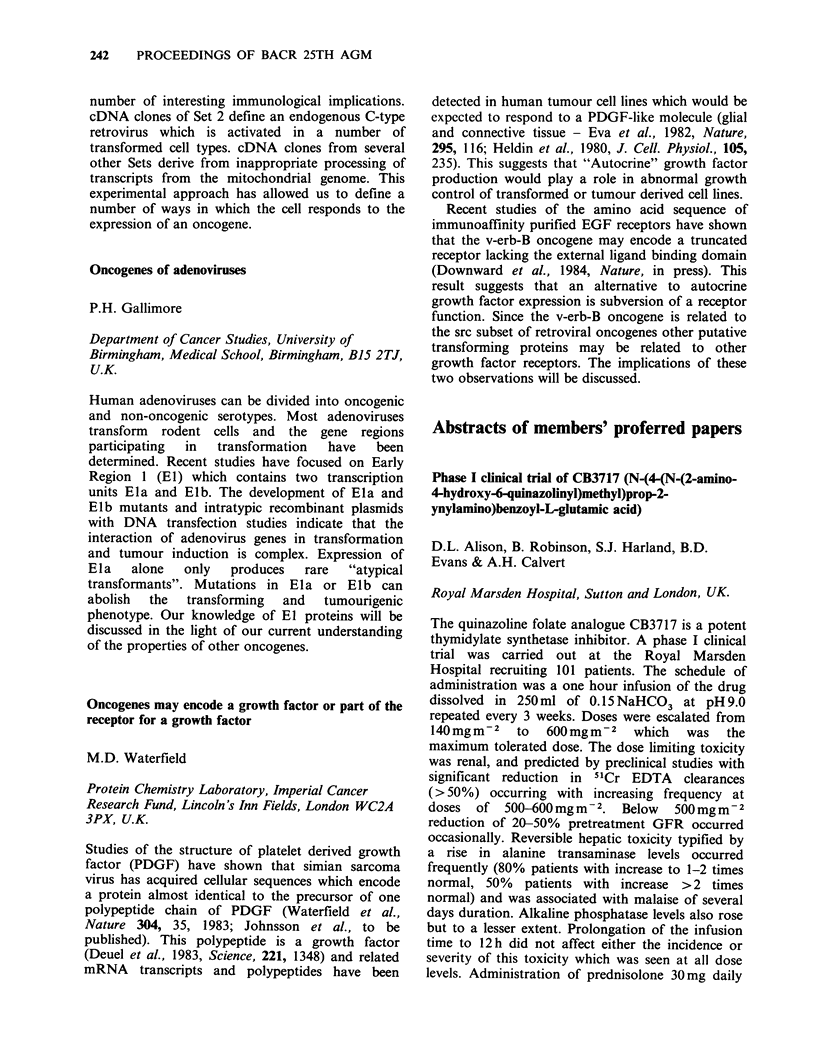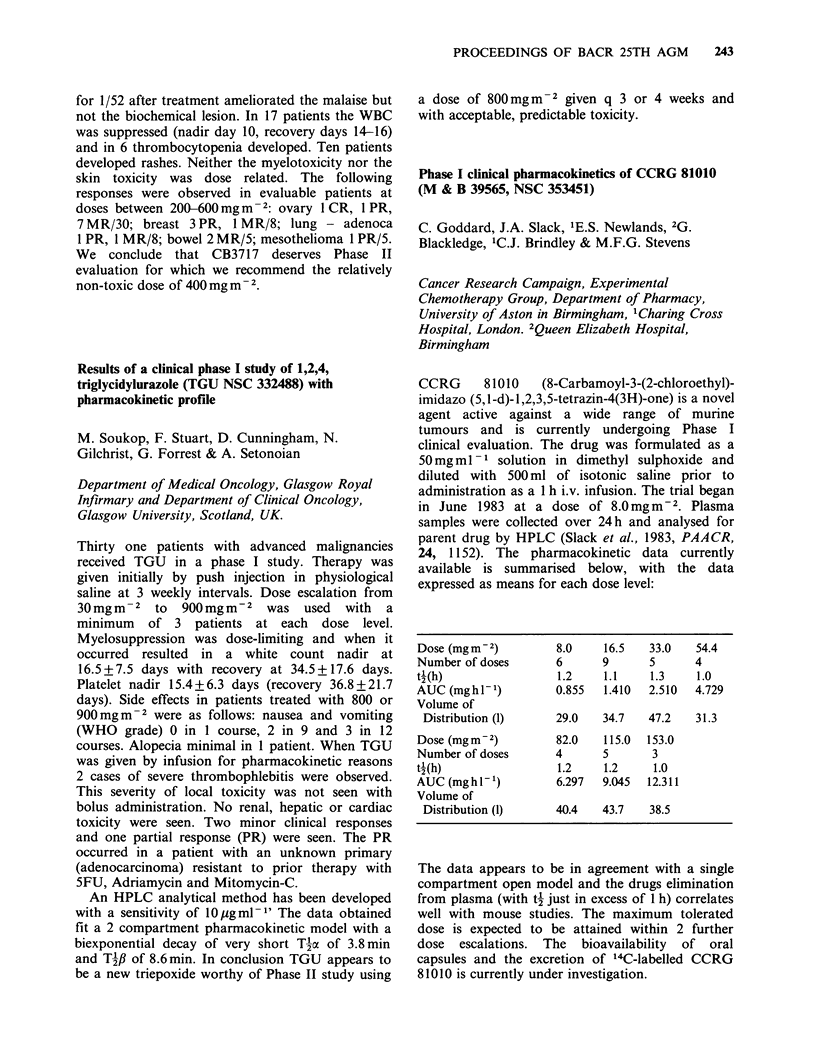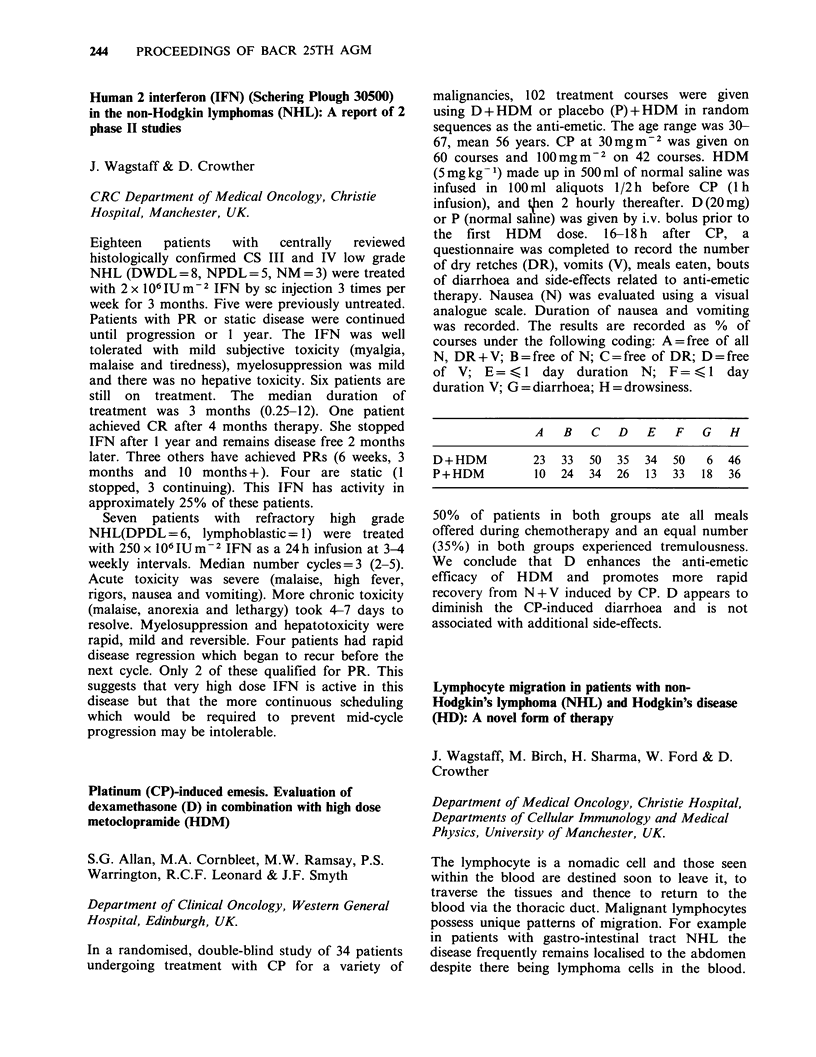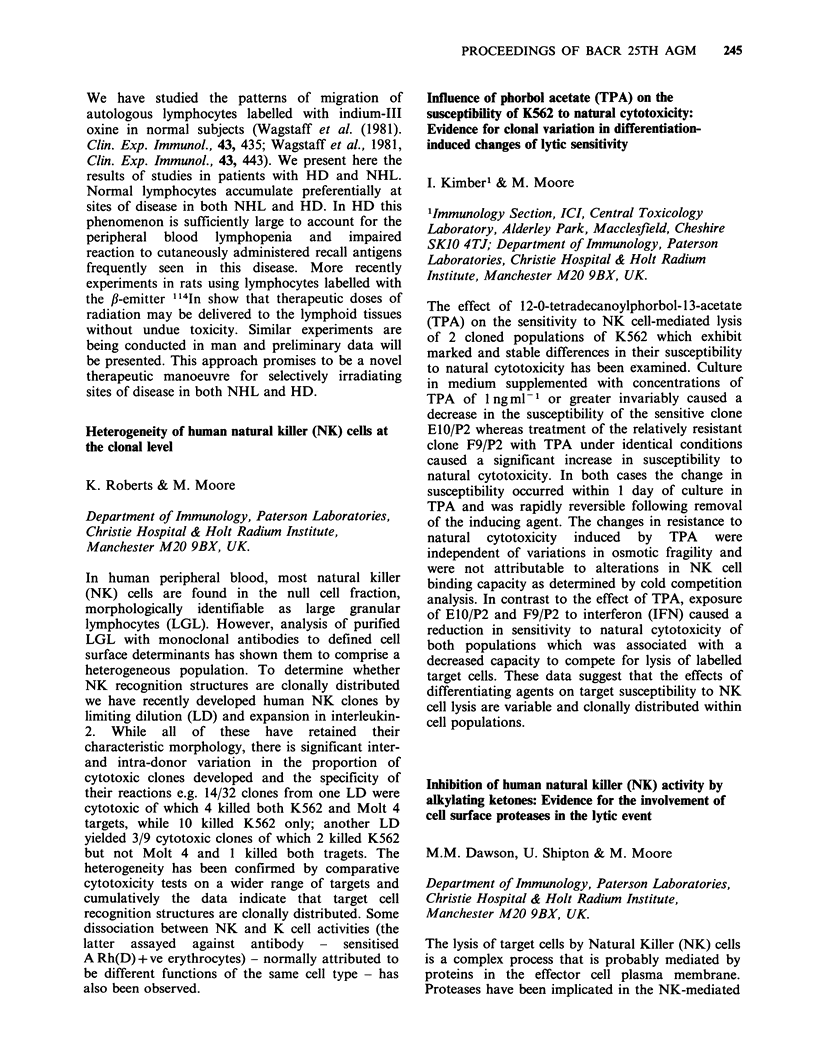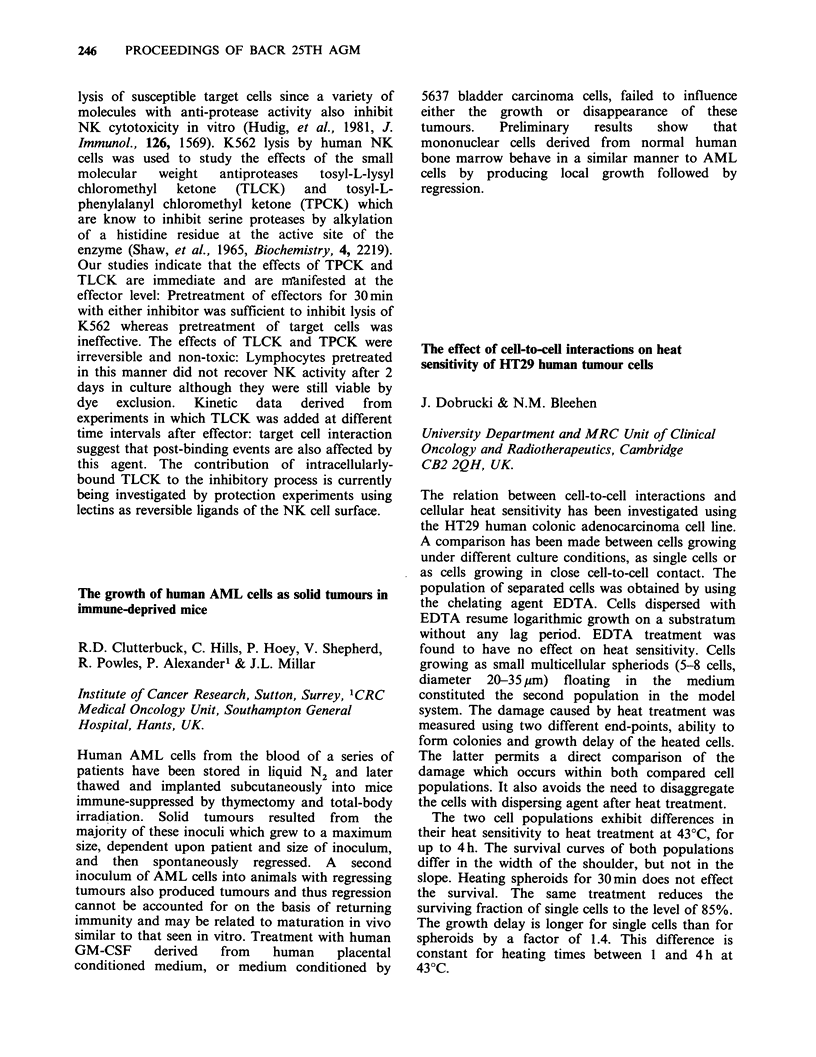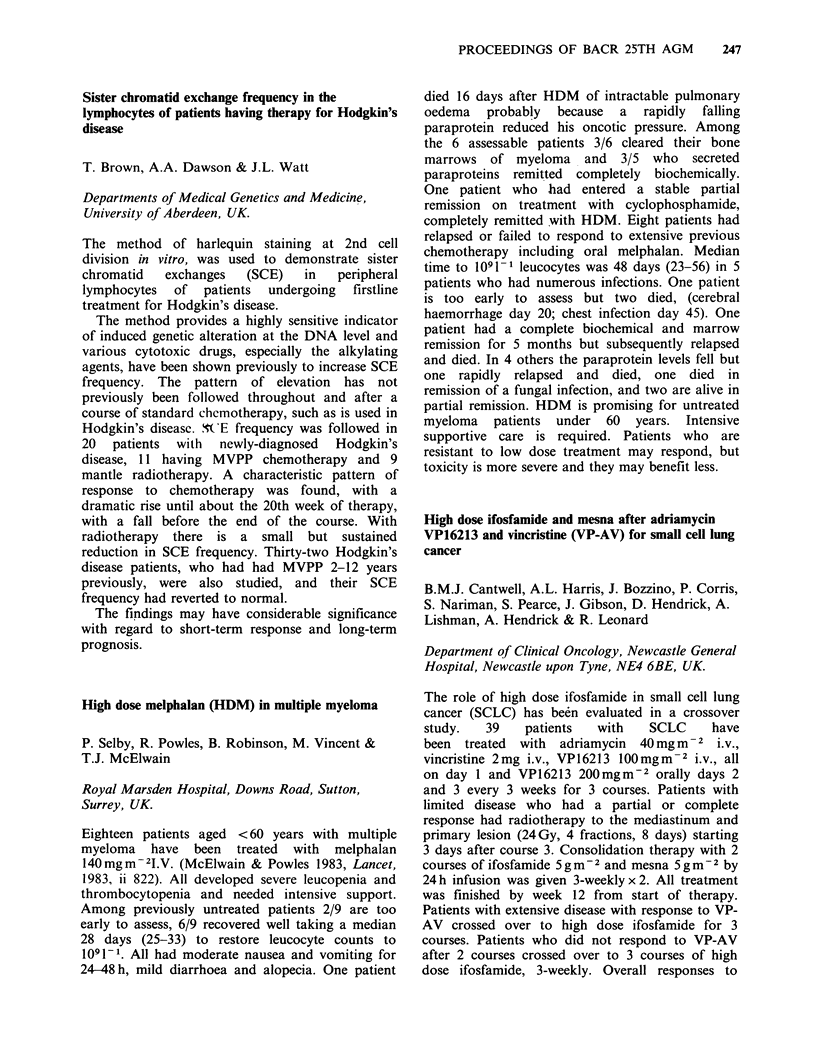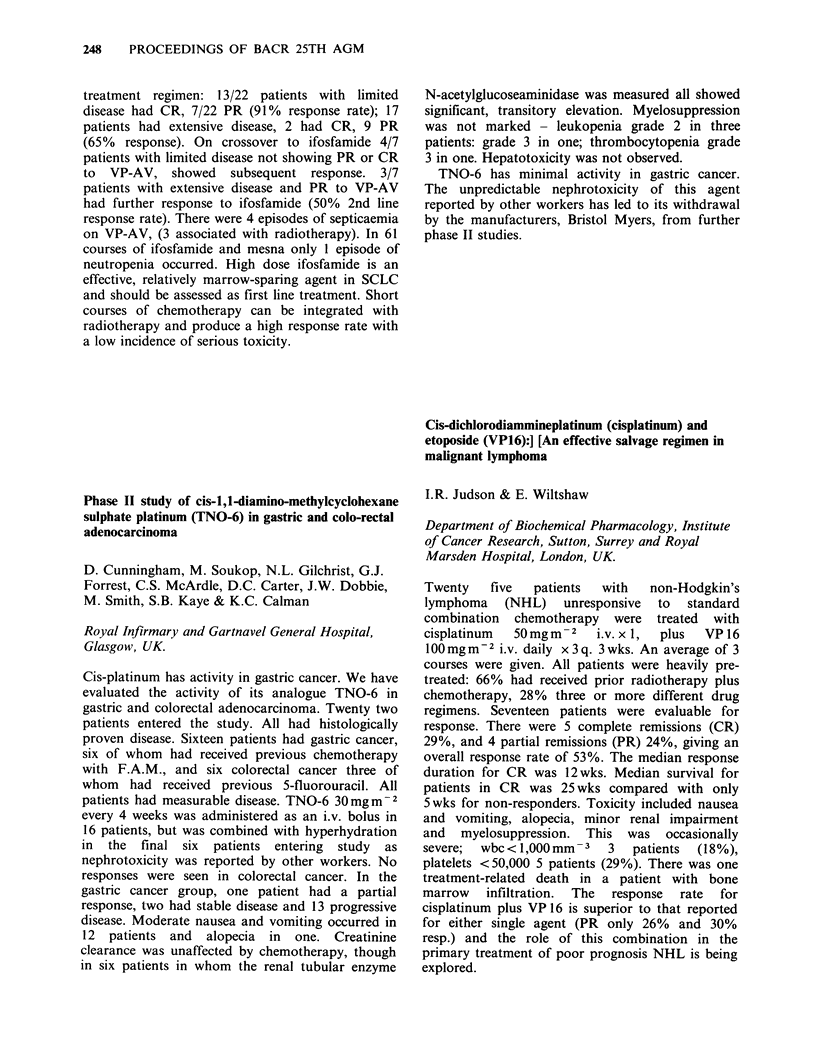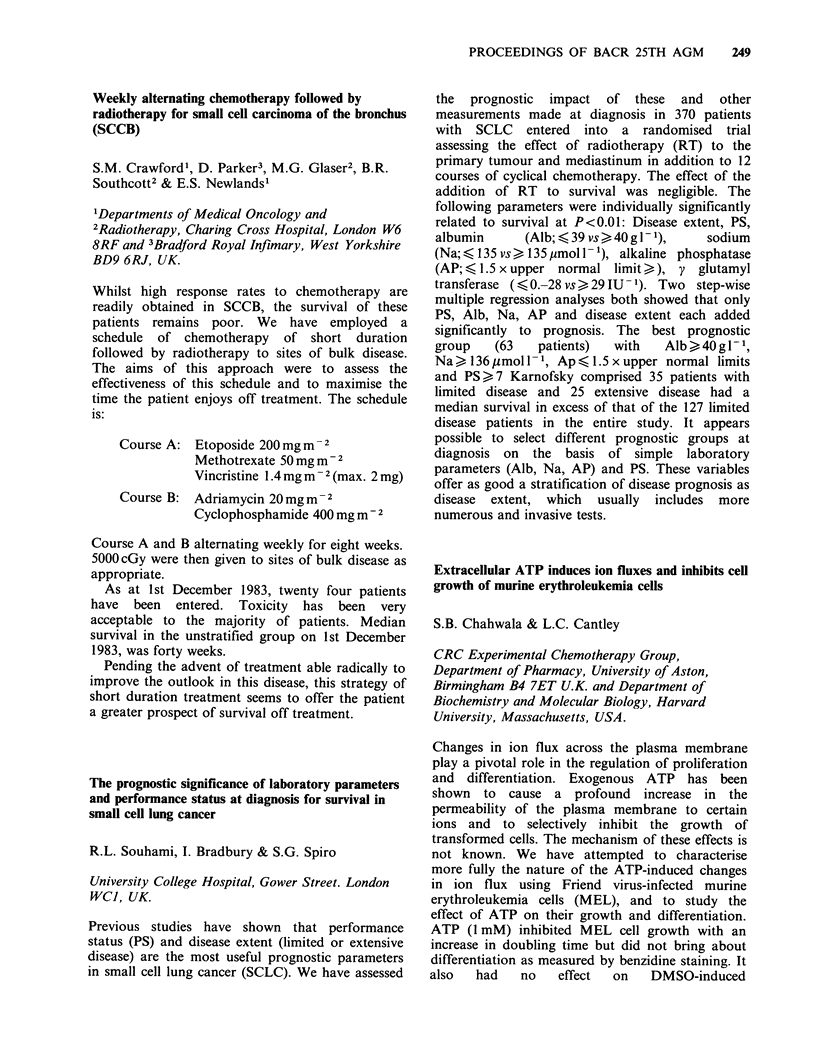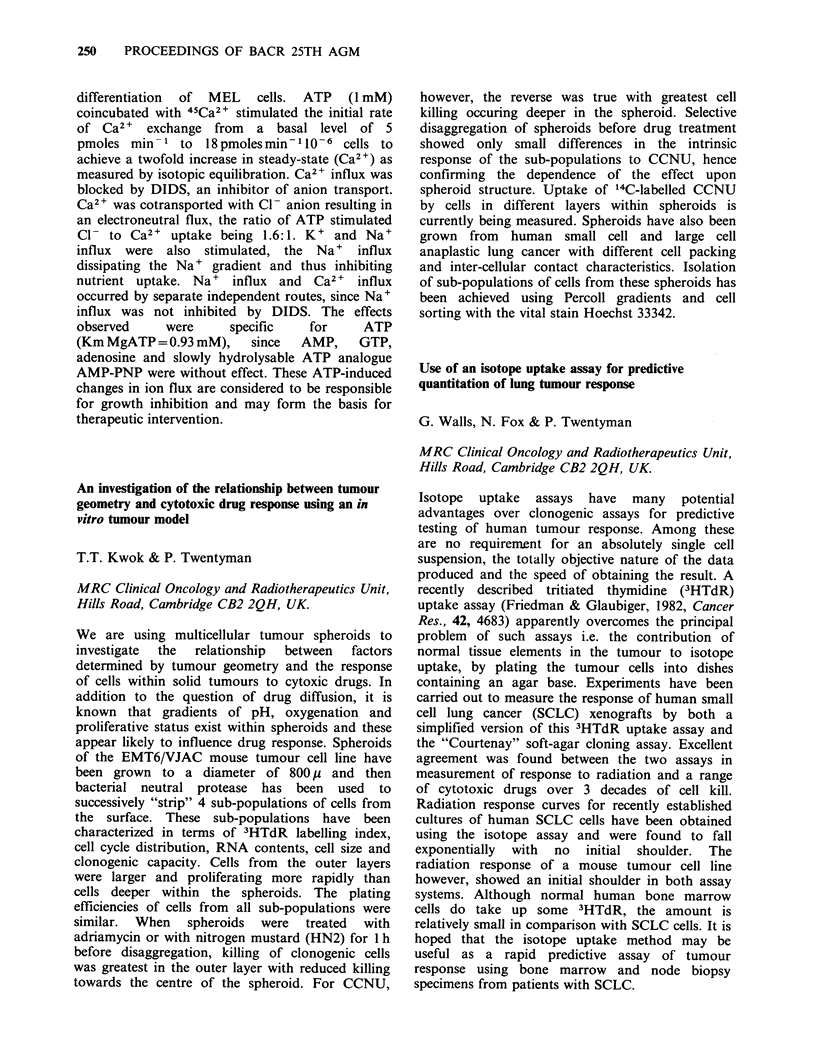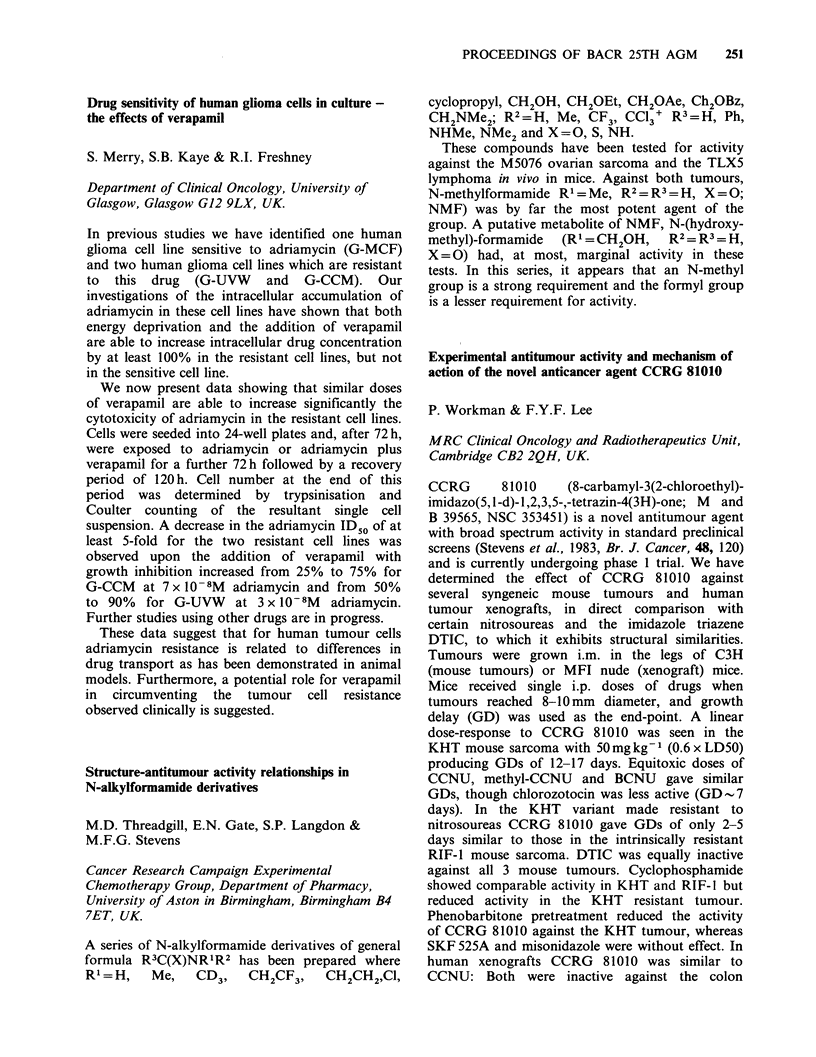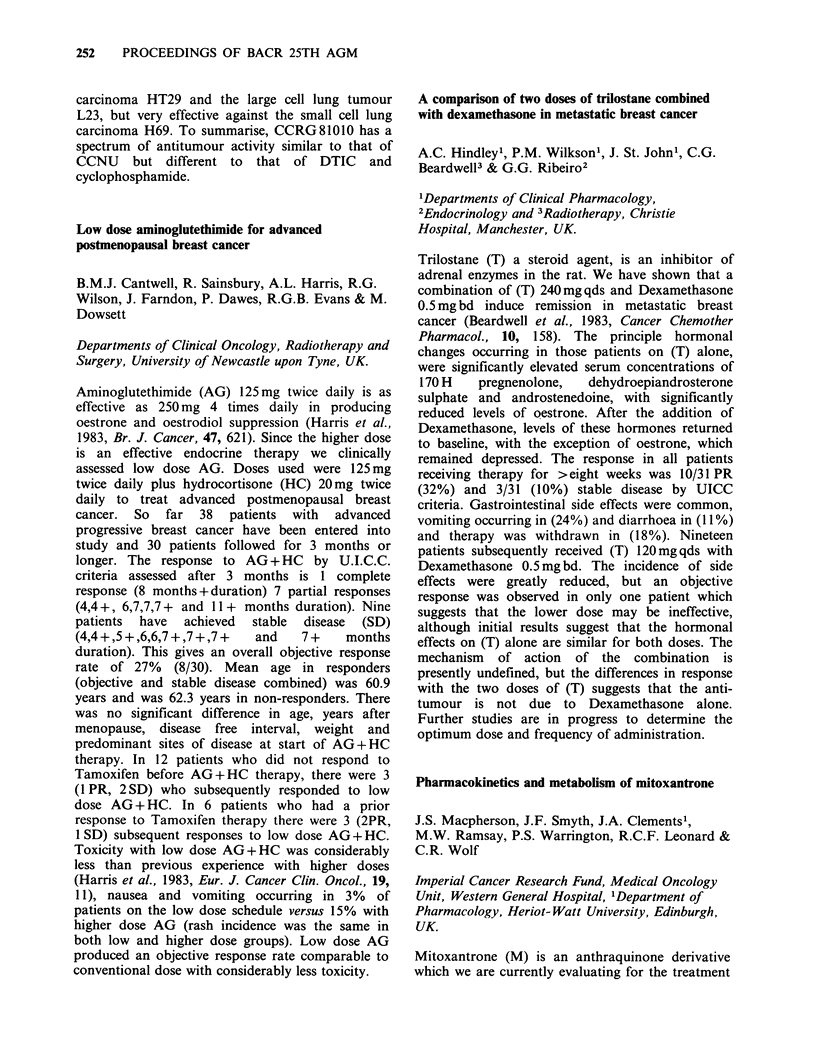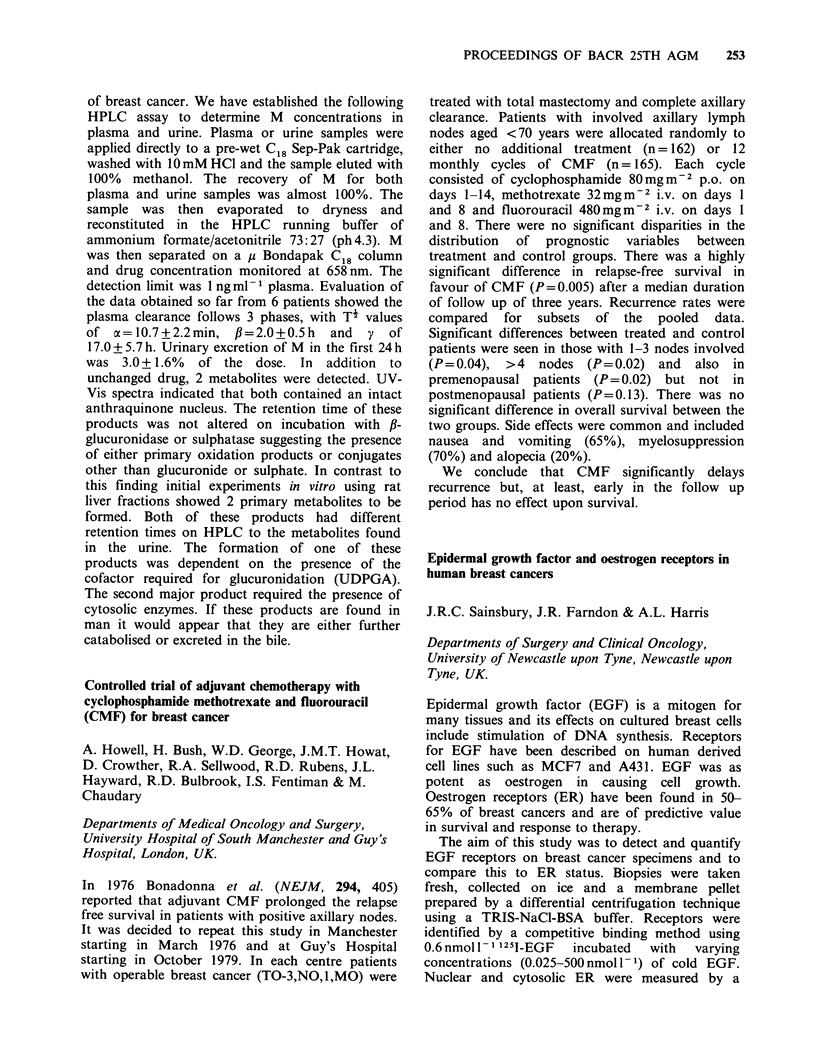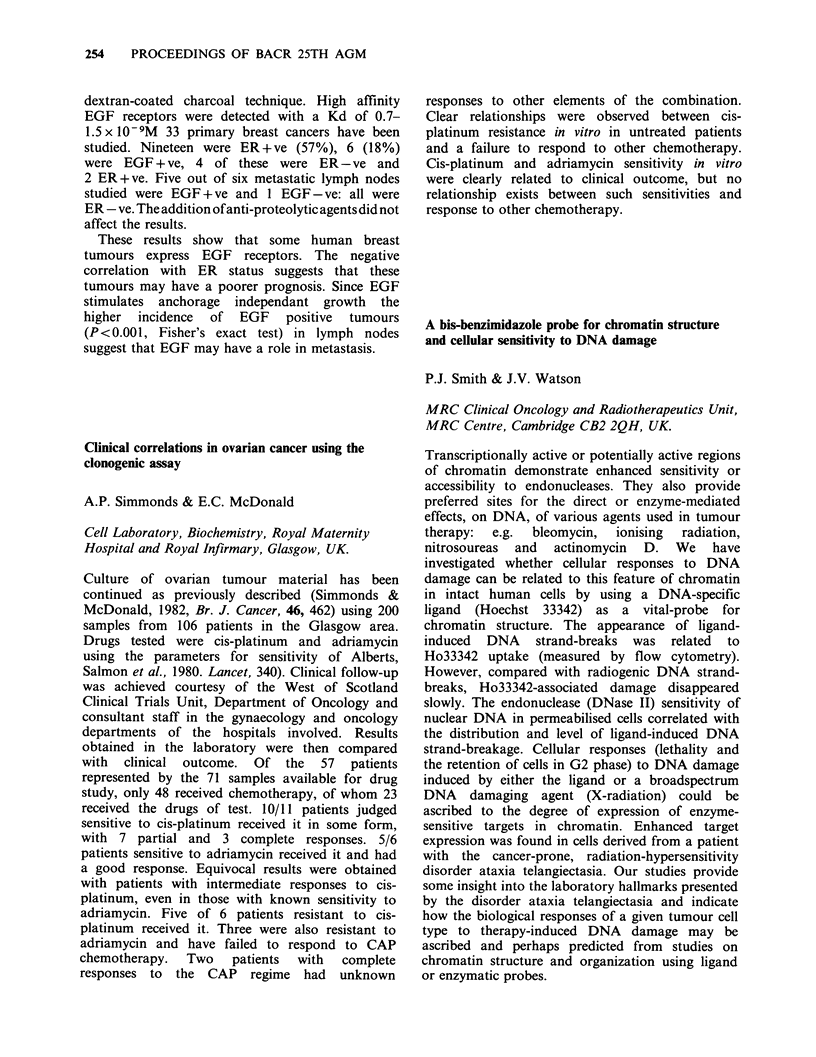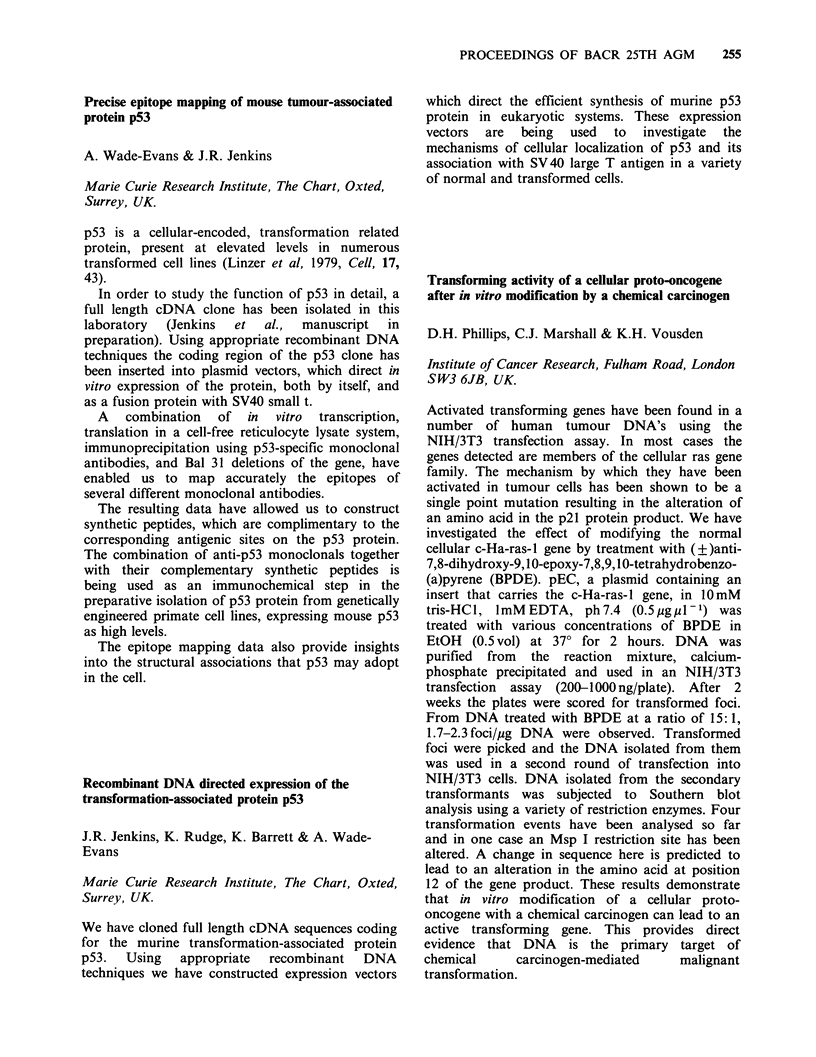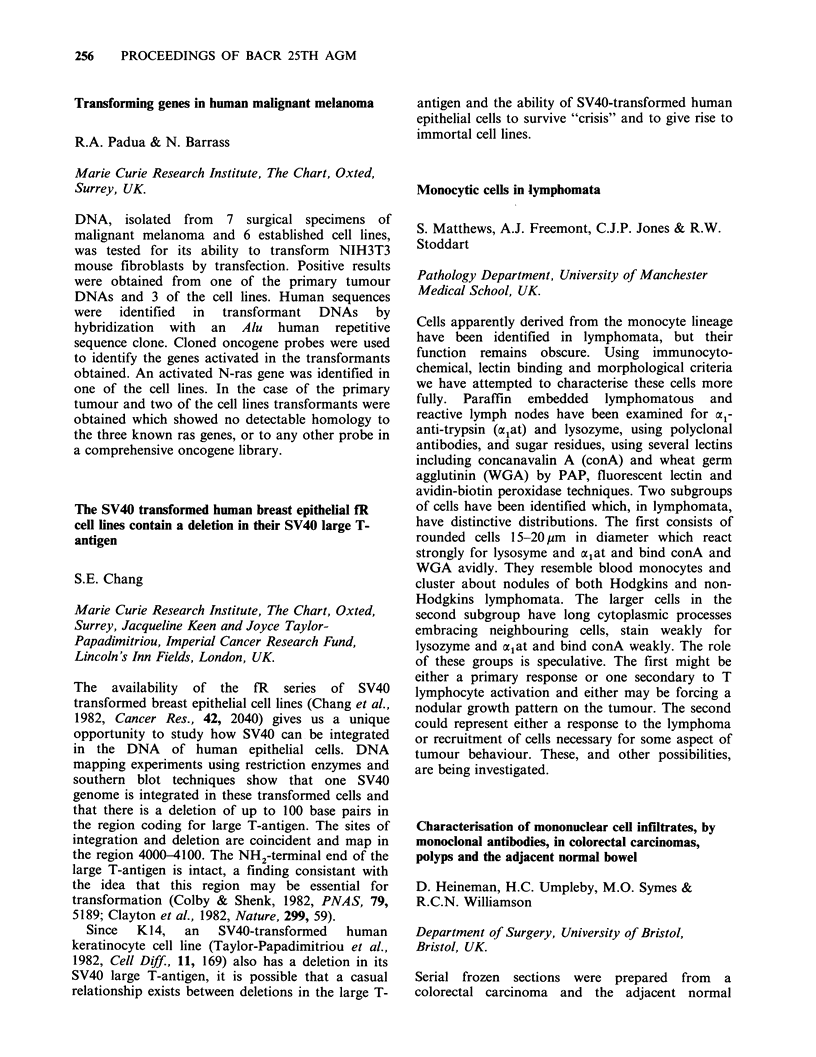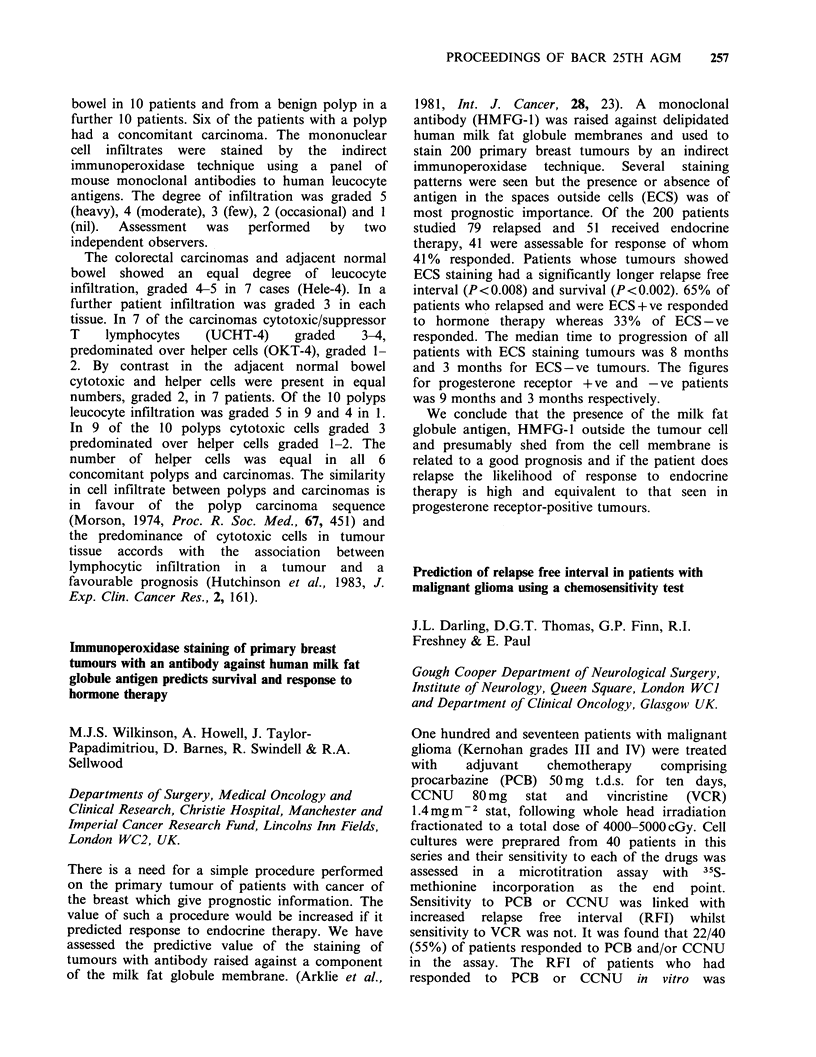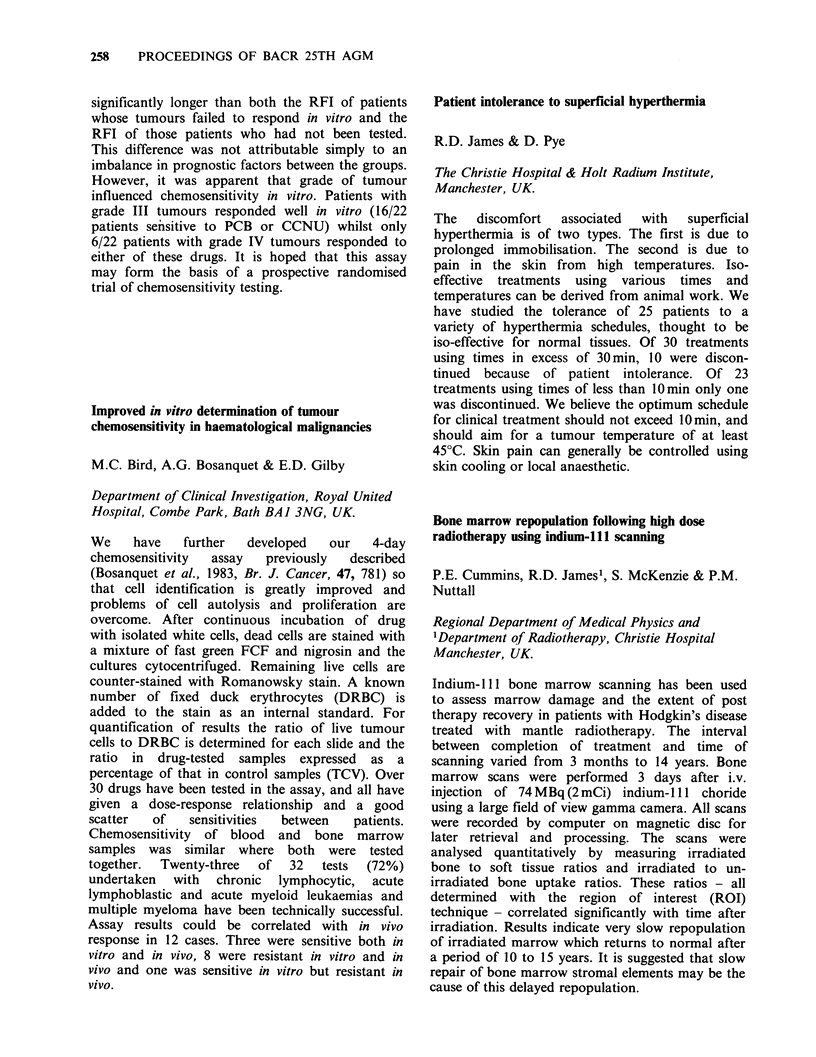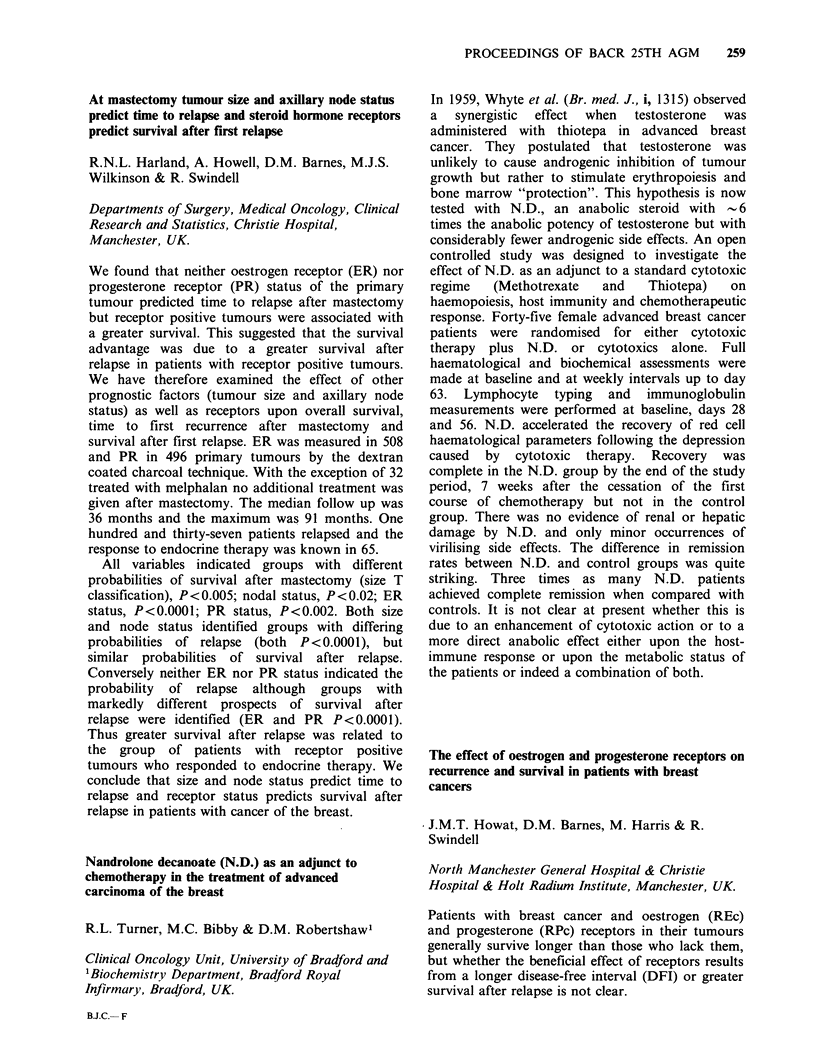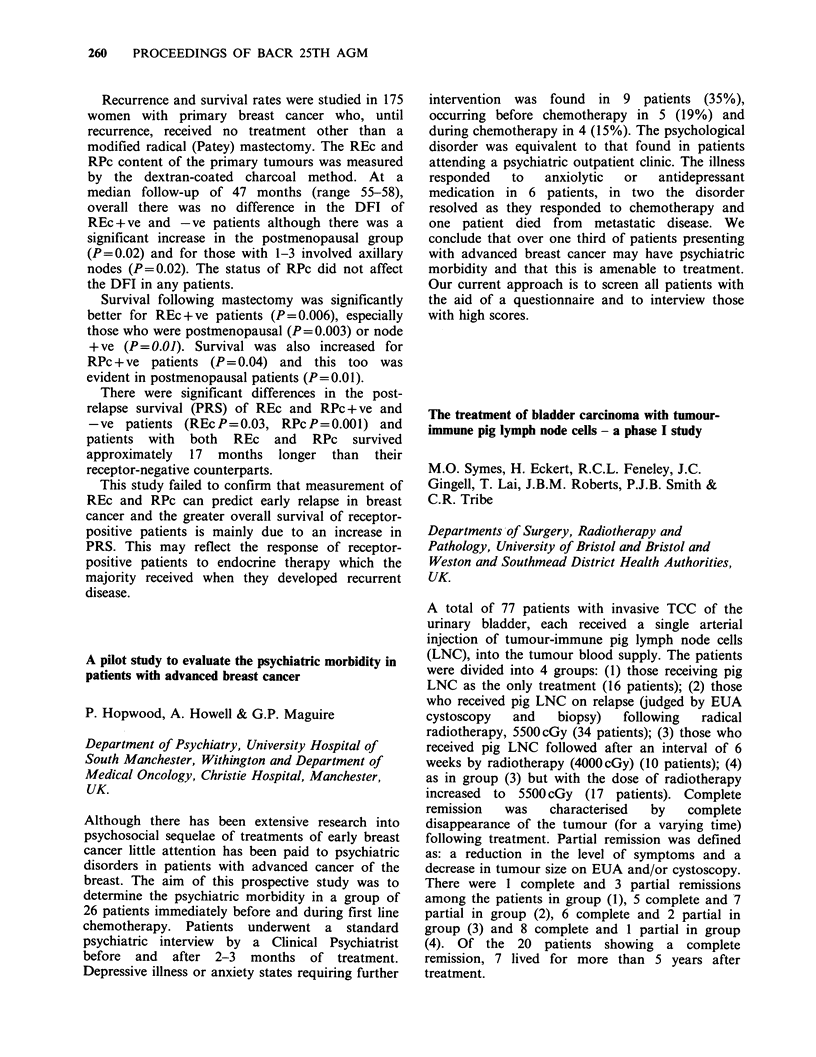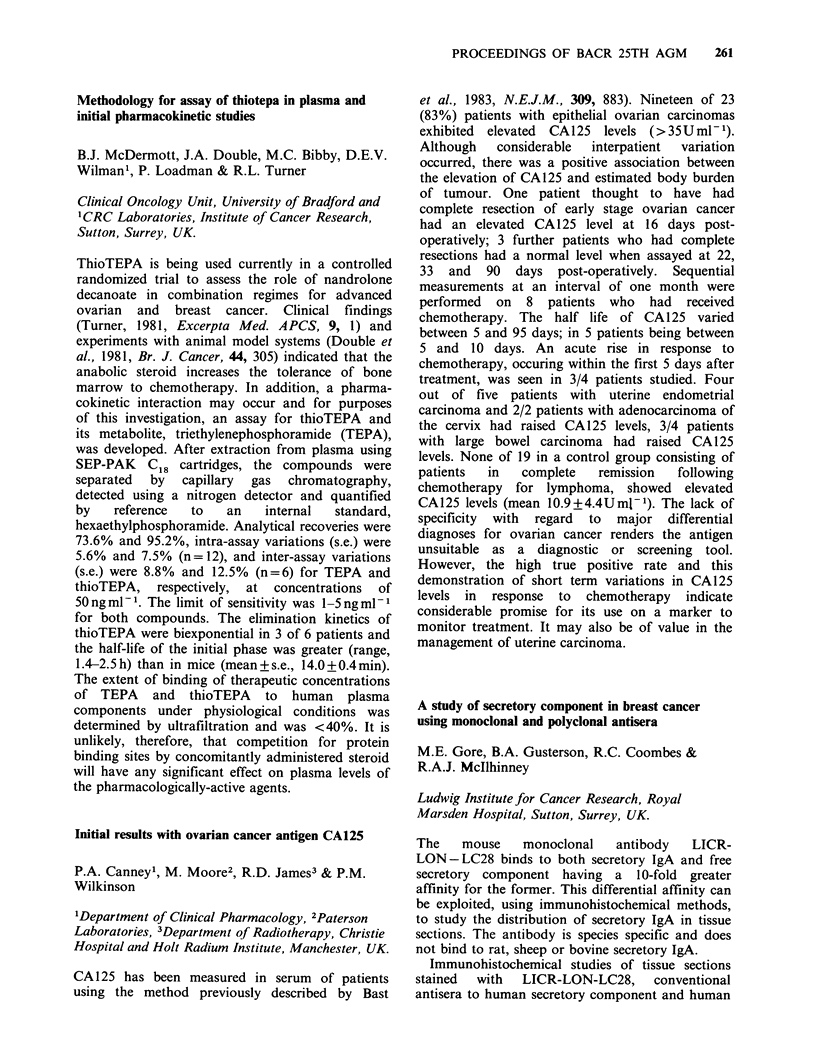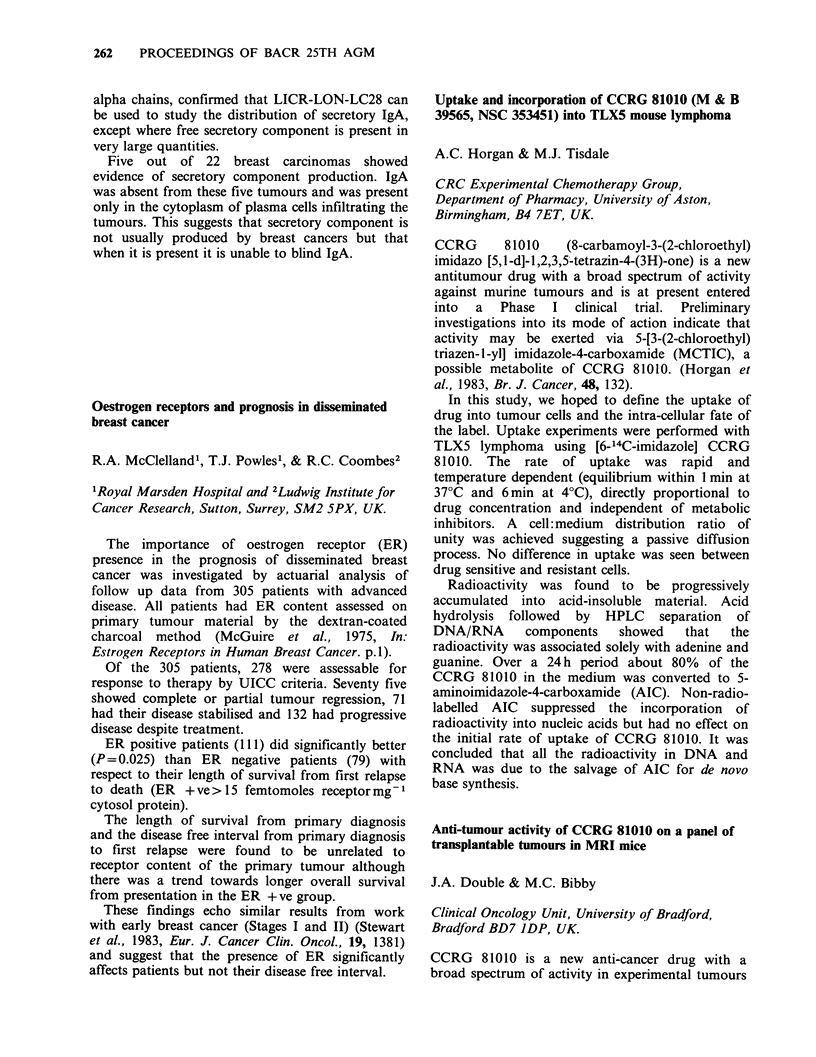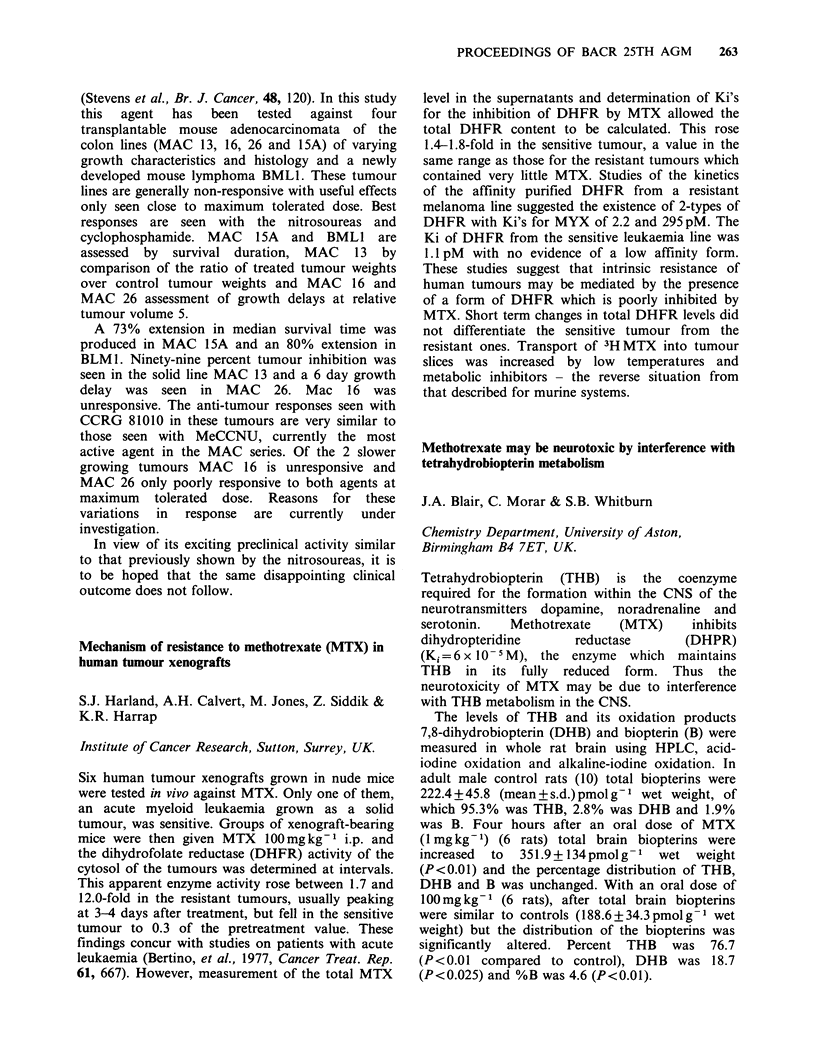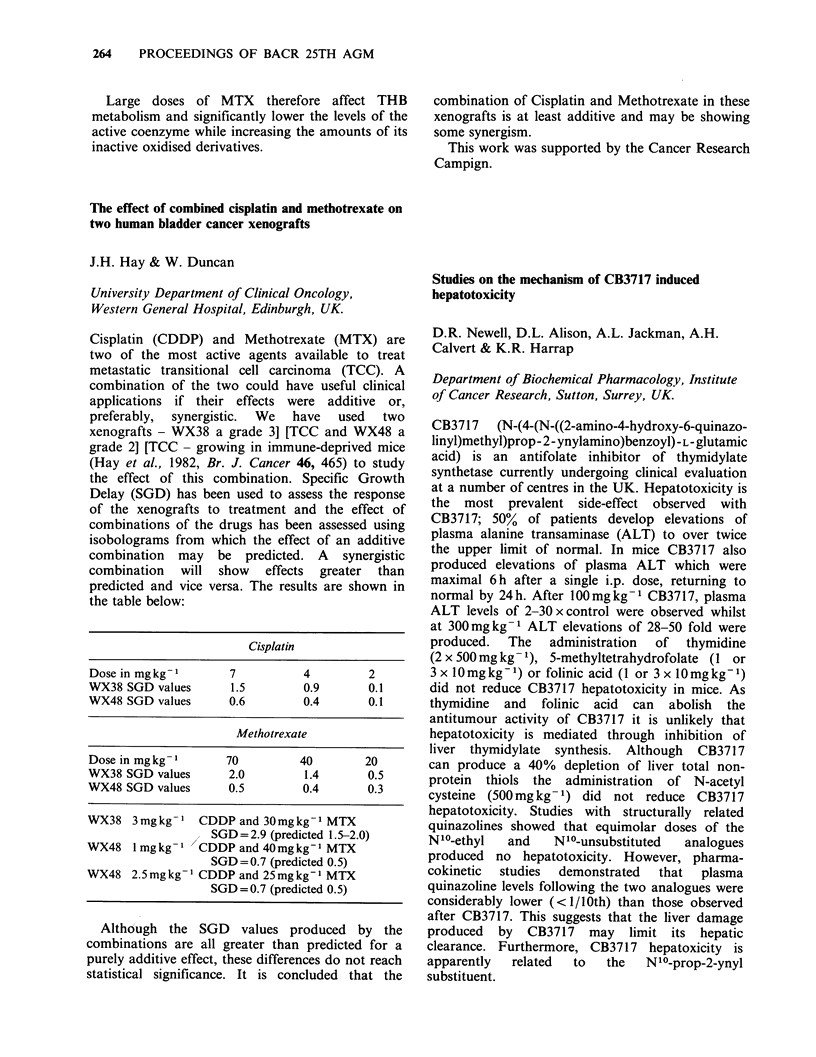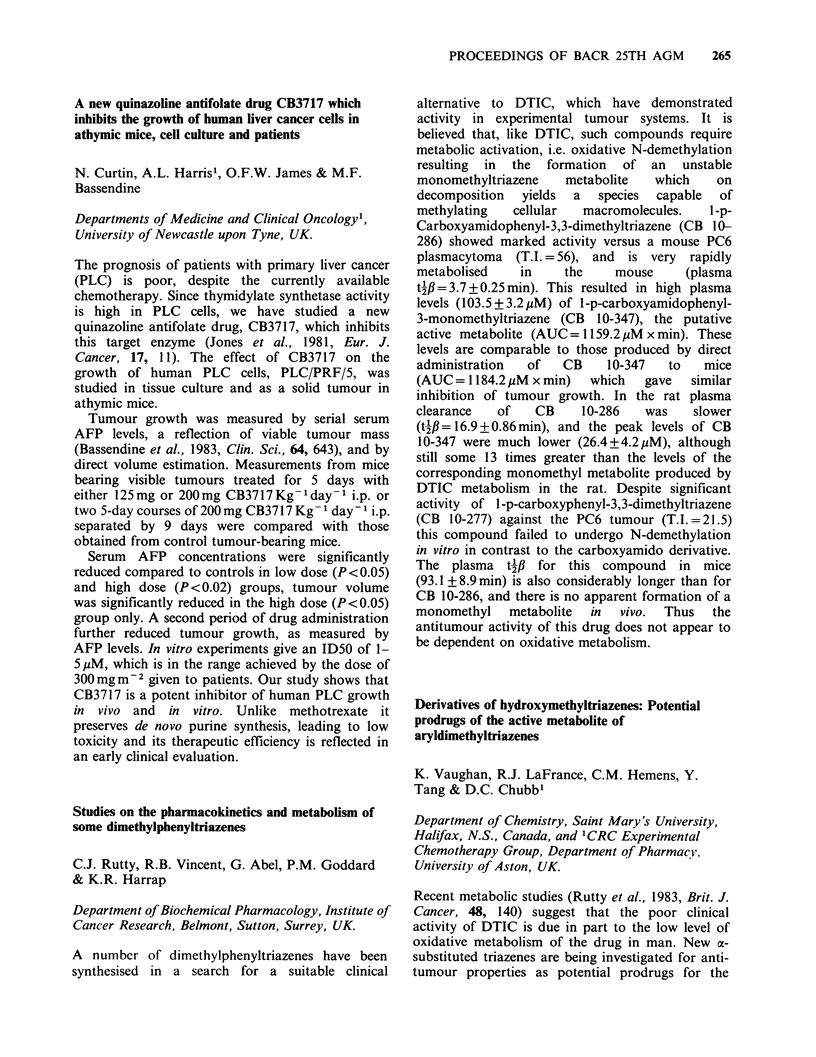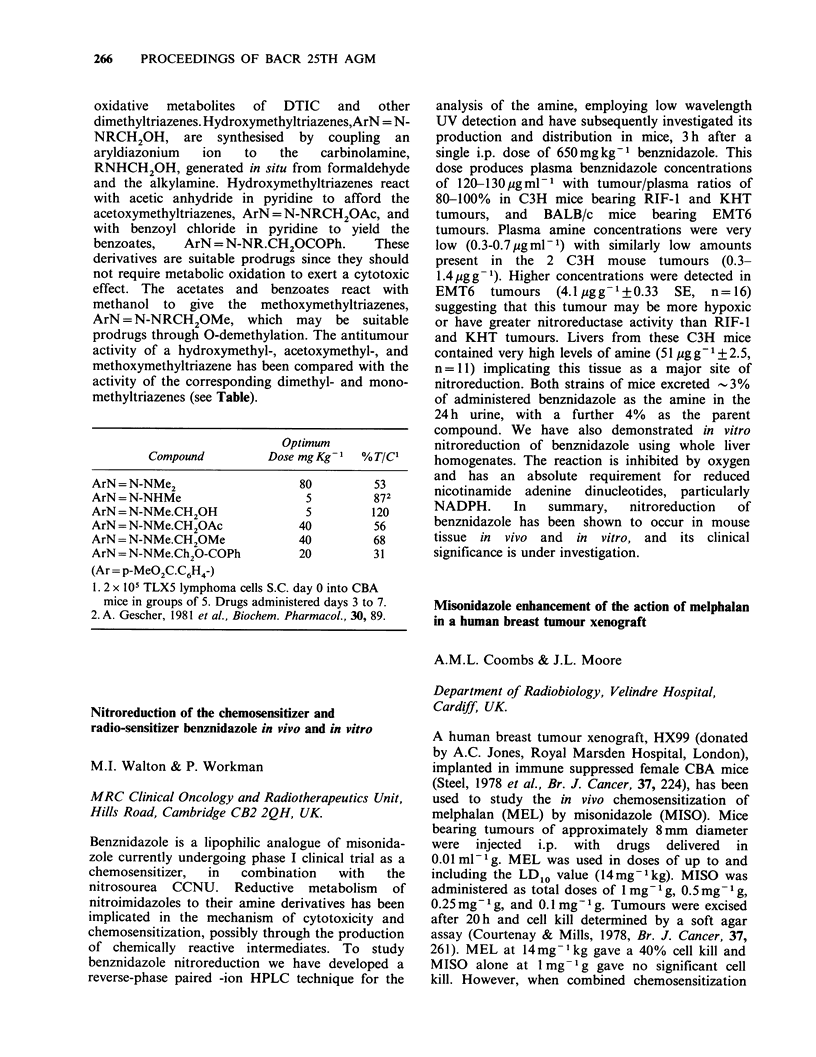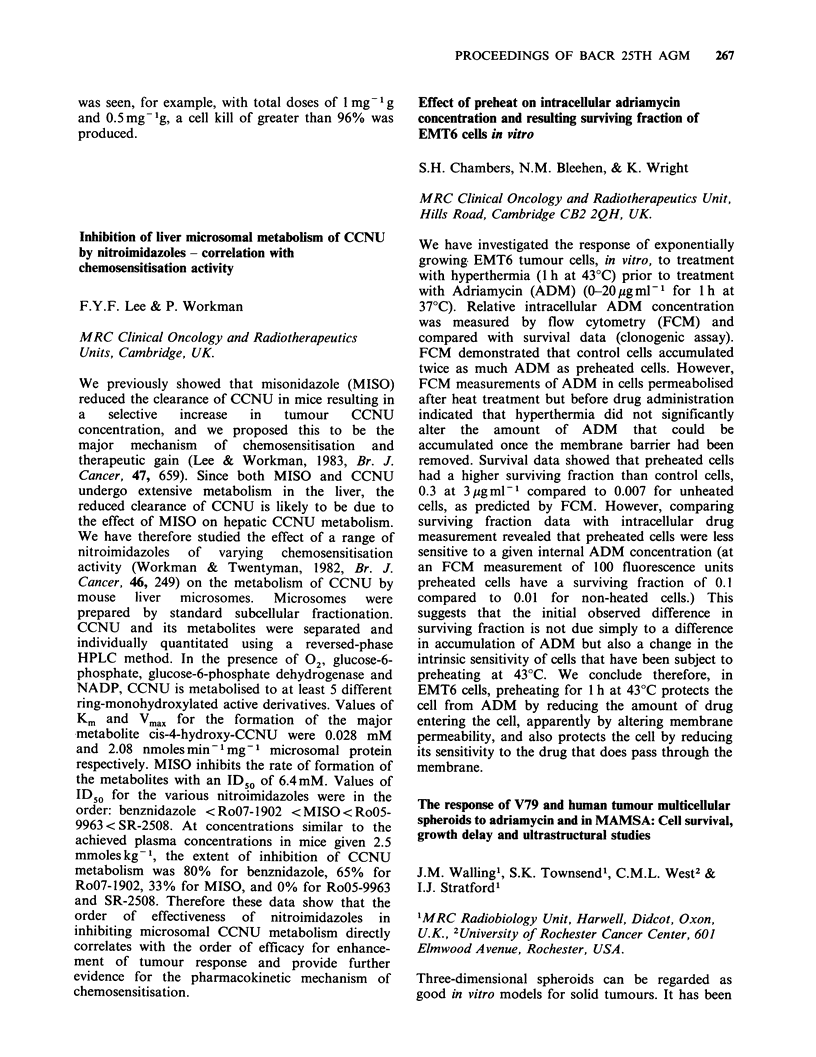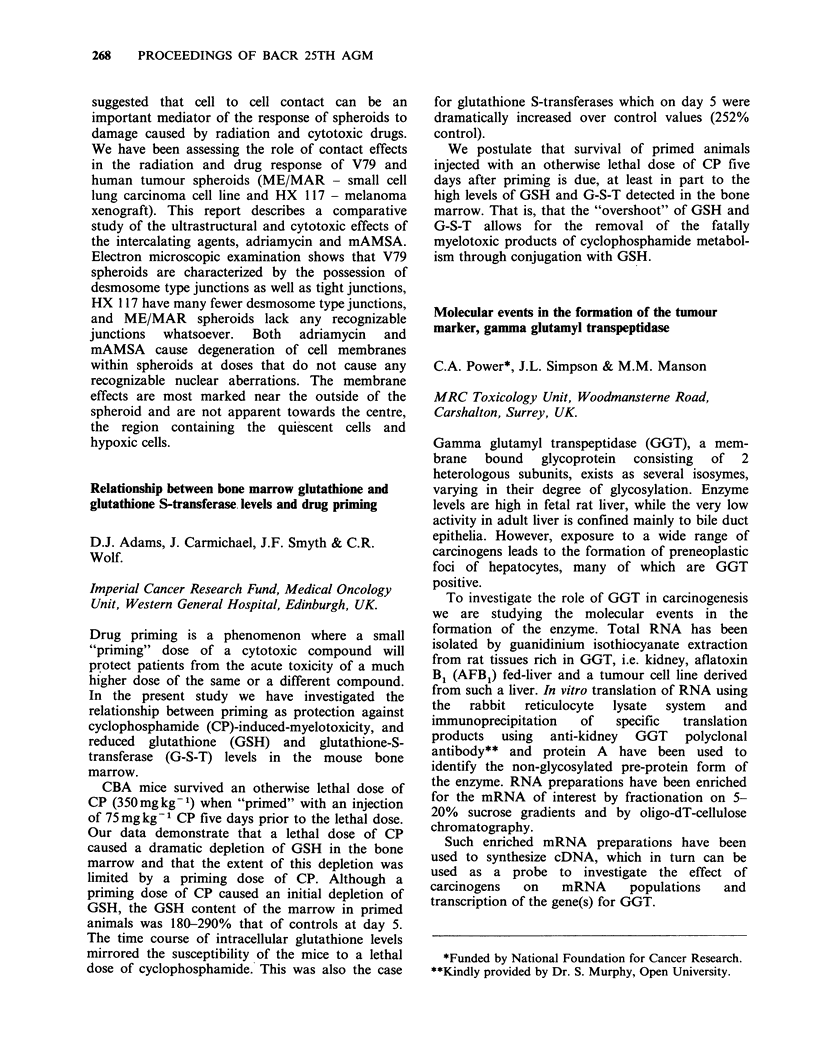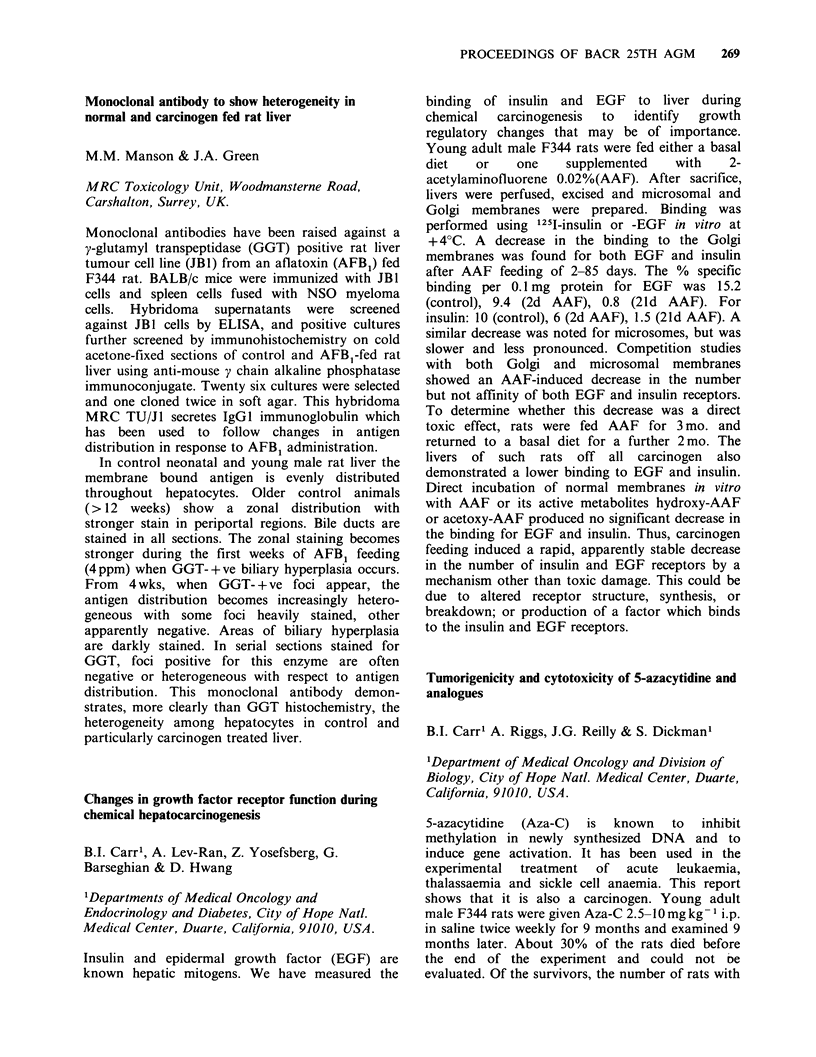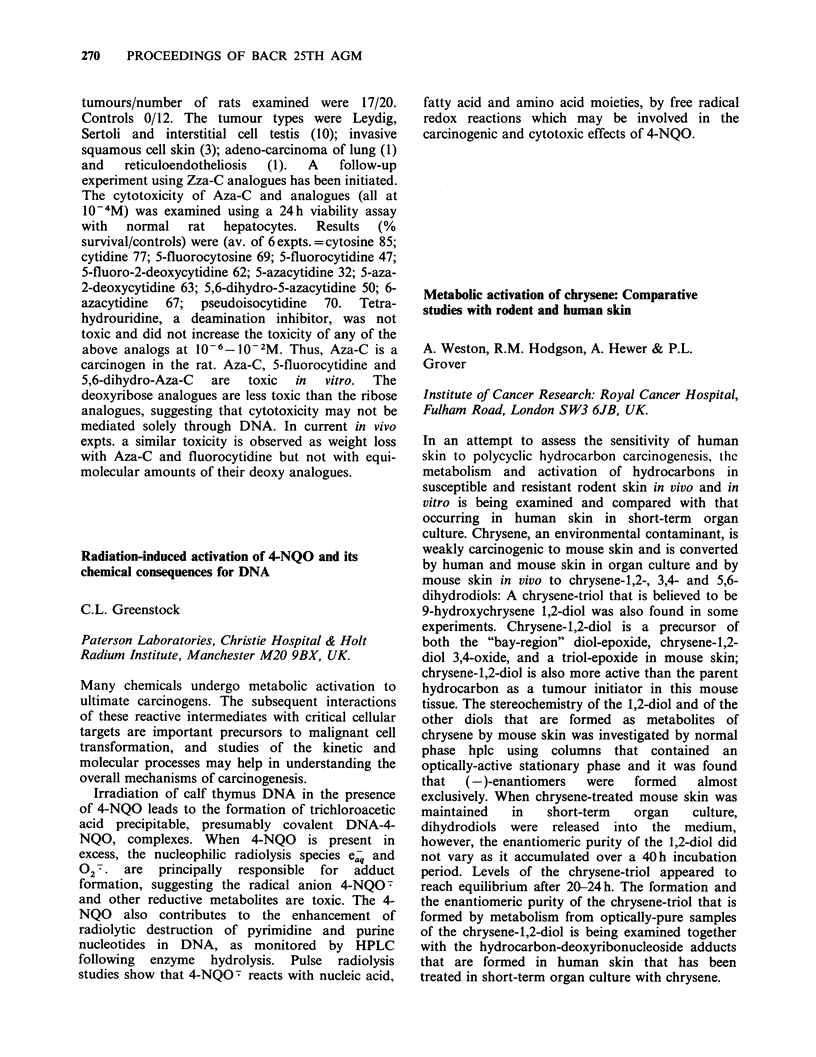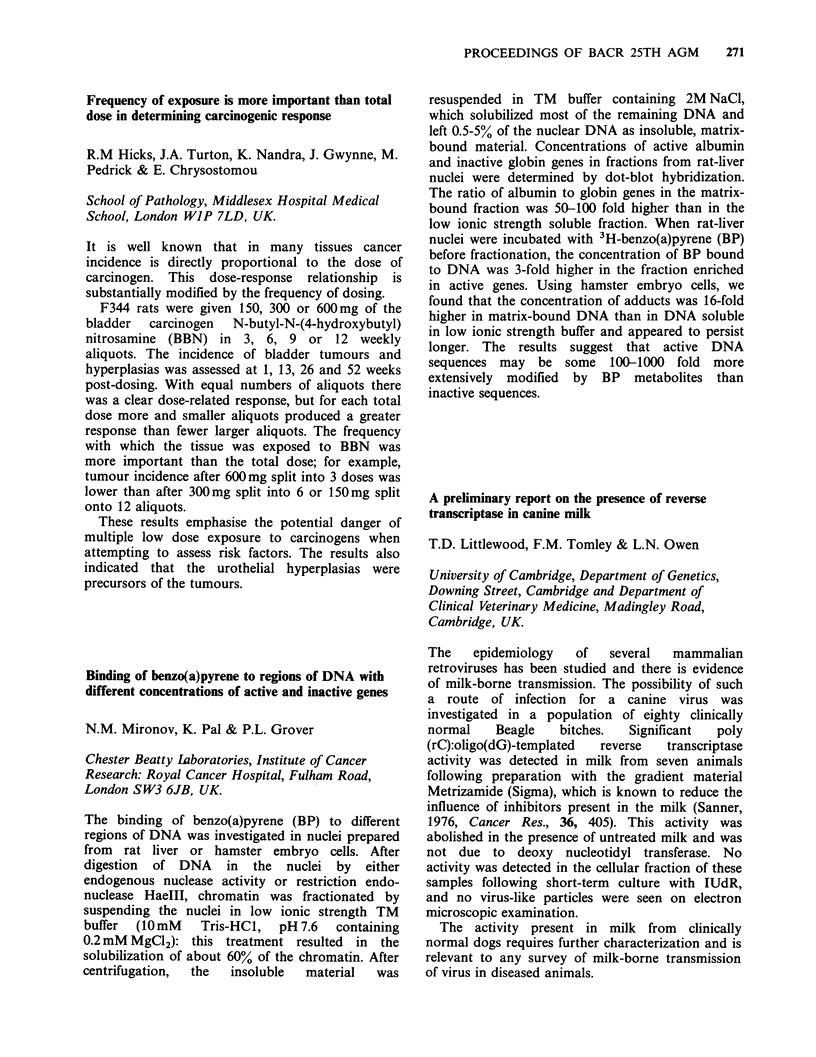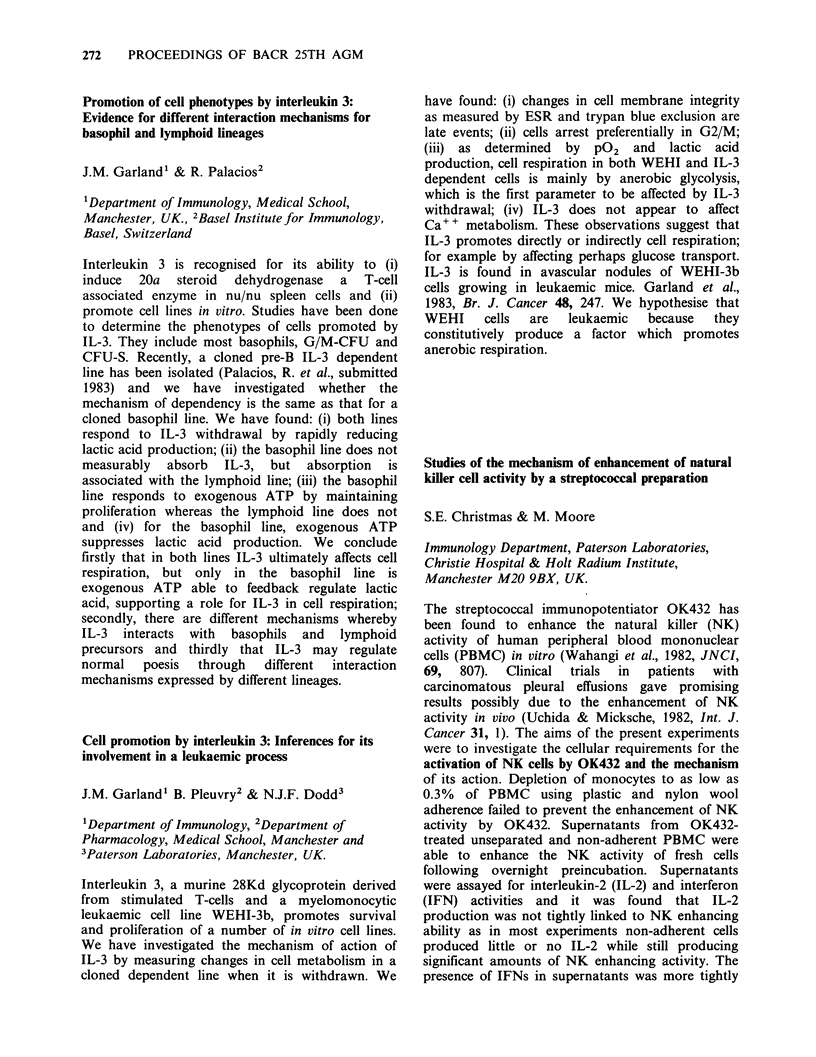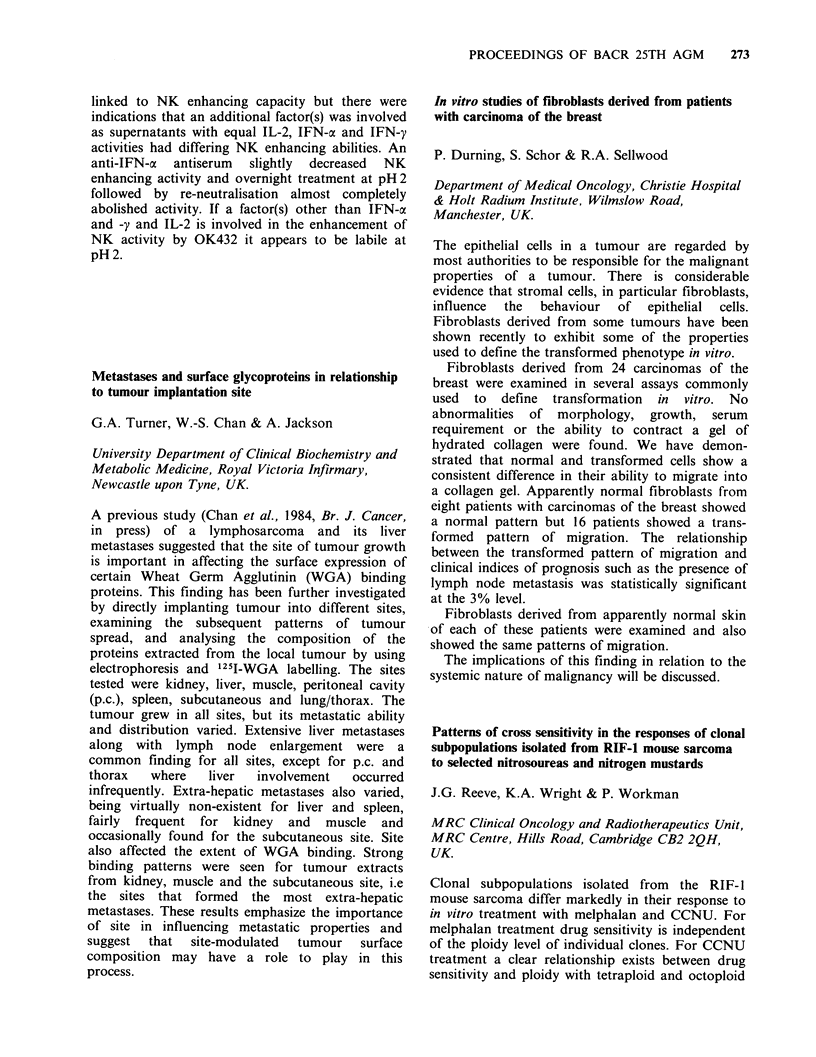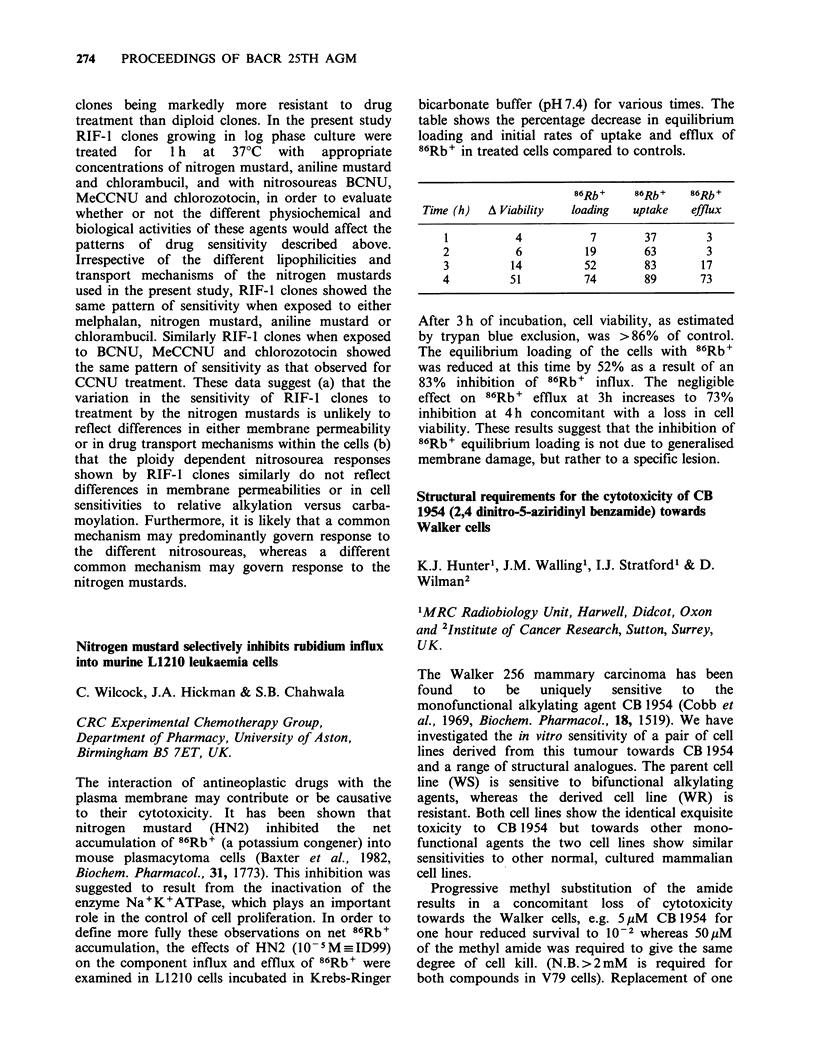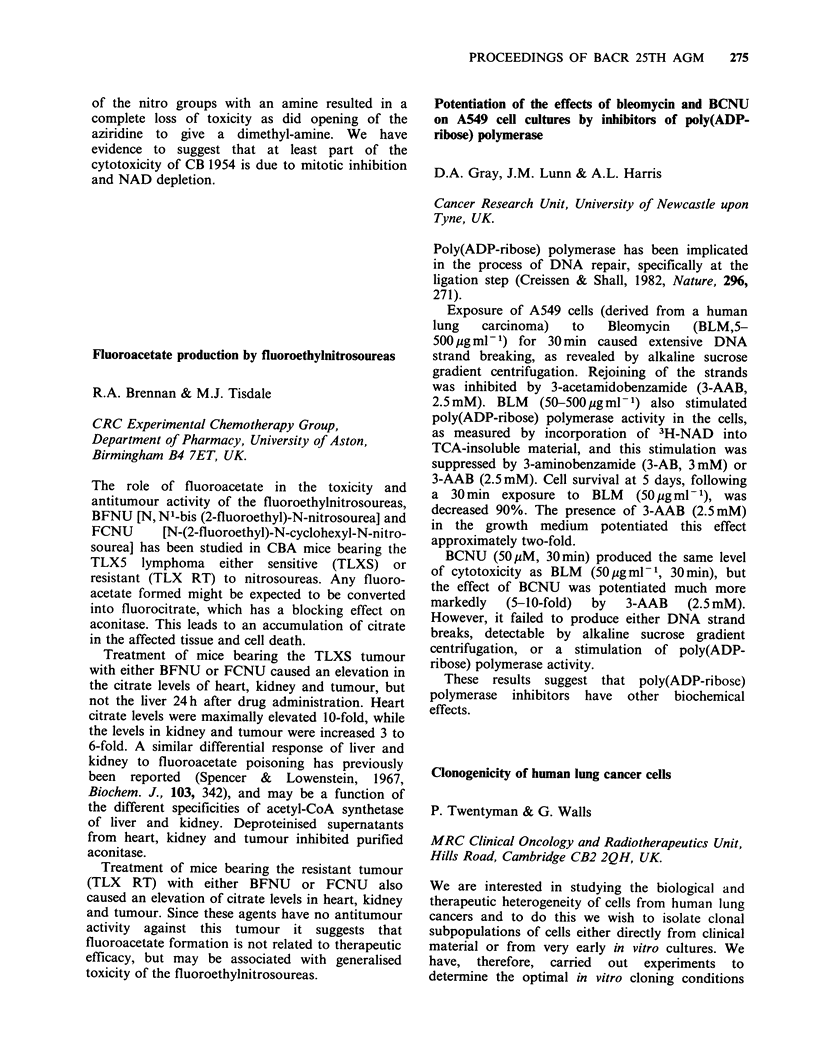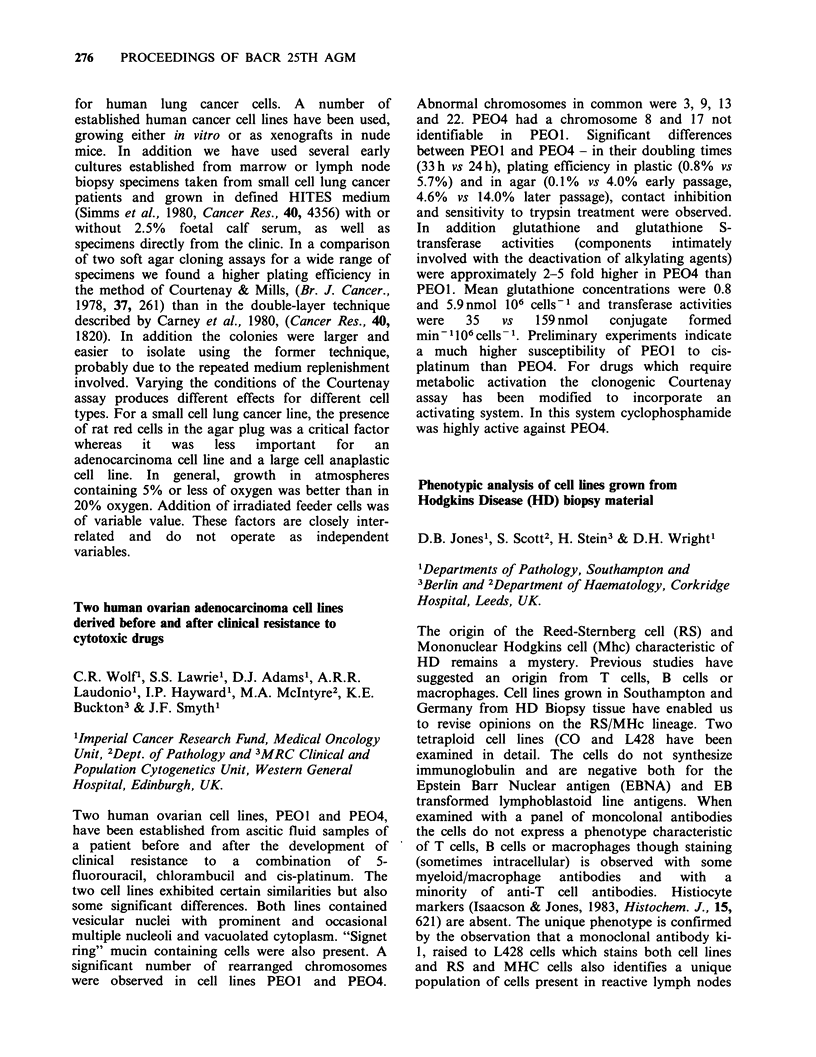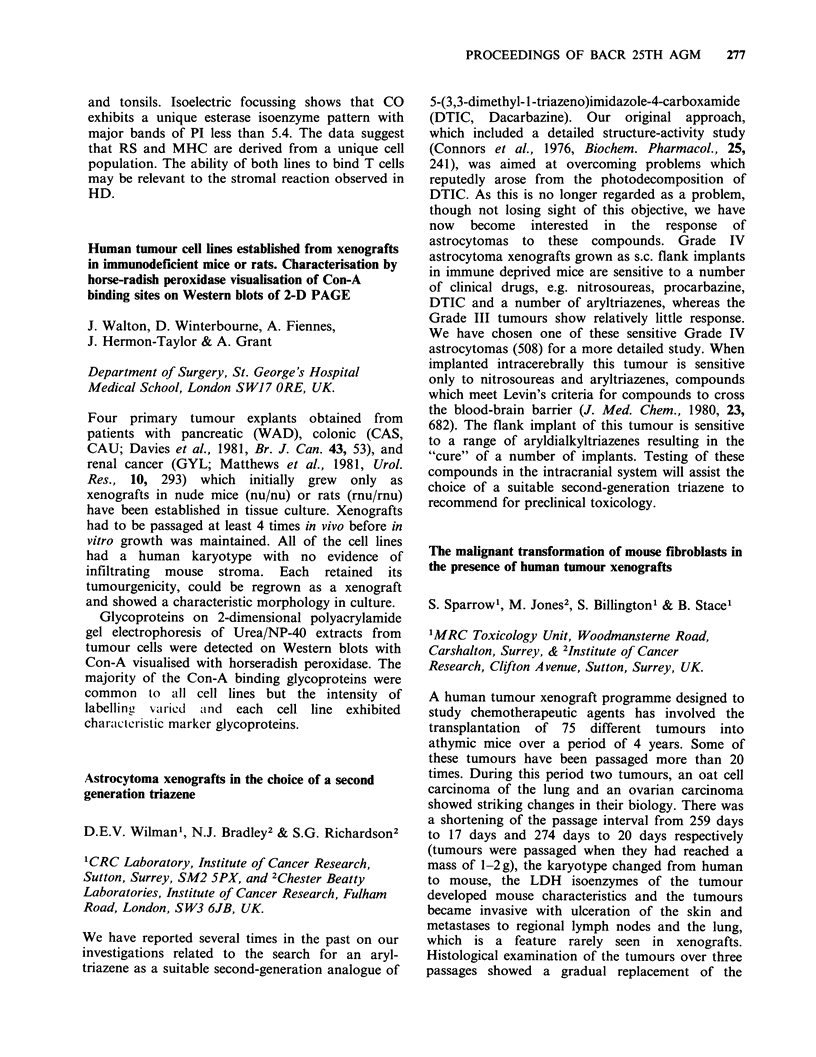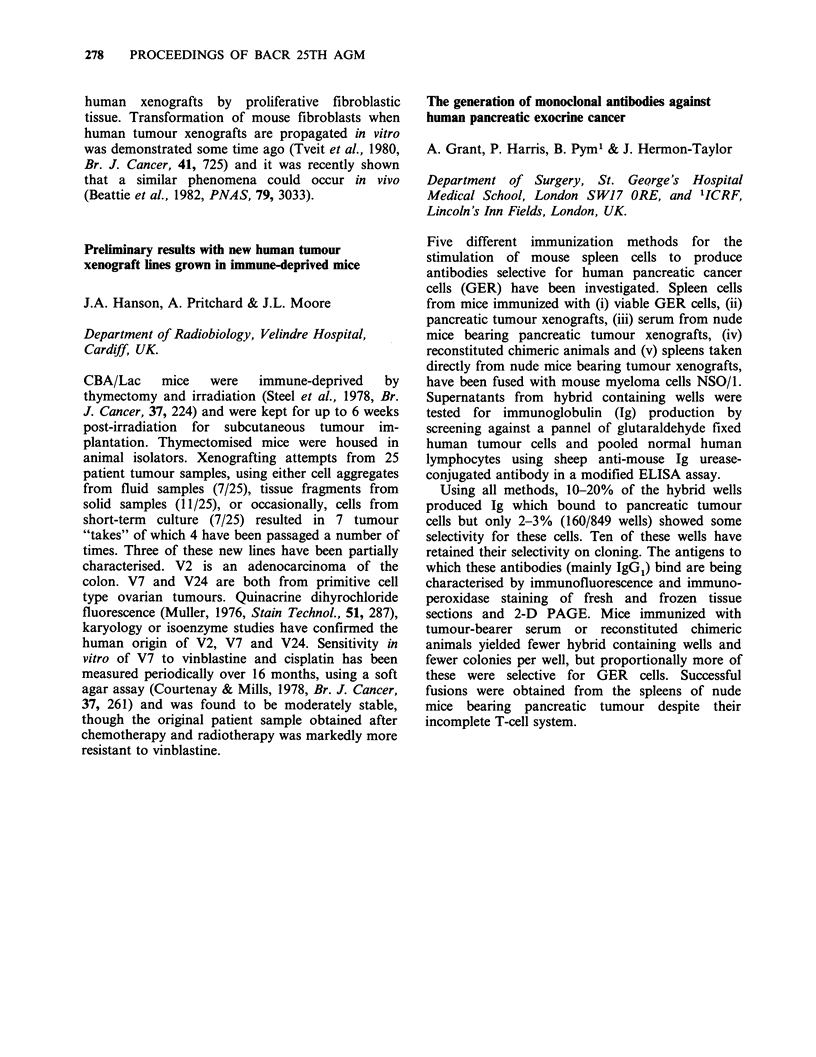# Twenty-fifth annual general meeting of the British Association for Cancer Research. Abstracts of invited and profferred papers.

**DOI:** 10.1038/bjc.1984.170

**Published:** 1984-08

**Authors:** 


Br. J. Cancer (1984), 50, 239–278

### Twenty-fifth Annual General Meeting of the British Association for Cancer Research*

### (Incorporating Symposia on "Growth Regulation of Haemopoietic Cells" and "Cellular Oncogenes" and the 1984 Walter Hubert Lecture†). April 2–4, 1984

*Held at Owens Park, Manchester University*

### Abstracts of Invited and Proffered Papers

### Symposium on "Growth Regulation of Haemopoietic Cells"

### Homeostasis in haemopoiesis

R. Schofield

*Paterson Laboratories, Christie Hospital & Holt Radium Institute, Withington, Manchester M20 6BX, UK.*

The haemopoietic system is normally maintained in dynamic equilibrium and when this equilibrium is disturbed the system responds quickly to re-establish it. This presentation will focus upon the behaviour of the haemopoietic progenitor and precursor cells responsible. I shall take erythropoiesis as the example of a cell system committed to the production of a specific end product and consider the pluripotent stem cells from which all haemopoietic cells derive. In the haemoglobinising (and therefore recognisable) erythroid cells there is flexibility in the degree of amplification they can undergo during periods of high demand. In the erythroid precursor cells which cannot be recognised morphologically (but which can be recognised functionally) there is considerable overproduction which is exploited immediately when there is increased demand. There is also an increased production of these precursor cells. The normal production and enormous reserve production capacity is maintained throughout life and would, if necessary, function for several lifespans. The supply originates within the haemopoietic tissues and depends upon a stem cell population committed solely to the production of blood cells. The stem cells are essentially "immortal" insofar as the animal's life-span is concerned. Overwhelming evidence now implicates a highly specific cellular microenvironment as being responsible for the survival and self-renewal of these cells. Microenvironmental influences also determine their rate of production and direction of differentiation.

### Proliferation control mechanisms in haemopoiesis

E.G. Wright

*Department of Anatomy and Experimental Pathology, The University of St Andrews, Fife KY16 9TS, UK.*

Mammalian haemopoietic tissue can be represented as a three-tier hierarchy of cell compartments: stem cells, committed precursor cells and maturing cells.

Stem cells in murine tissues are detected by their ability to produce macroscopic colonies of haemopoietic cells in the spleens of irradiated mice and are therefore operationally defined as colony forming units – spleen (CFU-S). An essential feature of stem cells is their capacity for "self-renewal". It is generally accepted that loss of self-renewal potential occurs concomitantly with an irreversible commitment to a more restricted potential for differentiation. Committed precursor cells (also known as progenitor cells) are detected by functional in vitro assays. They are operationally defined as colony-forming cells (CFC) that produce clones of morphologically-identifiable, maturing haemopoietic cells when appropriately stimulated.

Regulatory factors controlling the proliferative behaviour of cells at various stages in their developmental history have been demonstrated and it is becoming clear that control of cellular

*Honorary Secretary, Dr A.M. Creighton, Imperial Cancer Research Fund, P.O. Box 123, Lincoln's Inn Fields, London WC2A 3PX.

†See p. 137 of this issue.

© The Macmillan Press Ltd., 1984

proliferation in haemopoietic tissues is effected by a series of integrated inhibitor/stimulator negative feedback loops. There is increasing evidence that the distribution of proliferating and differentiating cells within the bone marrow is not random and that the various cell compartments conform to well defined spatial distributions in the marrow spaces. It is likely that the bone marrow has a spatial organisation related to the regulation of haemopoietic cell production.

**The role of stromal cells and growth factors in normal haemopoiesis and leukaemogenesis**

T.M. Dexter, A.D. Whetton & G. Bazill

*Paterson Laboratories, Christie Hospital & Holt Radium Institute, Withington, Manchester M20 9BX, UK.*

Haemopoiesis in the adult occurs mainly in the bone marrow, where the developing blood cells are found in intimate association with a variety of stromal cell types. Evidence indicates that the stroma is not simply supplying a "lodgement" area for the haemopoietic cells, but is actually having a determinative role to play in haemopoietic cell development by producing a variety of regulatory molecules essential for the survival, proliferation and differentiation of the primitive blood cells. Developing leukaemic cells, like normal cells, demonstrate an absolute dependence on the stromal cells/regulatory factors and rapidly die in their absence.

Recently, we have purified a haemopoietic cell growth factor (HCGF) which may represent a "master-control" molecule in haemopoiesis in that the growth factor a) facilitates self-renewal and differentiation of pluripotent stem cells, b) stimulates the committed myeloid progenitor cells and c) "immortalises" granulocyte precursor cells and allows their growth in the absence of stroma. HCGF. acts by modulating energy levels in the haemopoietic cells via its effect on glucose transport. The evolutionary significance of this finding and its relation with leukaemogenesis will be discussed.

**Molecular analysis of erythropoiesis**

P.R. Harrison, N. Affara, P.S. Goldfarb, K. Kasturi, E. Black, J. Fleming & J. O'Prey

*The Beatson Institute for Cancer Research, Glasgow, UK.*

Erythropoiesis involves the sequential expression of a series of characteristic red cell proteins during differentiation of committed erythroid progenitor cells, proteins such as spectrin and glycophorin, followed by the globins haem and other characteristic enzymes such as carbonic anhydrase, catalase and a specific lipoxygenase. It had been generally believed that spectrin and glycophorin, like the globins, were specific to red cells. But recent evidence by our group and others has shown that spectrin and possibly glycophorinlike molecules exist in certain other cells (Kasturi *et al.* (1983) *Exp. Cell Res.*, **144,** 241). This implies that red cell differentiation involves the tissue-specific regulation of gene families coding for spectrin and possibly glycophorin and other red cell proteins.

A major aspect of our recent work has involved cloning genes or mRNAs coding for characteristic red cell proteins (Affara *et al.* (1983) *Nucl. Acids Res.*, **11**, 931; Goldfarb *et al.* (1983) *Nucl. Acids Res.*, **11,** 3517.) with a view to elucidating the chromosomal and transcriptional changes involved in the expression of these genes during red cell differentiation. In particular, we have studied the molecular/genetic mechanisms of co-ordinate control of globin and non-globin genes by measuring their nuclease sensitivity, DNA methylation and transcription patterns in cell hybrids between erythroblasts and other cell types.

### Symposium on "Cellular Oncogenes"

*Organized jointly with the British Society for Cell Biology.*

**Specific genes involved in oncogenesis**

R.A. Weiss

*Institute of Cancer Research, Fulham Road, London SW3 6JB, UK.*

Carcinogenesis has long been recognised as a stepwise process, probably involving genetic change at several stages. The advance in our understanding of the genetics of cancer in recent years has been the identification of specific genes that play a role in malignant transformation, The transforming genes of tumour viruses have been studied most thoroughly. While the oncogenic properties of DNA tumour viruses appear to result from the expression of genes essential for viral replication, the oncogenes of retroviruses are derived from cellular genes and play no role in the life-cycle of the virus. The cellular homologues of several oncogenes have been found in changed form in human tumours. Human oncogenes have been detected by (a) tumour DNA transferred to cell lines sensitive to oncogene transformation, (b) amplified sequences in the tumour cells, and (c) chromosome translocations associated with specific types of malignancy. Some of the oncogenes detected by DNA transfer have not previously been found in viral genomes. Some oncogenes are regularly activated in experimental systems of chemical carcinogenesis; others – oncofetal genes – are normally expressed during embryogenesis and become reactivated during viral or chemical transformation. The regulation of these genes and the properties of their products will be briefly reviewed as an introduction to the subsequent talks.

### Activated ras oncogenes in human & mouse tumours

C.J. Marshall, A. Hall, R.J. Brown, Karen H. Vousden & Hugh Paterson

*Institute of Cancer Research, Fulham Road, London SW3 6JB, UK.*

We have identified the activated transforming gene in HT 1080 fibrosarcoma cells as a new member of the ras gene family. In HT1080 cells the N-ras gene is activated by an amino acid substitution of glutamine by lysine. This substitution results from a transversion, such that the mRNA contains an AAA codon instead of CAA.

In order to investigate the biological role of ras genes we have introduced the cloned genes into a variety of cell types. The transforming gene of HT1080 cells has been introduced into flat revertants of HT1080. Such transfected revertants become transformed, demonstrating that HT1080 can respond to its own activated N-ras gene. This result implies that the N-ras gene plays a role in the transformed phenotype of HT1080 tumour cells.

### Analysis of the avian erythroblastosis virus erb B oncogene products

M.J. Hayman[1], G. Kitchener[1,2] & H. Beug[2]

[1]*Imperial Cancer Research Fund, London WC2, U.K. and* [2]*European Molecular Biology Lab., Heidelberg, FRG.*

The avian erythroblastosis virus AEV, causes two diseases in young chickens, erythroblastosis and fibrosarcoma. The oncogene responsible for these diseases is known as erb, and it is capable of producing two gene products, erb B which is primarily responsible for both diseases, and erb A which plays an, as yet undefined, ancilliary role in transformation. Data will be presented on the biosynthesis of the erb B glycoprotein in both wild-type virus transformed cells and cells transformed by temperature-sensitive mutant virus. The results of these experiments show that surface expression of erb B correlates with transformation, and suggests that erb B may be functioning as a growth factor receptor-like molecule to induce the uncontrolled proliferation of the fibroblasts and erythroid cells it transforms.

### Papillomaviruses

W.F.H. Jarrett

*Department of Veterinary Pathology, University of Glasgow Veterinary School, Bearsden, Glasgow G61 1QH, UK.*

Papillomaviruses are not known to interact with any cellular oncogene. Bovine papillomaviruses 1 and 2 transform primary fibroblasts in tissue culture and also rodent cells. Using this system in transfection assays it has been shown that a fragment comprising 69% of the viral genome is required for transformation. This fragment contains two genes which give rise to five transcripts. Current work on the structure and action will be presented.

### Activation of cellular genes in transformed cells

P.W.J. Rigby, P.M. Brickell, D.S. Latchman, D. Murphy, M.R.D. Scott, K-H. Westphal & K. Willison

*Cancer Research Campaign Group*
*Department of Biochemistry, Imperial College and Chester Beatty Laboratories, Institute of Cancer Research, London, UK.*

We have used molecular hybridisation and cDNA cloning techniques to isolate a number of cellular genes which are transcriptionally activated in a variety of murine transformed and tumour cell lines. These cDNA clones have been used both to characterise the corresponding genomic sequences and to analyse gene expression in tumour cells. The cDNA clones of Set 1 define a Major Histocompatibility Complex gene from the Qa/Tla region, the activation of which is a general feature of oncogenesis in the mouse. The inappropriate expression of this embryonic MHC antigen has a number of interesting immunological implications. cDNA clones of Set 2 define an endogenous C-type retrovirus which is activated in a number of transformed cell types. cDNA clones from several other Sets derive from inappropriate processing of transcripts from the mitochondrial genome. This experimental approach has allowed us to define a number of ways in which the cell responds to the expression of an oncogene.

### Oncogenes of adenoviruses

P.H. Gallimore

*Department of Cancer Studies, University of Birmingham, Medical School, Birmingham, B15 2TJ, U.K.*

Human adenoviruses can be divided into oncogenic and non-oncogenic serotypes. Most adenoviruses transform rodent cells and the gene regions participating in transformation have been determined. Recent studies have focused on Early Region 1 (El) which contains two transcription units Ela and Elb. The development of Ela and Elb mutants and intratypic recombinant plasmids with DNA transfection studies indicate that the interaction of adenovirus genes in transformation and tumour induction is complex. Expression of Ela alone only produces rare "atypical transformants". Mutations in Ela or Elb can abolish the transforming and tumourigenic phenotype. Our knowledge of El proteins will be discussed in the light of our current understanding of the properties of other oncogenes.

### Oncogenes may encode a growth factor or part of the receptor for a growth factor

M.D. Waterfield

*Protein Chemistry Laboratory, Imperial Cancer Research Fund, Lincoln's Inn Fields, London WC2A 3PX, U.K.*

Studies of the structure of platelet derived growth factor (PDGF) have shown that simian sarcoma virus has acquired cellular sequences which encode a protein almost identical to the precursor of one polypeptide chain of PDGF (Waterfield *et al.*, *Nature* **304,** 35, 1983; Johnsson *et al.*, to be published). This polypeptide is a growth factor (Deuel *et al.*, 1983, *Science*, **221,** 1348) and related mRNA transcripts and polypeptides have been detected in human tumour cell lines which would be expected to respond to a PDGF-like molecule (glial and connective tissue – Eva *et al.*, 1982, *Nature*, **295,** 116; Heldin *et al.*, 1980, *J. Cell. Physiol.*, **105,** 235). This suggests that "Autocrine" growth factor production would play a role in abnormal growth control of transformed or tumour derived cell lines.

Recent studies of the amino acid sequence of immunoaffinity purified EGF receptors have shown that the v-erb-B oncogene may encode a truncated receptor lacking the external ligand binding domain (Downward *et al.*, 1984, *Nature*, in press). This result suggests that an alternative to autocrine growth factor expression is subversion of a receptor function. Since the v-erb-B oncogene is related to the src subset of retroviral oncogenes other putative transforming proteins may be related to other growth factor receptors. The implications of these two observations will be discussed.

### Abstracts of members' proferred papers

### Phase I clinical trial of CB3717 (N-(4-(N-(2-amino-4-hydroxy-6-quinazolinyl)methyl)prop-2-ynylamino)benzoyl-L-glutamic acid)

D.L. Alison, B. Robinson, S.J. Harland, B.D. Evans & A.H. Calvert

*Royal Marsden Hospital, Sutton and London, UK.*

The quinazoline folate analogue CB3717 is a potent thymidylate synthetase inhibitor. A phase I clinical trial was carried out at the Royal Marsden Hospital recruiting 101 patients. The schedule of administration was a one hour infusion of the drug dissolved in 250 ml of 0.15 $NaHCO_3$ at pH 9.0 repeated every 3 weeks. Doses were escalated from 140 mg m$^{-2}$ to 600 mg m$^{-2}$ which was the maximum tolerated dose. The dose limiting toxicity was renal, and predicted by preclinical studies with significant reduction in $^{51}Cr$ EDTA clearances (>50%) occurring with increasing frequency at doses of 500–600 mg m$^{-2}$. Below 500 mg m$^{-2}$ reduction of 20–50% pretreatment GFR occurred occasionally. Reversible hepatic toxicity typified by a rise in alanine transaminase levels occurred frequently (80% patients with increase to 1–2 times normal, 50% patients with increase >2 times normal) and was associated with malaise of several days duration. Alkaline phosphatase levels also rose but to a lesser extent. Prolongation of the infusion time to 12 h did not affect either the incidence or severity of this toxicity which was seen at all dose levels. Administration of prednisolone 30 mg daily for 1/52 after treatment ameliorated the malaise but not the biochemical lesion. In 17 patients the WBC was suppressed (nadir day 10, recovery days 14–16) and in 6 thrombocytopenia developed. Ten patients developed rashes. Neither the myelotoxicity nor the skin toxicity was dose related. The following responses were observed in evaluable patients at doses between 200–600 mg m$^{-2}$: ovary 1 CR, 1 PR, 7 MR/30; breast 3 PR, 1 MR/8; lung – adenoca 1 PR, 1 MR/8; bowel 2 MR/5; mesothelioma 1 PR/5. We conclude that CB3717 deserves Phase II evaluation for which we recommend the relatively non-toxic dose of 400 mg m$^{-2}$.

**Results of a clinical phase I study of 1,2,4, triglycidylurazole (TGU NSC 332488) with pharmacokinetic profile**

M. Soukop, F. Stuart, D. Cunningham, N. Gilchrist, G. Forrest & A. Setonoian

*Department of Medical Oncology, Glasgow Royal Infirmary and Department of Clinical Oncology, Glasgow University, Scotland, UK.*

Thirty one patients with advanced malignancies received TGU in a phase I study. Therapy was given initially by push injection in physiological saline at 3 weekly intervals. Dose escalation from 30 mg m$^{-2}$ to 900 mg m$^{-2}$ was used with a minimum of 3 patients at each dose level. Myelosuppression was dose-limiting and when it occurred resulted in a white count nadir at 16.5 ± 7.5 days with recovery at 34.5 ± 17.6 days. Platelet nadir 15.4 ± 6.3 days (recovery 36.8 ± 21.7 days). Side effects in patients treated with 800 or 900 mg m$^{-2}$ were as follows: nausea and vomiting (WHO grade) 0 in 1 course, 2 in 9 and 3 in 12 courses. Alopecia minimal in 1 patient. When TGU was given by infusion for pharmacokinetic reasons 2 cases of severe thrombophlebitis were observed. This severity of local toxicity was not seen with bolus administration. No renal, hepatic or cardiac toxicity were seen. Two minor clinical responses and one partial response (PR) were seen. The PR occurred in a patient with an unknown primary (adenocarcinoma) resistant to prior therapy with 5FU, Adriamycin and Mitomycin-C.

An HPLC analytical method has been developed with a sensitivity of 10 $\mu$g ml$^{-1}$' The data obtained fit a 2 compartment pharmacokinetic model with a biexponential decay of very short $T\frac{1}{2}\alpha$ of 3.8 min and $T\frac{1}{2}\beta$ of 8.6 min. In conclusion TGU appears to be a new triepoxide worthy of Phase II study using a dose of 800 mg m$^{-2}$ given q 3 or 4 weeks and with acceptable, predictable toxicity.

**Phase I clinical pharmacokinetics of CCRG 81010 (M & B 39565, NSC 353451)**

C. Goddard, J.A. Slack, [1]E.S. Newlands, [2]G. Blackledge, [1]C.J. Brindley & M.F.G. Stevens

*Cancer Research Campaign, Experimental Chemotherapy Group, Department of Pharmacy, University of Aston in Birmingham, [1]Charing Cross Hospital, London. [2]Queen Elizabeth Hospital, Birmingham*

CCRG 81010 (8-Carbamoyl-3-(2-chloroethyl)-imidazo (5,1-d)-1,2,3,5-tetrazin-4(3H)-one) is a novel agent active against a wide range of murine tumours and is currently undergoing Phase I clinical evaluation. The drug was formulated as a 50 mg ml$^{-1}$ solution in dimethyl sulphoxide and diluted with 500 ml of isotonic saline prior to administration as a 1 h i.v. infusion. The trial began in June 1983 at a dose of 8.0 mg m$^{-2}$. Plasma samples were collected over 24 h and analysed for parent drug by HPLC (Slack *et al.*, 1983, *PAACR*, **24**, 1152). The pharmacokinetic data currently available is summarised below, with the data expressed as means for each dose level:

| | | | | |
|---|---|---|---|---|
| Dose (mg m$^{-2}$) | 8.0 | 16.5 | 33.0 | 54.4 |
| Number of doses | 6 | 9 | 5 | 4 |
| $t\frac{1}{2}$(h) | 1.2 | 1.1 | 1.3 | 1.0 |
| AUC (mg h l$^{-1}$) | 0.855 | 1.410 | 2.510 | 4.729 |
| Volume of Distribution (l) | 29.0 | 34.7 | 47.2 | 31.3 |
| Dose (mg m$^{-2}$) | 82.0 | 115.0 | 153.0 | |
| Number of doses | 4 | 5 | 3 | |
| $t\frac{1}{2}$(h) | 1.2 | 1.2 | 1.0 | |
| AUC (mg h l$^{-1}$) | 6.297 | 9.045 | 12.311 | |
| Volume of Distribution (l) | 40.4 | 43.7 | 38.5 | |

The data appears to be in agreement with a single compartment open model and the drugs elimination from plasma (with $t\frac{1}{2}$ just in excess of 1 h) correlates well with mouse studies. The maximum tolerated dose is expected to be attained within 2 further dose escalations. The bioavailability of oral capsules and the excretion of $^{14}$C-labelled CCRG 81010 is currently under investigation.

### Human 2 interferon (IFN) (Schering Plough 30500) in the non-Hodgkin lymphomas (NHL): A report of 2 phase II studies

J. Wagstaff & D. Crowther

*CRC Department of Medical Oncology, Christie Hospital, Manchester, UK.*

Eighteen patients with centrally reviewed histologically confirmed CS III and IV low grade NHL (DWDL = 8, NPDL = 5, NM = 3) were treated with $2 \times 10^6$ IU $m^{-2}$ IFN by sc injection 3 times per week for 3 months. Five were previously untreated. Patients with PR or static disease were continued until progression or 1 year. The IFN was well tolerated with mild subjective toxicity (myalgia, malaise and tiredness), myelosuppression was mild and there was no hepative toxicity. Six patients are still on treatment. The median duration of treatment was 3 months (0.25–12). One patient achieved CR after 4 months therapy. She stopped IFN after 1 year and remains disease free 2 months later. Three others have achieved PRs (6 weeks, 3 months and 10 months+). Four are static (1 stopped, 3 continuing). This IFN has activity in approximately 25% of these patients.

Seven patients with refractory high grade NHL(DPDL = 6, lymphoblastic = 1) were treated with $250 \times 10^6$ IU $m^{-2}$ IFN as a 24 h infusion at 3–4 weekly intervals. Median number cycles = 3 (2–5). Acute toxicity was severe (malaise, high fever, rigors, nausea and vomiting). More chronic toxicity (malaise, anorexia and lethargy) took 4–7 days to resolve. Myelosuppression and hepatotoxicity were rapid, mild and reversible. Four patients had rapid disease regression which began to recur before the next cycle. Only 2 of these qualified for PR. This suggests that very high dose IFN is active in this disease but that the more continuous scheduling which would be required to prevent mid-cycle progression may be intolerable.

### Platinum (CP)-induced emesis. Evaluation of dexamethasone (D) in combination with high dose metoclopramide (HDM)

S.G. Allan, M.A. Cornbleet, M.W. Ramsay, P.S. Warrington, R.C.F. Leonard & J.F. Smyth

*Department of Clinical Oncology, Western General Hospital, Edinburgh, UK.*

In a randomised, double-blind study of 34 patients undergoing treatment with CP for a variety of malignancies, 102 treatment courses were given using D + HDM or placebo (P) + HDM in random sequences as the anti-emetic. The age range was 30–67, mean 56 years. CP at 30 mg $m^{-2}$ was given on 60 courses and 100 mg $m^{-2}$ on 42 courses. HDM (5 mg $kg^{-1}$) made up in 500 ml of normal saline was infused in 100 ml aliquots 1/2 h before CP (1 h infusion), and then 2 hourly thereafter. D (20 mg) or P (normal saline) was given by i.v. bolus prior to the first HDM dose. 16–18 h after CP, a questionnaire was completed to record the number of dry retches (DR), vomits (V), meals eaten, bouts of diarrhoea and side-effects related to anti-emetic therapy. Nausea (N) was evaluated using a visual analogue scale. Duration of nausea and vomiting was recorded. The results are recorded as % of courses under the following coding: A = free of all N, DR + V; B = free of N; C = free of DR; D = free of V; E = ⩽1 day duration N; F = ⩽1 day duration V; G = diarrhoea; H = drowsiness.

| | *A* | *B* | *C* | *D* | *E* | *F* | *G* | *H* |
|---|---|---|---|---|---|---|---|---|
| D + HDM | 23 | 33 | 50 | 35 | 34 | 50 | 6 | 46 |
| P + HDM | 10 | 24 | 34 | 26 | 13 | 33 | 18 | 36 |

50% of patients in both groups ate all meals offered during chemotherapy and an equal number (35%) in both groups experienced tremulousness. We conclude that D enhances the anti-emetic efficacy of HDM and promotes more rapid recovery from N + V induced by CP. D appears to diminish the CP-induced diarrhoea and is not associated with additional side-effects.

### Lymphocyte migration in patients with non-Hodgkin's lymphoma (NHL) and Hodgkin's disease (HD): A novel form of therapy

J. Wagstaff, M. Birch, H. Sharma, W. Ford & D. Crowther

*Department of Medical Oncology, Christie Hospital, Departments of Cellular Immunology and Medical Physics, University of Manchester, UK.*

The lymphocyte is a nomadic cell and those seen within the blood are destined soon to leave it, to traverse the tissues and thence to return to the blood via the thoracic duct. Malignant lymphocytes possess unique patterns of migration. For example in patients with gastro-intestinal tract NHL the disease frequently remains localised to the abdomen despite there being lymphoma cells in the blood.

We have studied the patterns of migration of autologous lymphocytes labelled with indium-III oxine in normal subjects (Wagstaff *et al.* (1981). *Clin. Exp. Immunol.*, **43,** 435; Wagstaff *et al.*, 1981, *Clin. Exp. Immunol.*, **43,** 443). We present here the results of studies in patients with HD and NHL. Normal lymphocytes accumulate preferentially at sites of disease in both NHL and HD. In HD this phenomenon is sufficiently large to account for the peripheral blood lymphopenia and impaired reaction to cutaneously administered recall antigens frequently seen in this disease. More recently experiments in rats using lymphocytes labelled with the $\beta$-emitter $^{114}$In show that therapeutic doses of radiation may be delivered to the lymphoid tissues without undue toxicity. Similar experiments are being conducted in man and preliminary data will be presented. This approach promises to be a novel therapeutic manoeuvre for selectively irradiating sites of disease in both NHL and HD.

### Heterogeneity of human natural killer (NK) cells at the clonal level

K. Roberts & M. Moore

*Department of Immunology, Paterson Laboratories, Christie Hospital & Holt Radium Institute, Manchester M20 9BX, UK.*

In human peripheral blood, most natural killer (NK) cells are found in the null cell fraction, morphologically identifiable as large granular lymphocytes (LGL). However, analysis of purified LGL with monoclonal antibodies to defined cell surface determinants has shown them to comprise a heterogeneous population. To determine whether NK recognition structures are clonally distributed we have recently developed human NK clones by limiting dilution (LD) and expansion in interleukin-2. While all of these have retained their characteristic morphology, there is significant inter- and intra-donor variation in the proportion of cytotoxic clones developed and the specificity of their reactions e.g. 14/32 clones from one LD were cytotoxic of which 4 killed both K562 and Molt 4 targets, while 10 killed K562 only; another LD yielded 3/9 cytotoxic clones of which 2 killed K562 but not Molt 4 and 1 killed both tragets. The heterogeneity has been confirmed by comparative cytotoxicity tests on a wider range of targets and cumulatively the data indicate that target cell recognition structures are clonally distributed. Some dissociation between NK and K cell activities (the latter assayed against antibody – sensitised A Rh(D)+ve erythrocytes) – normally attributed to be different functions of the same cell type – has also been observed.

### Influence of phorbol acetate (TPA) on the susceptibility of K562 to natural cytotoxicity: Evidence for clonal variation in differentiation-induced changes of lytic sensitivity

I. Kimber[1] & M. Moore

[1]*Immunology Section, ICI, Central Toxicology Laboratory, Alderley Park, Macclesfield, Cheshire SK10 4TJ; Department of Immunology, Paterson Laboratories, Christie Hospital & Holt Radium Institute, Manchester M20 9BX, UK.*

The effect of 12-0-tetradecanoylphorbol-13-acetate (TPA) on the sensitivity to NK cell-mediated lysis of 2 cloned populations of K562 which exhibit marked and stable differences in their susceptibility to natural cytotoxicity has been examined. Culture in medium supplemented with concentrations of TPA of $1\,ng\,ml^{-1}$ or greater invariably caused a decrease in the susceptibility of the sensitive clone E10/P2 whereas treatment of the relatively resistant clone F9/P2 with TPA under identical conditions caused a significant increase in susceptibility to natural cytotoxicity. In both cases the change in susceptibility occurred within 1 day of culture in TPA and was rapidly reversible following removal of the inducing agent. The changes in resistance to natural cytotoxicity induced by TPA were independent of variations in osmotic fragility and were not attributable to alterations in NK cell binding capacity as determined by cold competition analysis. In contrast to the effect of TPA, exposure of E10/P2 and F9/P2 to interferon (IFN) caused a reduction in sensitivity to natural cytotoxicity of both populations which was associated with a decreased capacity to compete for lysis of labelled target cells. These data suggest that the effects of differentiating agents on target susceptibility to NK cell lysis are variable and clonally distributed within cell populations.

### Inhibition of human natural killer (NK) activity by alkylating ketones: Evidence for the involvement of cell surface proteases in the lytic event

M.M. Dawson, U. Shipton & M. Moore

*Department of Immunology, Paterson Laboratories, Christie Hospital & Holt Radium Institute, Manchester M20 9BX, UK.*

The lysis of target cells by Natural Killer (NK) cells is a complex process that is probably mediated by proteins in the effector cell plasma membrane. Proteases have been implicated in the NK-mediated lysis of susceptible target cells since a variety of molecules with anti-protease activity also inhibit NK cytotoxicity in vitro (Hudig, *et al.*, 1981, *J. Immunol.*, **126,** 1569). K562 lysis by human NK cells was used to study the effects of the small molecular weight antiproteases tosyl-L-lysyl chloromethyl ketone (TLCK) and tosyl-L-phenylalanyl chloromethyl ketone (TPCK) which are know to inhibit serine proteases by alkylation of a histidine residue at the active site of the enzyme (Shaw, *et al.*, 1965, *Biochemistry*, **4,** 2219). Our studies indicate that the effects of TPCK and TLCK are immediate and are manifested at the effector level: Pretreatment of effectors for 30 min with either inhibitor was sufficient to inhibit lysis of K562 whereas pretreatment of target cells was ineffective. The effects of TLCK and TPCK were irreversible and non-toxic: Lymphocytes pretreated in this manner did not recover NK activity after 2 days in culture although they were still viable by dye exclusion. Kinetic data derived from experiments in which TLCK was added at different time intervals after effector: target cell interaction suggest that post-binding events are also affected by this agent. The contribution of intracellularly-bound TLCK to the inhibitory process is currently being investigated by protection experiments using lectins as reversible ligands of the NK cell surface.

### The growth of human AML cells as solid tumours in immune-deprived mice

R.D. Clutterbuck, C. Hills, P. Hoey, V. Shepherd, R. Powles, P. Alexander[1] & J.L. Millar

*Institute of Cancer Research, Sutton, Surrey, [1]CRC Medical Oncology Unit, Southampton General Hospital, Hants, UK.*

Human AML cells from the blood of a series of patients have been stored in liquid $N_2$ and later thawed and implanted subcutaneously into mice immune-suppressed by thymectomy and total-body irradiation. Solid tumours resulted from the majority of these inoculi which grew to a maximum size, dependent upon patient and size of inoculum, and then spontaneously regressed. A second inoculum of AML cells into animals with regressing tumours also produced tumours and thus regression cannot be accounted for on the basis of returning immunity and may be related to maturation in vivo similar to that seen in vitro. Treatment with human GM-CSF derived from human placental conditioned medium, or medium conditioned by 5637 bladder carcinoma cells, failed to influence either the growth or disappearance of these tumours. Preliminary results show that mononuclear cells derived from normal human bone marrow behave in a similar manner to AML cells by producing local growth followed by regression.

### The effect of cell-to-cell interactions on heat sensitivity of HT29 human tumour cells

J. Dobrucki & N.M. Bleehen

*University Department and MRC Unit of Clinical Oncology and Radiotherapeutics, Cambridge CB2 2QH, UK.*

The relation between cell-to-cell interactions and cellular heat sensitivity has been investigated using the HT29 human colonic adenocarcinoma cell line. A comparison has been made between cells growing under different culture conditions, as single cells or as cells growing in close cell-to-cell contact. The population of separated cells was obtained by using the chelating agent EDTA. Cells dispersed with EDTA resume logarithmic growth on a substratum without any lag period. EDTA treatment was found to have no effect on heat sensitivity. Cells growing as small multicellular spheriods (5–8 cells, diameter 20–35 $\mu$m) floating in the medium constituted the second population in the model system. The damage caused by heat treatment was measured using two different end-points, ability to form colonies and growth delay of the heated cells. The latter permits a direct comparison of the damage which occurs within both compared cell populations. It also avoids the need to disaggregate the cells with dispersing agent after heat treatment.

The two cell populations exhibit differences in their heat sensitivity to heat treatment at 43°C, for up to 4 h. The survival curves of both populations differ in the width of the shoulder, but not in the slope. Heating spheroids for 30 min does not effect the survival. The same treatment reduces the surviving fraction of single cells to the level of 85%. The growth delay is longer for single cells than for spheroids by a factor of 1.4. This difference is constant for heating times between 1 and 4 h at 43°C.

### Sister chromatid exchange frequency in the lymphocytes of patients having therapy for Hodgkin's disease

T. Brown, A.A. Dawson & J.L. Watt

*Departments of Medical Genetics and Medicine, University of Aberdeen, UK.*

The method of harlequin staining at 2nd cell division *in vitro*, was used to demonstrate sister chromatid exchanges (SCE) in peripheral lymphocytes of patients undergoing firstline treatment for Hodgkin's disease.

The method provides a highly sensitive indicator of induced genetic alteration at the DNA level and various cytotoxic drugs, especially the alkylating agents, have been shown previously to increase SCE frequency. The pattern of elevation has not previously been followed throughout and after a course of standard chemotherapy, such as is used in Hodgkin's disease. SCE frequency was followed in 20 patients with newly-diagnosed Hodgkin's disease, 11 having MVPP chemotherapy and 9 mantle radiotherapy. A characteristic pattern of response to chemotherapy was found, with a dramatic rise until about the 20th week of therapy, with a fall before the end of the course. With radiotherapy there is a small but sustained reduction in SCE frequency. Thirty-two Hodgkin's disease patients, who had had MVPP 2–12 years previously, were also studied, and their SCE frequency had reverted to normal.

The findings may have considerable significance with regard to short-term response and long-term prognosis.

### High dose melphalan (HDM) in multiple myeloma

P. Selby, R. Powles, B. Robinson, M. Vincent & T.J. McElwain

*Royal Marsden Hospital, Downs Road, Sutton, Surrey, UK.*

Eighteen patients aged <60 years with multiple myeloma have been treated with melphalan 140 mg m$^{-2}$I.V. (McElwain & Powles 1983, *Lancet*, 1983, ii 822). All developed severe leucopenia and thrombocytopenia and needed intensive support. Among previously untreated patients 2/9 are too early to assess, 6/9 recovered well taking a median 28 days (25–33) to restore leucocyte counts to $10^9 l^{-1}$. All had moderate nausea and vomiting for 24–48 h, mild diarrhoea and alopecia. One patient died 16 days after HDM of intractable pulmonary oedema probably because a rapidly falling paraprotein reduced his oncotic pressure. Among the 6 assessable patients 3/6 cleared their bone marrows of myeloma and 3/5 who secreted paraproteins remitted completely biochemically. One patient who had entered a stable partial remission on treatment with cyclophosphamide, completely remitted with HDM. Eight patients had relapsed or failed to respond to extensive previous chemotherapy including oral melphalan. Median time to $10^9 l^{-1}$ leucocytes was 48 days (23–56) in 5 patients who had numerous infections. One patient is too early to assess but two died, (cerebral haemorrhage day 20; chest infection day 45). One patient had a complete biochemical and marrow remission for 5 months but subsequently relapsed and died. In 4 others the paraprotein levels fell but one rapidly relapsed and died, one died in remission of a fungal infection, and two are alive in partial remission. HDM is promising for untreated myeloma patients under 60 years. Intensive supportive care is required. Patients who are resistant to low dose treatment may respond, but toxicity is more severe and they may benefit less.

### High dose ifosfamide and mesna after adriamycin VP16213 and vincristine (VP-AV) for small cell lung cancer

B.M.J. Cantwell, A.L. Harris, J. Bozzino, P. Corris, S. Nariman, S. Pearce, J. Gibson, D. Hendrick, A. Lishman, A. Hendrick & R. Leonard

*Department of Clinical Oncology, Newcastle General Hospital, Newcastle upon Tyne, NE4 6BE, UK.*

The role of high dose ifosfamide in small cell lung cancer (SCLC) has been evaluated in a crossover study. 39 patients with SCLC have been treated with adriamycin 40 mg m$^{-2}$ i.v., vincristine 2 mg i.v., VP16213 100 mg m$^{-2}$ i.v., all on day 1 and VP16213 200 mg m$^{-2}$ orally days 2 and 3 every 3 weeks for 3 courses. Patients with limited disease who had a partial or complete response had radiotherapy to the mediastinum and primary lesion (24 Gy, 4 fractions, 8 days) starting 3 days after course 3. Consolidation therapy with 2 courses of ifosfamide 5 g m$^{-2}$ and mesna 5 g m$^{-2}$ by 24 h infusion was given 3-weekly $\times$ 2. All treatment was finished by week 12 from start of therapy. Patients with extensive disease with response to VP-AV crossed over to high dose ifosfamide for 3 courses. Patients who did not respond to VP-AV after 2 courses crossed over to 3 courses of high dose ifosfamide, 3-weekly. Overall responses to treatment regimen: 13/22 patients with limited disease had CR, 7/22 PR (91% response rate); 17 patients had extensive disease, 2 had CR, 9 PR (65% response). On crossover to ifosfamide 4/7 patients with limited disease not showing PR or CR to VP-AV, showed subsequent response. 3/7 patients with extensive disease and PR to VP-AV had further response to ifosfamide (50% 2nd line response rate). There were 4 episodes of septicaemia on VP-AV, (3 associated with radiotherapy). In 61 courses of ifosfamide and mesna only 1 episode of neutropenia occurred. High dose ifosfamide is an effective, relatively marrow-sparing agent in SCLC and should be assessed as first line treatment. Short courses of chemotherapy can be integrated with radiotherapy and produce a high response rate with a low incidence of serious toxicity.

**Phase II study of cis-1,1-diamino-methylcyclohexane sulphate platinum (TNO-6) in gastric and colo-rectal adenocarcinoma**

D. Cunningham, M. Soukop, N.L. Gilchrist, G.J. Forrest, C.S. McArdle, D.C. Carter, J.W. Dobbie, M. Smith, S.B. Kaye & K.C. Calman

*Royal Infirmary and Gartnavel General Hospital, Glasgow, UK.*

Cis-platinum has activity in gastric cancer. We have evaluated the activity of its analogue TNO-6 in gastric and colorectal adenocarcinoma. Twenty two patients entered the study. All had histologically proven disease. Sixteen patients had gastric cancer, six of whom had received previous chemotherapy with F.A.M., and six colorectal cancer three of whom had received previous 5-fluorouracil. All patients had measurable disease. TNO-6 30 mg m$^{-2}$ every 4 weeks was administered as an i.v. bolus in 16 patients, but was combined with hyperhydration in the final six patients entering study as nephrotoxicity was reported by other workers. No responses were seen in colorectal cancer. In the gastric cancer group, one patient had a partial response, two had stable disease and 13 progressive disease. Moderate nausea and vomiting occurred in 12 patients and alopecia in one. Creatinine clearance was unaffected by chemotherapy, though in six patients in whom the renal tubular enzyme N-acetylglucoseaminidase was measured all showed significant, transitory elevation. Myelosuppression was not marked – leukopenia grade 2 in three patients: grade 3 in one; thrombocytopenia grade 3 in one. Hepatotoxicity was not observed.

TNO-6 has minimal activity in gastric cancer. The unpredictable nephrotoxicity of this agent reported by other workers has led to its withdrawal by the manufacturers, Bristol Myers, from further phase II studies.

**Cis-dichlorodiammineplatinum (cisplatinum) and etoposide (VP16):] [An effective salvage regimen in malignant lymphoma**

I.R. Judson & E. Wiltshaw

*Department of Biochemical Pharmacology, Institute of Cancer Research, Sutton, Surrey and Royal Marsden Hospital, London, UK.*

Twenty five patients with non-Hodgkin's lymphoma (NHL) unresponsive to standard combination chemotherapy were treated with cisplatinum 50 mg m$^{-2}$ i.v. × 1, plus VP 16 100 mg m$^{-2}$ i.v. daily × 3 q. 3 wks. An average of 3 courses were given. All patients were heavily pre-treated: 66% had received prior radiotherapy plus chemotherapy, 28% three or more different drug regimens. Seventeen patients were evaluable for response. There were 5 complete remissions (CR) 29%, and 4 partial remissions (PR) 24%, giving an overall response rate of 53%. The median response duration for CR was 12 wks. Median survival for patients in CR was 25 wks compared with only 5 wks for non-responders. Toxicity included nausea and vomiting, alopecia, minor renal impairment and myelosuppression. This was occasionally severe; wbc $<$ 1,000 mm$^{-3}$ 3 patients (18%), platelets $<$ 50,000 5 patients (29%). There was one treatment-related death in a patient with bone marrow infiltration. The response rate for cisplatinum plus VP 16 is superior to that reported for either single agent (PR only 26% and 30% resp.) and the role of this combination in the primary treatment of poor prognosis NHL is being explored.

### Weekly alternating chemotherapy followed by radiotherapy for small cell carcinoma of the bronchus (SCCB)

S.M. Crawford[1], D. Parker[3], M.G. Glaser[2], B.R. Southcott[2] & E.S. Newlands[1]

[1]*Departments of Medical Oncology and* [2]*Radiotherapy, Charing Cross Hospital, London W6 8RF and* [3]*Bradford Royal Infimary, West Yorkshire BD9 6RJ, UK.*

Whilst high response rates to chemotherapy are readily obtained in SCCB, the survival of these patients remains poor. We have employed a schedule of chemotherapy of short duration followed by radiotherapy to sites of bulk disease. The aims of this approach were to assess the effectiveness of this schedule and to maximise the time the patient enjoys off treatment. The schedule is:

Course A: Etoposide $200\,mg\,m^{-2}$
Methotrexate $50\,mg\,m^{-2}$
Vincristine $1.4\,mg\,m^{-2}$ (max. 2 mg)

Course B: Adriamycin $20\,mg\,m^{-2}$
Cyclophosphamide $400\,mg\,m^{-2}$

Course A and B alternating weekly for eight weeks. 5000 cGy were then given to sites of bulk disease as appropriate.

As at 1st December 1983, twenty four patients have been entered. Toxicity has been very acceptable to the majority of patients. Median survival in the unstratified group on 1st December 1983, was forty weeks.

Pending the advent of treatment able radically to improve the outlook in this disease, this strategy of short duration treatment seems to offer the patient a greater prospect of survival off treatment.

### The prognostic significance of laboratory parameters and performance status at diagnosis for survival in small cell lung cancer

R.L. Souhami, I. Bradbury & S.G. Spiro

*University College Hospital, Gower Street. London WC1, UK.*

Previous studies have shown that performance status (PS) and disease extent (limited or extensive disease) are the most useful prognostic parameters in small cell lung cancer (SCLC). We have assessed the prognostic impact of these and other measurements made at diagnosis in 370 patients with SCLC entered into a randomised trial assessing the effect of radiotherapy (RT) to the primary tumour and mediastinum in addition to 12 courses of cyclical chemotherapy. The effect of the addition of RT to survival was negligible. The following parameters were individually significantly related to survival at $P<0.01$: Disease extent, PS, albumin (Alb; $\leqslant 39$ *vs* $\geqslant 40\,g\,l^{-1}$), sodium (Na; $\leqslant 135$ *vs* $\geqslant 135\,\mu mol\,l^{-1}$), alkaline phosphatase (AP; $\leqslant 1.5\times$ upper normal limit $\geqslant$), $\gamma$ glutamyl transferase ($\leqslant 0.$–$28$ *vs* $\geqslant 29\,IU^{-1}$). Two step-wise multiple regression analyses both showed that only PS, Alb, Na, AP and disease extent each added significantly to prognosis. The best prognostic group (63 patients) with Alb $\geqslant 40\,g\,l^{-1}$, Na $\geqslant 136\,\mu mol\,l^{-1}$, Ap $\leqslant 1.5\times$ upper normal limits and PS $\geqslant 7$ Karnofsky comprised 35 patients with limited disease and 25 extensive disease had a median survival in excess of that of the 127 limited disease patients in the entire study. It appears possible to select different prognostic groups at diagnosis on the basis of simple laboratory parameters (Alb, Na, AP) and PS. These variables offer as good a stratification of disease prognosis as disease extent, which usually includes more numerous and invasive tests.

### Extracellular ATP induces ion fluxes and inhibits cell growth of murine erythroleukemia cells

S.B. Chahwala & L.C. Cantley

*CRC Experimental Chemotherapy Group, Department of Pharmacy, University of Aston, Birmingham B4 7ET U.K. and Department of Biochemistry and Molecular Biology, Harvard University, Massachusetts, USA.*

Changes in ion flux across the plasma membrane play a pivotal role in the regulation of proliferation and differentiation. Exogenous ATP has been shown to cause a profound increase in the permeability of the plasma membrane to certain ions and to selectively inhibit the growth of transformed cells. The mechanism of these effects is not known. We have attempted to characterise more fully the nature of the ATP-induced changes in ion flux using Friend virus-infected murine erythroleukemia cells (MEL), and to study the effect of ATP on their growth and differentiation. ATP (1 mM) inhibited MEL cell growth with an increase in doubling time but did not bring about differentiation as measured by benzidine staining. It also had no effect on DMSO-induced differentiation of MEL cells. ATP (1 mM) coincubated with $^{45}Ca^{2+}$ stimulated the initial rate of $Ca^{2+}$ exchange from a basal level of 5 pmoles $min^{-1}$ to 18 pmoles $min^{-1} 10^{-6}$ cells to achieve a twofold increase in steady-state ($Ca^{2+}$) as measured by isotopic equilibration. $Ca^{2+}$ influx was blocked by DIDS, an inhibitor of anion transport. $Ca^{2+}$ was cotransported with $Cl^-$ anion resulting in an electroneutral flux, the ratio of ATP stimulated $Cl^-$ to $Ca^{2+}$ uptake being 1.6:1. $K^+$ and $Na^+$ influx were also stimulated, the $Na^+$ influx dissipating the $Na^+$ gradient and thus inhibiting nutrient uptake. $Na^+$ influx and $Ca^{2+}$ influx occurred by separate independent routes, since $Na^+$ influx was not inhibited by DIDS. The effects observed were specific for ATP (Km MgATP = 0.93 mM), since AMP, GTP, adenosine and slowly hydrolysable ATP analogue AMP-PNP were without effect. These ATP-induced changes in ion flux are considered to be responsible for growth inhibition and may form the basis for therapeutic intervention.

### An investigation of the relationship between tumour geometry and cytotoxic drug response using an *in vitro* tumour model

T.T. Kwok & P. Twentyman

*MRC Clinical Oncology and Radiotherapeutics Unit, Hills Road, Cambridge CB2 2QH, UK.*

We are using multicellular tumour spheroids to investigate the relationship between factors determined by tumour geometry and the response of cells within solid tumours to cytoxic drugs. In addition to the question of drug diffusion, it is known that gradients of pH, oxygenation and proliferative status exist within spheroids and these appear likely to influence drug response. Spheroids of the EMT6/VJAC mouse tumour cell line have been grown to a diameter of 800 $\mu$ and then bacterial neutral protease has been used to successively "strip" 4 sub-populations of cells from the surface. These sub-populations have been characterized in terms of $^3$HTdR labelling index, cell cycle distribution, RNA contents, cell size and clonogenic capacity. Cells from the outer layers were larger and proliferating more rapidly than cells deeper within the spheroids. The plating efficiencies of cells from all sub-populations were similar. When spheroids were treated with adriamycin or with nitrogen mustard (HN2) for 1 h before disaggregation, killing of clonogenic cells was greatest in the outer layer with reduced killing towards the centre of the spheroid. For CCNU, however, the reverse was true with greatest cell killing occuring deeper in the spheroid. Selective disaggregation of spheroids before drug treatment showed only small differences in the intrinsic response of the sub-populations to CCNU, hence confirming the dependence of the effect upon spheroid structure. Uptake of $^{14}$C-labelled CCNU by cells in different layers within spheroids is currently being measured. Spheroids have also been grown from human small cell and large cell anaplastic lung cancer with different cell packing and inter-cellular contact characteristics. Isolation of sub-populations of cells from these spheroids has been achieved using Percoll gradients and cell sorting with the vital stain Hoechst 33342.

### Use of an isotope uptake assay for predictive quantitation of lung tumour response

G. Walls, N. Fox & P. Twentyman

*MRC Clinical Oncology and Radiotherapeutics Unit, Hills Road, Cambridge CB2 2QH, UK.*

Isotope uptake assays have many potential advantages over clonogenic assays for predictive testing of human tumour response. Among these are no requirement for an absolutely single cell suspension, the totally objective nature of the data produced and the speed of obtaining the result. A recently described tritiated thymidine ($^3$HTdR) uptake assay (Friedman & Glaubiger, 1982, *Cancer Res.*, **42,** 4683) apparently overcomes the principal problem of such assays i.e. the contribution of normal tissue elements in the tumour to isotope uptake, by plating the tumour cells into dishes containing an agar base. Experiments have been carried out to measure the response of human small cell lung cancer (SCLC) xenografts by both a simplified version of this $^3$HTdR uptake assay and the "Courtenay" soft-agar cloning assay. Excellent agreement was found between the two assays in measurement of response to radiation and a range of cytotoxic drugs over 3 decades of cell kill. Radiation response curves for recently established cultures of human SCLC cells have been obtained using the isotope assay and were found to fall exponentially with no initial shoulder. The radiation response of a mouse tumour cell line however, showed an initial shoulder in both assay systems. Although normal human bone marrow cells do take up some $^3$HTdR, the amount is relatively small in comparison with SCLC cells. It is hoped that the isotope uptake method may be useful as a rapid predictive assay of tumour response using bone marrow and node biopsy specimens from patients with SCLC.

**Drug sensitivity of human glioma cells in culture – the effects of verapamil**

S. Merry, S.B. Kaye & R.I. Freshney

*Department of Clinical Oncology, University of Glasgow, Glasgow G12 9LX, UK.*

In previous studies we have identified one human glioma cell line sensitive to adriamycin (G-MCF) and two human glioma cell lines which are resistant to this drug (G-UVW and G-CCM). Our investigations of the intracellular accumulation of adriamycin in these cell lines have shown that both energy deprivation and the addition of verapamil are able to increase intracellular drug concentration by at least 100% in the resistant cell lines, but not in the sensitive cell line.

We now present data showing that similar doses of verapamil are able to increase significantly the cytotoxicity of adriamycin in the resistant cell lines. Cells were seeded into 24-well plates and, after 72 h, were exposed to adriamycin or adriamycin plus verapamil for a further 72 h followed by a recovery period of 120 h. Cell number at the end of this period was determined by trypsinisation and Coulter counting of the resultant single cell suspension. A decrease in the adriamycin $ID_{50}$ of at least 5-fold for the two resistant cell lines was observed upon the addition of verapamil with growth inhibition increased from 25% to 75% for G-CCM at $7\times10^{-8}$M adriamycin and from 50% to 90% for G-UVW at $3\times10^{-8}$M adriamycin. Further studies using other drugs are in progress.

These data suggest that for human tumour cells adriamycin resistance is related to differences in drug transport as has been demonstrated in animal models. Furthermore, a potential role for verapamil in circumventing the tumour cell resistance observed clinically is suggested.

**Structure-antitumour activity relationships in N-alkylformamide derivatives**

M.D. Threadgill, E.N. Gate, S.P. Langdon & M.F.G. Stevens

*Cancer Research Campaign Experimental Chemotherapy Group, Department of Pharmacy, University of Aston in Birmingham, Birmingham B4 7ET, UK.*

A series of N-alkylformamide derivatives of general formula $R^3C(X)NR^1R^2$ has been prepared where $R^1=H$, Me, $CD_3$, $CH_2CF_3$, $CH_2CH_2$,Cl, cyclopropyl, $CH_2OH$, $CH_2OEt$, $CH_2OAe$, $Ch_2OBz$, $CH_2NMe_2$; $R^2=H$, Me, $CF_3$, $CCl_3{}^+$ $R^3=H$, Ph, NHMe, $NMe_2$ and X=O, S, NH.

These compounds have been tested for activity against the M5076 ovarian sarcoma and the TLX5 lymphoma *in vivo* in mice. Against both tumours, N-methylformamide $R^1=Me$, $R^2=R^3=H$, X=O; NMF) was by far the most potent agent of the group. A putative metabolite of NMF, N-(hydroxymethyl)-formamide ($R^1=CH_2OH$, $R^2=R^3=H$, X=O) had, at most, marginal activity in these tests. In this series, it appears that an N-methyl group is a strong requirement and the formyl group is a lesser requirement for activity.

**Experimental antitumour activity and mechanism of action of the novel anticancer agent CCRG 81010**

P. Workman & F.Y.F. Lee

*MRC Clinical Oncology and Radiotherapeutics Unit, Cambridge CB2 2QH, UK.*

CCRG 81010 (8-carbamyl-3(2-chloroethyl)-imidazo(5,1-d)-1,2,3,5-,-tetrazin-4(3H)-one; M and B 39565, NSC 353451) is a novel antitumour agent with broad spectrum activity in standard preclinical screens (Stevens *et al.*, 1983, *Br. J. Cancer*, **48,** 120) and is currently undergoing phase 1 trial. We have determined the effect of CCRG 81010 against several syngeneic mouse tumours and human tumour xenografts, in direct comparison with certain nitrosoureas and the imidazole triazene DTIC, to which it exhibits structural similarities. Tumours were grown i.m. in the legs of C3H (mouse tumours) or MFI nude (xenograft) mice. Mice received single i.p. doses of drugs when tumours reached 8–10 mm diameter, and growth delay (GD) was used as the end-point. A linear dose-response to CCRG 81010 was seen in the KHT mouse sarcoma with 50 mg $kg^{-1}$ ($0.6\times LD50$) producing GDs of 12–17 days. Equitoxic doses of CCNU, methyl-CCNU and BCNU gave similar GDs, though chlorozotocin was less active (GD$\sim$7 days). In the KHT variant made resistant to nitrosoureas CCRG 81010 gave GDs of only 2–5 days similar to those in the intrinsically resistant RIF-1 mouse sarcoma. DTIC was equally inactive against all 3 mouse tumours. Cyclophosphamide showed comparable activity in KHT and RIF-1 but reduced activity in the KHT resistant tumour. Phenobarbitone pretreatment reduced the activity of CCRG 81010 against the KHT tumour, whereas SKF 525A and misonidazole were without effect. In human xenografts CCRG 81010 was similar to CCNU: Both were inactive against the colon carcinoma HT29 and the large cell lung tumour L23, but very effective against the small cell lung carcinoma H69. To summarise, CCRG 81010 has a spectrum of antitumour activity similar to that of CCNU but different to that of DTIC and cyclophosphamide.

**Low dose aminoglutethimide for advanced postmenopausal breast cancer**

B.M.J. Cantwell, R. Sainsbury, A.L. Harris, R.G. Wilson, J. Farndon, P. Dawes, R.G.B. Evans & M. Dowsett

*Departments of Clinical Oncology, Radiotherapy and Surgery, University of Newcastle upon Tyne, UK.*

Aminoglutethimide (AG) 125 mg twice daily is as effective as 250 mg 4 times daily in producing oestrone and oestrodiol suppression (Harris *et al.*, 1983, *Br. J. Cancer,* **47,** 621). Since the higher dose is an effective endocrine therapy we clinically assessed low dose AG. Doses used were 125 mg twice daily plus hydrocortisone (HC) 20 mg twice daily to treat advanced postmenopausal breast cancer. So far 38 patients with advanced progressive breast cancer have been entered into study and 30 patients followed for 3 months or longer. The response to AG+HC by U.I.C.C. criteria assessed after 3 months is 1 complete response (8 months+duration) 7 partial responses (4,4+, 6,7,7,7+ and 11+ months duration). Nine patients have achieved stable disease (SD) (4,4+,5+,6,6,7+,7+,7+ and 7+ months duration). This gives an overall objective response rate of 27% (8/30). Mean age in responders (objective and stable disease combined) was 60.9 years and was 62.3 years in non-responders. There was no significant difference in age, years after menopause, disease free interval, weight and predominant sites of disease at start of AG+HC therapy. In 12 patients who did not respond to Tamoxifen before AG+HC therapy, there were 3 (1 PR, 2 SD) who subsequently responded to low dose AG+HC. In 6 patients who had a prior response to Tamoxifen therapy there were 3 (2PR, 1 SD) subsequent responses to low dose AG+HC. Toxicity with low dose AG+HC was considerably less than previous experience with higher doses (Harris *et al.*, 1983, *Eur. J. Cancer Clin. Oncol.*, **19,** 11), nausea and vomiting occurring in 3% of patients on the low dose schedule *versus* 15% with higher dose AG (rash incidence was the same in both low and higher dose groups). Low dose AG produced an objective response rate comparable to conventional dose with considerably less toxicity.

**A comparison of two doses of trilostane combined with dexamethasone in metastatic breast cancer**

A.C. Hindley[1], P.M. Wilkson[1], J. St. John[1], C.G. Beardwell[3] & G.G. Ribeiro[2]

[1]*Departments of Clinical Pharmacology,* [2]*Endocrinology and* [3]*Radiotherapy, Christie Hospital, Manchester, UK.*

Trilostane (T) a steroid agent, is an inhibitor of adrenal enzymes in the rat. We have shown that a combination of (T) 240 mg qds and Dexamethasone 0.5 mg bd induce remission in metastatic breast cancer (Beardwell *et al.*, 1983, *Cancer Chemother Pharmacol.,* **10,** 158). The principle hormonal changes occurring in those patients on (T) alone, were significantly elevated serum concentrations of 170H pregnenolone, dehydroepiandrosterone sulphate and androstenedoine, with significantly reduced levels of oestrone. After the addition of Dexamethasone, levels of these hormones returned to baseline, with the exception of oestrone, which remained depressed. The response in all patients receiving therapy for >eight weeks was 10/31 PR (32%) and 3/31 (10%) stable disease by UICC criteria. Gastrointestinal side effects were common, vomiting occurring in (24%) and diarrhoea in (11%) and therapy was withdrawn in (18%). Nineteen patients subsequently received (T) 120 mg qds with Dexamethasone 0.5 mg bd. The incidence of side effects were greatly reduced, but an objective response was observed in only one patient which suggests that the lower dose may be ineffective, although initial results suggest that the hormonal effects on (T) alone are similar for both doses. The mechanism of action of the combination is presently undefined, but the differences in response with the two doses of (T) suggests that the anti-tumour is not due to Dexamethasone alone. Further studies are in progress to determine the optimum dose and frequency of administration.

**Pharmacokinetics and metabolism of mitoxantrone**

J.S. Macpherson, J.F. Smyth, J.A. Clements[1], M.W. Ramsay, P.S. Warrington, R.C.F. Leonard & C.R. Wolf

*Imperial Cancer Research Fund, Medical Oncology Unit, Western General Hospital,* [1]*Department of Pharmacology, Heriot-Watt University, Edinburgh, UK.*

Mitoxantrone (M) is an anthraquinone derivative which we are currently evaluating for the treatment of breast cancer. We have established the following HPLC assay to determine M concentrations in plasma and urine. Plasma or urine samples were applied directly to a pre-wet $C_{18}$ Sep-Pak cartridge, washed with 10 mM HCl and the sample eluted with 100% methanol. The recovery of M for both plasma and urine samples was almost 100%. The sample was then evaporated to dryness and reconstituted in the HPLC running buffer of ammonium formate/acetonitrile 73:27 (ph 4.3). M was then separated on a $\mu$ Bondapak $C_{18}$ column and drug concentration monitored at 658 nm. The detection limit was 1 ng ml$^{-1}$ plasma. Evaluation of the data obtained so far from 6 patients showed the plasma clearance follows 3 phases, with $T^{\frac{1}{2}}$ values of $\alpha = 10.7 \pm 2.2$ min, $\beta = 2.0 \pm 0.5$ h and $\gamma$ of $17.0 \pm 5.7$ h. Urinary excretion of M in the first 24 h was $3.0 \pm 1.6$% of the dose. In addition to unchanged drug, 2 metabolites were detected. UV-Vis spectra indicated that both contained an intact anthraquinone nucleus. The retention time of these products was not altered on incubation with $\beta$-glucuronidase or sulphatase suggesting the presence of either primary oxidation products or conjugates other than glucuronide or sulphate. In contrast to this finding initial experiments *in vitro* using rat liver fractions showed 2 primary metabolites to be formed. Both of these products had different retention times on HPLC to the metabolites found in the urine. The formation of one of these products was dependent on the presence of the cofactor required for glucuronidation (UDPGA). The second major product required the presence of cytosolic enzymes. If these products are found in man it would appear that they are either further catabolised or excreted in the bile.

### Controlled trial of adjuvant chemotherapy with cyclophosphamide methotrexate and fluorouracil (CMF) for breast cancer

A. Howell, H. Bush, W.D. George, J.M.T. Howat, D. Crowther, R.A. Sellwood, R.D. Rubens, J.L. Hayward, R.D. Bulbrook, I.S. Fentiman & M. Chaudary

*Departments of Medical Oncology and Surgery, University Hospital of South Manchester and Guy's Hospital, London, UK.*

In 1976 Bonadonna *et al.* (*NEJM*, **294**, 405) reported that adjuvant CMF prolonged the relapse free survival in patients with positive axillary nodes. It was decided to repeat this study in Manchester starting in March 1976 and at Guy's Hospital starting in October 1979. In each centre patients with operable breast cancer (TO-3,NO,1,MO) were treated with total mastectomy and complete axillary clearance. Patients with involved axillary lymph nodes aged <70 years were allocated randomly to either no additional treatment (n = 162) or 12 monthly cycles of CMF (n = 165). Each cycle consisted of cyclophosphamide 80 mg m$^{-2}$ p.o. on days 1–14, methotrexate 32 mg m$^{-2}$ i.v. on days 1 and 8 and fluorouracil 480 mg m$^{-2}$ i.v. on days 1 and 8. There were no significant disparities in the distribution of prognostic variables between treatment and control groups. There was a highly significant difference in relapse-free survival in favour of CMF ($P = 0.005$) after a median duration of follow up of three years. Recurrence rates were compared for subsets of the pooled data. Significant differences between treated and control patients were seen in those with 1–3 nodes involved ($P = 0.04$), >4 nodes ($P = 0.02$) and also in premenopausal patients ($P = 0.02$) but not in postmenopausal patients ($P = 0.13$). There was no significant difference in overall survival between the two groups. Side effects were common and included nausea and vomiting (65%), myelosuppression (70%) and alopecia (20%).

We conclude that CMF significantly delays recurrence but, at least, early in the follow up period has no effect upon survival.

### Epidermal growth factor and oestrogen receptors in human breast cancers

J.R.C. Sainsbury, J.R. Farndon & A.L. Harris

*Departments of Surgery and Clinical Oncology, University of Newcastle upon Tyne, Newcastle upon Tyne, UK.*

Epidermal growth factor (EGF) is a mitogen for many tissues and its effects on cultured breast cells include stimulation of DNA synthesis. Receptors for EGF have been described on human derived cell lines such as MCF7 and A431. EGF was as potent as oestrogen in causing cell growth. Oestrogen receptors (ER) have been found in 50–65% of breast cancers and are of predictive value in survival and response to therapy.

The aim of this study was to detect and quantify EGF receptors on breast cancer specimens and to compare this to ER status. Biopsies were taken fresh, collected on ice and a membrane pellet prepared by a differential centrifugation technique using a TRIS-NaCl-BSA buffer. Receptors were identified by a competitive binding method using 0.6 nmol l$^{-1}$ $^{125}$I-EGF incubated with varying concentrations (0.025–500 nmol l$^{-1}$) of cold EGF. Nuclear and cytosolic ER were measured by a dextran-coated charcoal technique. High affinity EGF receptors were detected with a Kd of 0.7–$1.5 \times 10^{-9}$M 33 primary breast cancers have been studied. Nineteen were ER+ve (57%), 6 (18%) were EGF+ve, 4 of these were ER−ve and 2 ER+ve. Five out of six metastatic lymph nodes studied were EGF+ve and 1 EGF−ve: all were ER−ve. The addition of anti-proteolytic agents did not affect the results.

These results show that some human breast tumours express EGF receptors. The negative correlation with ER status suggests that these tumours may have a poorer prognosis. Since EGF stimulates anchorage independant growth the higher incidence of EGF positive tumours ($P < 0.001$, Fisher's exact test) in lymph nodes suggest that EGF may have a role in metastasis.

**Clinical correlations in ovarian cancer using the clonogenic assay**

A.P. Simmonds & E.C. McDonald

*Cell Laboratory, Biochemistry, Royal Maternity Hospital and Royal Infirmary, Glasgow, UK.*

Culture of ovarian tumour material has been continued as previously described (Simmonds & McDonald, 1982, *Br. J. Cancer*, **46**, 462) using 200 samples from 106 patients in the Glasgow area. Drugs tested were cis-platinum and adriamycin using the parameters for sensitivity of Alberts, Salmon *et al.*, 1980. *Lancet*, 340). Clinical follow-up was achieved courtesy of the West of Scotland Clinical Trials Unit, Department of Oncology and consultant staff in the gynaecology and oncology departments of the hospitals involved. Results obtained in the laboratory were then compared with clinical outcome. Of the 57 patients represented by the 71 samples available for drug study, only 48 received chemotherapy, of whom 23 received the drugs of test. 10/11 patients judged sensitive to cis-platinum received it in some form, with 7 partial and 3 complete responses. 5/6 patients sensitive to adriamycin received it and had a good response. Equivocal results were obtained with patients with intermediate responses to cis-platinum, even in those with known sensitivity to adriamycin. Five of 6 patients resistant to cis-platinum received it. Three were also resistant to adriamycin and have failed to respond to CAP chemotherapy. Two patients with complete responses to the CAP regime had unknown responses to other elements of the combination. Clear relationships were observed between cis-platinum resistance *in vitro* in untreated patients and a failure to respond to other chemotherapy. Cis-platinum and adriamycin sensitivity *in vitro* were clearly related to clinical outcome, but no relationship exists between such sensitivities and response to other chemotherapy.

**A bis-benzimidazole probe for chromatin structure and cellular sensitivity to DNA damage**

P.J. Smith & J.V. Watson

*MRC Clinical Oncology and Radiotherapeutics Unit, MRC Centre, Cambridge CB2 2QH, UK.*

Transcriptionally active or potentially active regions of chromatin demonstrate enhanced sensitivity or accessibility to endonucleases. They also provide preferred sites for the direct or enzyme-mediated effects, on DNA, of various agents used in tumour therapy: e.g. bleomycin, ionising radiation, nitrosoureas and actinomycin D. We have investigated whether cellular responses to DNA damage can be related to this feature of chromatin in intact human cells by using a DNA-specific ligand (Hoechst 33342) as a vital-probe for chromatin structure. The appearance of ligand-induced DNA strand-breaks was related to Ho33342 uptake (measured by flow cytometry). However, compared with radiogenic DNA strand-breaks, Ho33342-associated damage disappeared slowly. The endonuclease (DNase II) sensitivity of nuclear DNA in permeabilised cells correlated with the distribution and level of ligand-induced DNA strand-breakage. Cellular responses (lethality and the retention of cells in G2 phase) to DNA damage induced by either the ligand or a broadspectrum DNA damaging agent (X-radiation) could be ascribed to the degree of expression of enzyme-sensitive targets in chromatin. Enhanced target expression was found in cells derived from a patient with the cancer-prone, radiation-hypersensitivity disorder ataxia telangiectasia. Our studies provide some insight into the laboratory hallmarks presented by the disorder ataxia telangiectasia and indicate how the biological responses of a given tumour cell type to therapy-induced DNA damage may be ascribed and perhaps predicted from studies on chromatin structure and organization using ligand or enzymatic probes.

### Precise epitope mapping of mouse tumour-associated protein p53

A. Wade-Evans & J.R. Jenkins

*Marie Curie Research Institute, The Chart, Oxted, Surrey, UK.*

p53 is a cellular-encoded, transformation related protein, present at elevated levels in numerous transformed cell lines (Linzer *et al*, 1979, *Cell*, **17**, 43).

In order to study the function of p53 in detail, a full length cDNA clone has been isolated in this laboratory (Jenkins *et al.*, manuscript in preparation). Using appropriate recombinant DNA techniques the coding region of the p53 clone has been inserted into plasmid vectors, which direct *in vitro* expression of the protein, both by itself, and as a fusion protein with SV40 small t.

A combination of *in vitro* transcription, translation in a cell-free reticulocyte lysate system, immunoprecipitation using p53-specific monoclonal antibodies, and Bal 31 deletions of the gene, have enabled us to map accurately the epitopes of several different monoclonal antibodies.

The resulting data have allowed us to construct synthetic peptides, which are complimentary to the corresponding antigenic sites on the p53 protein. The combination of anti-p53 monoclonals together with their complementary synthetic peptides is being used as an immunochemical step in the preparative isolation of p53 protein from genetically engineered primate cell lines, expressing mouse p53 as high levels.

The epitope mapping data also provide insights into the structural associations that p53 may adopt in the cell.

### Recombinant DNA directed expression of the transformation-associated protein p53

J.R. Jenkins, K. Rudge, K. Barrett & A. Wade-Evans

*Marie Curie Research Institute, The Chart, Oxted, Surrey, UK.*

We have cloned full length cDNA sequences coding for the murine transformation-associated protein p53. Using appropriate recombinant DNA techniques we have constructed expression vectors which direct the efficient synthesis of murine p53 protein in eukaryotic systems. These expression vectors are being used to investigate the mechanisms of cellular localization of p53 and its association with SV40 large T antigen in a variety of normal and transformed cells.

### Transforming activity of a cellular proto-oncogene after *in vitro* modification by a chemical carcinogen

D.H. Phillips, C.J. Marshall & K.H. Vousden

*Institute of Cancer Research, Fulham Road, London SW3 6JB, UK.*

Activated transforming genes have been found in a number of human tumour DNA's using the NIH/3T3 transfection assay. In most cases the genes detected are members of the cellular ras gene family. The mechanism by which they have been activated in tumour cells has been shown to be a single point mutation resulting in the alteration of an amino acid in the p21 protein product. We have investigated the effect of modifying the normal cellular c-Ha-ras-1 gene by treatment with (±)anti-7,8-dihydroxy-9,10-epoxy-7,8,9,10-tetrahydrobenzo-(a)pyrene (BPDE). pEC, a plasmid containing an insert that carries the c-Ha-ras-1 gene, in 10 mM tris-HC1, 1mM EDTA, ph 7.4 ($0.5\,\mu g\,\mu l^{-1}$) was treated with various concentrations of BPDE in EtOH (0.5 vol) at 37° for 2 hours. DNA was purified from the reaction mixture, calcium-phosphate precipitated and used in an NIH/3T3 transfection assay (200–1000 ng/plate). After 2 weeks the plates were scored for transformed foci. From DNA treated with BPDE at a ratio of 15:1, 1.7–2.3 foci/μg DNA were observed. Transformed foci were picked and the DNA isolated from them was used in a second round of transfection into NIH/3T3 cells. DNA isolated from the secondary transformants was subjected to Southern blot analysis using a variety of restriction enzymes. Four transformation events have been analysed so far and in one case an Msp I restriction site has been altered. A change in sequence here is predicted to lead to an alteration in the amino acid at position 12 of the gene product. These results demonstrate that *in vitro* modification of a cellular proto-oncogene with a chemical carcinogen can lead to an active transforming gene. This provides direct evidence that DNA is the primary target of chemical carcinogen-mediated malignant transformation.

### Transforming genes in human malignant melanoma

R.A. Padua & N. Barrass

*Marie Curie Research Institute, The Chart, Oxted, Surrey, UK.*

DNA, isolated from 7 surgical specimens of malignant melanoma and 6 established cell lines, was tested for its ability to transform NIH3T3 mouse fibroblasts by transfection. Positive results were obtained from one of the primary tumour DNAs and 3 of the cell lines. Human sequences were identified in transformant DNAs by hybridization with an *Alu* human repetitive sequence clone. Cloned oncogene probes were used to identify the genes activated in the transformants obtained. An activated N-ras gene was identified in one of the cell lines. In the case of the primary tumour and two of the cell lines transformants were obtained which showed no detectable homology to the three known ras genes, or to any other probe in a comprehensive oncogene library.

### The SV40 transformed human breast epithelial fR cell lines contain a deletion in their SV40 large T-antigen

S.E. Chang

*Marie Curie Research Institute, The Chart, Oxted, Surrey, Jacqueline Keen and Joyce Taylor-Papadimitriou, Imperial Cancer Research Fund, Lincoln's Inn Fields, London, UK.*

The availability of the fR series of SV40 transformed breast epithelial cell lines (Chang *et al.*, 1982, *Cancer Res.*, **42,** 2040) gives us a unique opportunity to study how SV40 can be integrated in the DNA of human epithelial cells. DNA mapping experiments using restriction enzymes and southern blot techniques show that one SV40 genome is integrated in these transformed cells and that there is a deletion of up to 100 base pairs in the region coding for large T-antigen. The sites of integration and deletion are coincident and map in the region 4000–4100. The $NH_2$-terminal end of the large T-antigen is intact, a finding consistant with the idea that this region may be essential for transformation (Colby & Shenk, 1982, *PNAS*, **79,** 5189; Clayton *et al.*, 1982, *Nature*, **299,** 59).

Since K14, an SV40-transformed human keratinocyte cell line (Taylor-Papadimitriou *et al.*, 1982, *Cell Diff.*, **11,** 169) also has a deletion in its SV40 large T-antigen, it is possible that a casual relationship exists between deletions in the large T-antigen and the ability of SV40-transformed human epithelial cells to survive "crisis" and to give rise to immortal cell lines.

### Monocytic cells in lymphomata

S. Matthews, A.J. Freemont, C.J.P. Jones & R.W. Stoddart

*Pathology Department, University of Manchester Medical School, UK.*

Cells apparently derived from the monocyte lineage have been identified in lymphomata, but their function remains obscure. Using immunocytochemical, lectin binding and morphological criteria we have attempted to characterise these cells more fully. Paraffin embedded lymphomatous and reactive lymph nodes have been examined for $\alpha_1$-anti-trypsin ($\alpha_1$at) and lysozyme, using polyclonal antibodies, and sugar residues, using several lectins including concanavalin A (conA) and wheat germ agglutinin (WGA) by PAP, fluorescent lectin and avidin-biotin peroxidase techniques. Two subgroups of cells have been identified which, in lymphomata, have distinctive distributions. The first consists of rounded cells 15–20 $\mu$m in diameter which react strongly for lysosyme and $\alpha_1$at and bind conA and WGA avidly. They resemble blood monocytes and cluster about nodules of both Hodgkins and non-Hodgkins lymphomata. The larger cells in the second subgroup have long cytoplasmic processes embracing neighbouring cells, stain weakly for lysozyme and $\alpha_1$at and bind conA weakly. The role of these groups is speculative. The first might be either a primary response or one secondary to T lymphocyte activation and either may be forcing a nodular growth pattern on the tumour. The second could represent either a response to the lymphoma or recruitment of cells necessary for some aspect of tumour behaviour. These, and other possibilities, are being investigated.

### Characterisation of mononuclear cell infiltrates, by monoclonal antibodies, in colorectal carcinomas, polyps and the adjacent normal bowel

D. Heineman, H.C. Umpleby, M.O. Symes & R.C.N. Williamson

*Department of Surgery, University of Bristol, Bristol, UK.*

Serial frozen sections were prepared from a colorectal carcinoma and the adjacent normal bowel in 10 patients and from a benign polyp in a further 10 patients. Six of the patients with a polyp had a concomitant carcinoma. The mononuclear cell infiltrates were stained by the indirect immunoperoxidase technique using a panel of mouse monoclonal antibodies to human leucocyte antigens. The degree of infiltration was graded 5 (heavy), 4 (moderate), 3 (few), 2 (occasional) and 1 (nil). Assessment was performed by two independent observers.

The colorectal carcinomas and adjacent normal bowel showed an equal degree of leucocyte infiltration, graded 4–5 in 7 cases (Hele-4). In a further patient infiltration was graded 3 in each tissue. In 7 of the carcinomas cytotoxic/suppressor T lymphocytes (UCHT-4) graded 3–4, predominated over helper cells (OKT-4), graded 1–2. By contrast in the adjacent normal bowel cytotoxic and helper cells were present in equal numbers, graded 2, in 7 patients. Of the 10 polyps leucocyte infiltration was graded 5 in 9 and 4 in 1. In 9 of the 10 polyps cytotoxic cells graded 3 predominated over helper cells graded 1–2. The number of helper cells was equal in all 6 concomitant polyps and carcinomas. The similarity in cell infiltrate between polyps and carcinomas is in favour of the polyp carcinoma sequence (Morson, 1974, *Proc. R. Soc. Med.*, **67**, 451) and the predominance of cytotoxic cells in tumour tissue accords with the association between lymphocytic infiltration in a tumour and a favourable prognosis (Hutchinson *et al.*, 1983, *J. Exp. Clin. Cancer Res.*, **2**, 161).

### Immunoperoxidase staining of primary breast tumours with an antibody against human milk fat globule antigen predicts survival and response to hormone therapy

M.J.S. Wilkinson, A. Howell, J. Taylor-Papadimitriou, D. Barnes, R. Swindell & R.A. Sellwood

*Departments of Surgery, Medical Oncology and Clinical Research, Christie Hospital, Manchester and Imperial Cancer Research Fund, Lincolns Inn Fields, London WC2, UK.*

There is a need for a simple procedure performed on the primary tumour of patients with cancer of the breast which give prognostic information. The value of such a procedure would be increased if it predicted response to endocrine therapy. We have assessed the predictive value of the staining of tumours with antibody raised against a component of the milk fat globule membrane. (Arklie *et al.*, 1981, *Int. J. Cancer*, **28**, 23). A monoclonal antibody (HMFG-1) was raised against delipidated human milk fat globule membranes and used to stain 200 primary breast tumours by an indirect immunoperoxidase technique. Several staining patterns were seen but the presence or absence of antigen in the spaces outside cells (ECS) was of most prognostic importance. Of the 200 patients studied 79 relapsed and 51 received endocrine therapy, 41 were assessable for response of whom 41% responded. Patients whose tumours showed ECS staining had a significantly longer relapse free interval ($P<0.008$) and survival ($P<0.002$). 65% of patients who relapsed and were ECS+ve responded to hormone therapy whereas 33% of ECS−ve responded. The median time to progression of all patients with ECS staining tumours was 8 months and 3 months for ECS−ve tumours. The figures for progesterone receptor +ve and −ve patients was 9 months and 3 months respectively.

We conclude that the presence of the milk fat globule antigen, HMFG-1 outside the tumour cell and presumably shed from the cell membrane is related to a good prognosis and if the patient does relapse the likelihood of response to endocrine therapy is high and equivalent to that seen in progesterone receptor-positive tumours.

### Prediction of relapse free interval in patients with malignant glioma using a chemosensitivity test

J.L. Darling, D.G.T. Thomas, G.P. Finn, R.I. Freshney & E. Paul

*Gough Cooper Department of Neurological Surgery, Institute of Neurology, Queen Square, London WC1 and Department of Clinical Oncology, Glasgow UK.*

One hundred and seventeen patients with malignant glioma (Kernohan grades III and IV) were treated with adjuvant chemotherapy comprising procarbazine (PCB) 50 mg t.d.s. for ten days, CCNU 80 mg stat and vincristine (VCR) 1.4 mg m$^{-2}$ stat, following whole head irradiation fractionated to a total dose of 4000–5000 cGy. Cell cultures were preprared from 40 patients in this series and their sensitivity to each of the drugs was assessed in a microtitration assay with $^{35}$S-methionine incorporation as the end point. Sensitivity to PCB or CCNU was linked with increased relapse free interval (RFI) whilst sensitivity to VCR was not. It was found that 22/40 (55%) of patients responded to PCB and/or CCNU in the assay. The RFI of patients who had responded to PCB or CCNU *in vitro* was significantly longer than both the RFI of patients whose tumours failed to respond *in vitro* and the RFI of those patients who had not been tested. This difference was not attributable simply to an imbalance in prognostic factors between the groups. However, it was apparent that grade of tumour influenced chemosensitivity *in vitro*. Patients with grade III tumours responded well *in vitro* (16/22 patients sensitive to PCB or CCNU) whilst only 6/22 patients with grade IV tumours responded to either of these drugs. It is hoped that this assay may form the basis of a prospective randomised trial of chemosensitivity testing.

### Improved *in vitro* determination of tumour chemosensitivity in haematological malignancies

M.C. Bird, A.G. Bosanquet & E.D. Gilby

*Department of Clinical Investigation, Royal United Hospital, Combe Park, Bath BA1 3NG, UK.*

We have further developed our 4-day chemosensitivity assay previously described (Bosanquet *et al.*, 1983, *Br. J. Cancer*, **47**, 781) so that cell identification is greatly improved and problems of cell autolysis and proliferation are overcome. After continuous incubation of drug with isolated white cells, dead cells are stained with a mixture of fast green FCF and nigrosin and the cultures cytocentrifuged. Remaining live cells are counter-stained with Romanowsky stain. A known number of fixed duck erythrocytes (DRBC) is added to the stain as an internal standard. For quantification of results the ratio of live tumour cells to DRBC is determined for each slide and the ratio in drug-tested samples expressed as a percentage of that in control samples (TCV). Over 30 drugs have been tested in the assay, and all have given a dose-response relationship and a good scatter of sensitivities between patients. Chemosensitivity of blood and bone marrow samples was similar where both were tested together. Twenty-three of 32 tests (72%) undertaken with chronic lymphocytic, acute lymphoblastic and acute myeloid leukaemias and multiple myeloma have been technically successful. Assay results could be correlated with *in vivo* response in 12 cases. Three were sensitive both *in vitro* and *in vivo*, 8 were resistant *in vitro* and *in vivo* and one was sensitive *in vitro* but resistant *in vivo*.

### Patient intolerance to superficial hyperthermia

R.D. James & D. Pye

*The Christie Hospital & Holt Radium Institute, Manchester, UK.*

The discomfort associated with superficial hyperthermia is of two types. The first is due to prolonged immobilisation. The second is due to pain in the skin from high temperatures. Iso-effective treatments using various times and temperatures can be derived from animal work. We have studied the tolerance of 25 patients to a variety of hyperthermia schedules, thought to be iso-effective for normal tissues. Of 30 treatments using times in excess of 30 min, 10 were discontinued because of patient intolerance. Of 23 treatments using times of less than 10 min only one was discontinued. We believe the optimum schedule for clinical treatment should not exceed 10 min, and should aim for a tumour temperature of at least 45°C. Skin pain can generally be controlled using skin cooling or local anaesthetic.

### Bone marrow repopulation following high dose radiotherapy using indium-111 scanning

P.E. Cummins, R.D. James[1], S. McKenzie & P.M. Nuttall

*Regional Department of Medical Physics and [1]Department of Radiotherapy, Christie Hospital Manchester, UK.*

Indium-111 bone marrow scanning has been used to assess marrow damage and the extent of post therapy recovery in patients with Hodgkin's disease treated with mantle radiotherapy. The interval between completion of treatment and time of scanning varied from 3 months to 14 years. Bone marrow scans were performed 3 days after i.v. injection of 74 MBq (2 mCi) indium-111 choride using a large field of view gamma camera. All scans were recorded by computer on magnetic disc for later retrieval and processing. The scans were analysed quantitatively by measuring irradiated bone to soft tissue ratios and irradiated to un-irradiated bone uptake ratios. These ratios – all determined with the region of interest (ROI) technique – correlated significantly with time after irradiation. Results indicate very slow repopulation of irradiated marrow which returns to normal after a period of 10 to 15 years. It is suggested that slow repair of bone marrow stromal elements may be the cause of this delayed repopulation.

**At mastectomy tumour size and axillary node status predict time to relapse and steroid hormone receptors predict survival after first relapse**

R.N.L. Harland, A. Howell, D.M. Barnes, M.J.S. Wilkinson & R. Swindell

*Departments of Surgery, Medical Oncology, Clinical Research and Statistics, Christie Hospital, Manchester, UK.*

We found that neither oestrogen receptor (ER) nor progesterone receptor (PR) status of the primary tumour predicted time to relapse after mastectomy but receptor positive tumours were associated with a greater survival. This suggested that the survival advantage was due to a greater survival after relapse in patients with receptor positive tumours. We have therefore examined the effect of other prognostic factors (tumour size and axillary node status) as well as receptors upon overall survival, time to first recurrence after mastectomy and survival after first relapse. ER was measured in 508 and PR in 496 primary tumours by the dextran coated charcoal technique. With the exception of 32 treated with melphalan no additional treatment was given after mastectomy. The median follow up was 36 months and the maximum was 91 months. One hundred and thirty-seven patients relapsed and the response to endocrine therapy was known in 65.

All variables indicated groups with different probabilities of survival after mastectomy (size T classification), $P<0.005$; nodal status, $P<0.02$; ER status, $P<0.0001$; PR status, $P<0.002$. Both size and node status identified groups with differing probabilities of relapse (both $P<0.0001$), but similar probabilities of survival after relapse. Conversely neither ER nor PR status indicated the probability of relapse although groups with markedly different prospects of survival after relapse were identified (ER and PR $P<0.0001$). Thus greater survival after relapse was related to the group of patients with receptor positive tumours who responded to endocrine therapy. We conclude that size and node status predict time to relapse and receptor status predicts survival after relapse in patients with cancer of the breast.

**Nandrolone decanoate (N.D.) as an adjunct to chemotherapy in the treatment of advanced carcinoma of the breast**

R.L. Turner, M.C. Bibby & D.M. Robertshaw[1]

*Clinical Oncology Unit, University of Bradford and [1]Biochemistry Department, Bradford Royal Infirmary, Bradford, UK.*

In 1959, Whyte *et al.* (*Br. med. J.*, **i**, 1315) observed a synergistic effect when testosterone was administered with thiotepa in advanced breast cancer. They postulated that testosterone was unlikely to cause androgenic inhibition of tumour growth but rather to stimulate erythropoiesis and bone marrow "protection". This hypothesis is now tested with N.D., an anabolic steroid with ~6 times the anabolic potency of testosterone but with considerably fewer androgenic side effects. An open controlled study was designed to investigate the effect of N.D. as an adjunct to a standard cytotoxic regime (Methotrexate and Thiotepa) on haemopoiesis, host immunity and chemotherapeutic response. Forty-five female advanced breast cancer patients were randomised for either cytotoxic therapy plus N.D. or cytotoxics alone. Full haematological and biochemical assessments were made at baseline and at weekly intervals up to day 63. Lymphocyte typing and immunoglobulin measurements were performed at baseline, days 28 and 56. N.D. accelerated the recovery of red cell haematological parameters following the depression caused by cytotoxic therapy. Recovery was complete in the N.D. group by the end of the study period, 7 weeks after the cessation of the first course of chemotherapy but not in the control group. There was no evidence of renal or hepatic damage by N.D. and only minor occurrences of virilising side effects. The difference in remission rates between N.D. and control groups was quite striking. Three times as many N.D. patients achieved complete remission when compared with controls. It is not clear at present whether this is due to an enhancement of cytotoxic action or to a more direct anabolic effect either upon the host-immune response or upon the metabolic status of the patients or indeed a combination of both.

**The effect of oestrogen and progesterone receptors on recurrence and survival in patients with breast cancers**

J.M.T. Howat, D.M. Barnes, M. Harris & R. Swindell

*North Manchester General Hospital & Christie Hospital & Holt Radium Institute, Manchester, UK.*

Patients with breast cancer and oestrogen (REc) and progesterone (RPc) receptors in their tumours generally survive longer than those who lack them, but whether the beneficial effect of receptors results from a longer disease-free interval (DFI) or greater survival after relapse is not clear.

Recurrence and survival rates were studied in 175 women with primary breast cancer who, until recurrence, received no treatment other than a modified radical (Patey) mastectomy. The REc and RPc content of the primary tumours was measured by the dextran-coated charcoal method. At a median follow-up of 47 months (range 55–58), overall there was no difference in the DFI of REc+ve and −ve patients although there was a significant increase in the postmenopausal group ($P=0.02$) and for those with 1–3 involved axillary nodes ($P=0.02$). The status of RPc did not affect the DFI in any patients.

Survival following mastectomy was significantly better for REc+ve patients ($P=0.006$), especially those who were postmenopausal ($P=0.003$) or node +ve ($P=0.01$). Survival was also increased for RPc+ve patients ($P=0.04$) and this too was evident in postmenopausal patients ($P=0.01$).

There were significant differences in the post-relapse survival (PRS) of REc and RPc+ve and −ve patients (REc $P=0.03$, RPc $P=0.001$) and patients with both REc and RPc survived approximately 17 months longer than their receptor-negative counterparts.

This study failed to confirm that measurement of REc and RPc can predict early relapse in breast cancer and the greater overall survival of receptor-positive patients is mainly due to an increase in PRS. This may reflect the response of receptor-positive patients to endocrine therapy which the majority received when they developed recurrent disease.

### A pilot study to evaluate the psychiatric morbidity in patients with advanced breast cancer

P. Hopwood, A. Howell & G.P. Maguire

*Department of Psychiatry, University Hospital of South Manchester, Withington and Department of Medical Oncology, Christie Hospital, Manchester, UK.*

Although there has been extensive research into psychosocial sequelae of treatments of early breast cancer little attention has been paid to psychiatric disorders in patients with advanced cancer of the breast. The aim of this prospective study was to determine the psychiatric morbidity in a group of 26 patients immediately before and during first line chemotherapy. Patients underwent a standard psychiatric interview by a Clinical Psychiatrist before and after 2–3 months of treatment. Depressive illness or anxiety states requiring further intervention was found in 9 patients (35%), occurring before chemotherapy in 5 (19%) and during chemotherapy in 4 (15%). The psychological disorder was equivalent to that found in patients attending a psychiatric outpatient clinic. The illness responded to anxiolytic or antidepressant medication in 6 patients, in two the disorder resolved as they responded to chemotherapy and one patient died from metastatic disease. We conclude that over one third of patients presenting with advanced breast cancer may have psychiatric morbidity and that this is amenable to treatment. Our current approach is to screen all patients with the aid of a questionnaire and to interview those with high scores.

### The treatment of bladder carcinoma with tumour-immune pig lymph node cells – a phase I study

M.O. Symes, H. Eckert, R.C.L. Feneley, J.C. Gingell, T. Lai, J.B.M. Roberts, P.J.B. Smith & C.R. Tribe

*Departments of Surgery, Radiotherapy and Pathology, University of Bristol and Bristol and Weston and Southmead District Health Authorities, UK.*

A total of 77 patients with invasive TCC of the urinary bladder, each received a single arterial injection of tumour-immune pig lymph node cells (LNC), into the tumour blood supply. The patients were divided into 4 groups: (1) those receiving pig LNC as the only treatment (16 patients); (2) those who received pig LNC on relapse (judged by EUA cystoscopy and biopsy) following radical radiotherapy, 5500 cGy (34 patients); (3) those who received pig LNC followed after an interval of 6 weeks by radiotherapy (4000 cGy) (10 patients); (4) as in group (3) but with the dose of radiotherapy increased to 5500 cGy (17 patients). Complete remission was characterised by complete disappearance of the tumour (for a varying time) following treatment. Partial remission was defined as: a reduction in the level of symptoms and a decrease in tumour size on EUA and/or cystoscopy. There were 1 complete and 3 partial remissions among the patients in group (1), 5 complete and 7 partial in group (2), 6 complete and 2 partial in group (3) and 8 complete and 1 partial in group (4). Of the 20 patients showing a complete remission, 7 lived for more than 5 years after treatment.

### Methodology for assay of thiotepa in plasma and initial pharmacokinetic studies

B.J. McDermott, J.A. Double, M.C. Bibby, D.E.V. Wilman[1], P. Loadman & R.L. Turner

*Clinical Oncology Unit, University of Bradford and [1]CRC Laboratories, Institute of Cancer Research, Sutton, Surrey, UK.*

ThioTEPA is being used currently in a controlled randomized trial to assess the role of nandrolone decanoate in combination regimes for advanced ovarian and breast cancer. Clinical findings (Turner, 1981, *Excerpta Med. APCS,* **9,** 1) and experiments with animal model systems (Double *et al.,* 1981, *Br. J. Cancer,* **44,** 305) indicated that the anabolic steroid increases the tolerance of bone marrow to chemotherapy. In addition, a pharmacokinetic interaction may occur and for purposes of this investigation, an assay for thioTEPA and its metabolite, triethylenephosphoramide (TEPA), was developed. After extraction from plasma using SEP-PAK $C_{18}$ cartridges, the compounds were separated by capillary gas chromatography, detected using a nitrogen detector and quantified by reference to an internal standard, hexaethylphosphoramide. Analytical recoveries were 73.6% and 95.2%, intra-assay variations (s.e.) were 5.6% and 7.5% (n = 12), and inter-assay variations (s.e.) were 8.8% and 12.5% (n = 6) for TEPA and thioTEPA, respectively, at concentrations of $50\,ng\,ml^{-1}$. The limit of sensitivity was $1–5\,ng\,ml^{-1}$ for both compounds. The elimination kinetics of thioTEPA were biexponential in 3 of 6 patients and the half-life of the initial phase was greater (range, 1.4–2.5 h) than in mice (mean ± s.e., 14.0 ± 0.4 min). The extent of binding of therapeutic concentrations of TEPA and thioTEPA to human plasma components under physiological conditions was determined by ultrafiltration and was <40%. It is unlikely, therefore, that competition for protein binding sites by concomitantly administered steroid will have any significant effect on plasma levels of the pharmacologically-active agents.

### Initial results with ovarian cancer antigen CA125

P.A. Canney[1], M. Moore[2], R.D. James[3] & P.M. Wilkinson

*[1]Department of Clinical Pharmacology, [2]Paterson Laboratories, [3]Department of Radiotherapy, Christie Hospital and Holt Radium Institute, Manchester, UK.*

CA125 has been measured in serum of patients using the method previously described by Bast *et al.,* 1983, *N.E.J.M.,* **309,** 883). Nineteen of 23 (83%) patients with epithelial ovarian carcinomas exhibited elevated CA125 levels ($>35\,U\,ml^{-1}$). Although considerable interpatient variation occurred, there was a positive association between the elevation of CA125 and estimated body burden of tumour. One patient thought to have had complete resection of early stage ovarian cancer had an elevated CA125 level at 16 days post-operatively; 3 further patients who had complete resections had a normal level when assayed at 22, 33 and 90 days post-operatively. Sequential measurements at an interval of one month were performed on 8 patients who had received chemotherapy. The half life of CA125 varied between 5 and 95 days; in 5 patients being between 5 and 10 days. An acute rise in response to chemotherapy, occuring within the first 5 days after treatment, was seen in 3/4 patients studied. Four out of five patients with uterine endometrial carcinoma and 2/2 patients with adenocarcinoma of the cervix had raised CA125 levels, 3/4 patients with large bowel carcinoma had raised CA125 levels. None of 19 in a control group consisting of patients in complete remission following chemotherapy for lymphoma, showed elevated CA125 levels (mean $10.9 \pm 4.4\,U\,ml^{-1}$). The lack of specificity with regard to major differential diagnoses for ovarian cancer renders the antigen unsuitable as a diagnostic or screening tool. However, the high true positive rate and this demonstration of short term variations in CA125 levels in response to chemotherapy indicate considerable promise for its use on a marker to monitor treatment. It may also be of value in the management of uterine carcinoma.

### A study of secretory component in breast cancer using monoclonal and polyclonal antisera

M.E. Gore, B.A. Gusterson, R.C. Coombes & R.A.J. McIlhinney

*Ludwig Institute for Cancer Research, Royal Marsden Hospital, Sutton, Surrey, UK.*

The mouse monoclonal antibody LICR-LON – LC28 binds to both secretory IgA and free secretory component having a 10-fold greater affinity for the former. This differential affinity can be exploited, using immunohistochemical methods, to study the distribution of secretory IgA in tissue sections. The antibody is species specific and does not bind to rat, sheep or bovine secretory IgA.

Immunohistochemical studies of tissue sections stained with LICR-LON-LC28, conventional antisera to human secretory component and human alpha chains, confirmed that LICR-LON-LC28 can be used to study the distribution of secretory IgA, except where free secretory component is present in very large quantities.

Five out of 22 breast carcinomas showed evidence of secretory component production. IgA was absent from these five tumours and was present only in the cytoplasm of plasma cells infiltrating the tumours. This suggests that secretory component is not usually produced by breast cancers but that when it is present it is unable to blind IgA.

### Oestrogen receptors and prognosis in disseminated breast cancer

R.A. McClelland[1], T.J. Powles[1], & R.C. Coombes[2]

[1]*Royal Marsden Hospital and* [2]*Ludwig Institute for Cancer Research, Sutton, Surrey, SM2 5PX, UK.*

The importance of oestrogen receptor (ER) presence in the prognosis of disseminated breast cancer was investigated by actuarial analysis of follow up data from 305 patients with advanced disease. All patients had ER content assessed on primary tumour material by the dextran-coated charcoal method (McGuire *et al.*, 1975, *In: Estrogen Receptors in Human Breast Cancer*. p.1).

Of the 305 patients, 278 were assessable for response to therapy by UICC criteria. Seventy five showed complete or partial tumour regression, 71 had their disease stabilised and 132 had progressive disease despite treatment.

ER positive patients (111) did significantly better ($P=0.025$) than ER negative patients (79) with respect to their length of survival from first relapse to death (ER +ve>15 femtomoles receptor $mg^{-1}$ cytosol protein).

The length of survival from primary diagnosis and the disease free interval from primary diagnosis to first relapse were found to be unrelated to receptor content of the primary tumour although there was a trend towards longer overall survival from presentation in the ER +ve group.

These findings echo similar results from work with early breast cancer (Stages I and II) (Stewart *et al.*, 1983, *Eur. J. Cancer Clin. Oncol.*, **19**, 1381) and suggest that the presence of ER significantly affects patients but not their disease free interval.

### Uptake and incorporation of CCRG 81010 (M & B 39565, NSC 353451) into TLX5 mouse lymphoma

A.C. Horgan & M.J. Tisdale

*CRC Experimental Chemotherapy Group, Department of Pharmacy, University of Aston, Birmingham, B4 7ET, UK.*

CCRG 81010 (8-carbamoyl-3-(2-chloroethyl) imidazo [5,1-d]-1,2,3,5-tetrazin-4-(3H)-one) is a new antitumour drug with a broad spectrum of activity against murine tumours and is at present entered into a Phase I clinical trial. Preliminary investigations into its mode of action indicate that activity may be exerted via 5-[3-(2-chloroethyl) triazen-1-yl] imidazole-4-carboxamide (MCTIC), a possible metabolite of CCRG 81010. (Horgan *et al.*, 1983, *Br. J. Cancer*, **48**, 132).

In this study, we hoped to define the uptake of drug into tumour cells and the intra-cellular fate of the label. Uptake experiments were performed with TLX5 lymphoma using [6-$^{14}$C-imidazole] CCRG 81010. The rate of uptake was rapid and temperature dependent (equilibrium within 1 min at 37°C and 6 min at 4°C), directly proportional to drug concentration and independent of metabolic inhibitors. A cell:medium distribution ratio of unity was achieved suggesting a passive diffusion process. No difference in uptake was seen between drug sensitive and resistant cells.

Radioactivity was found to be progressively accumulated into acid-insoluble material. Acid hydrolysis followed by HPLC separation of DNA/RNA components showed that the radioactivity was associated solely with adenine and guanine. Over a 24 h period about 80% of the CCRG 81010 in the medium was converted to 5-aminoimidazole-4-carboxamide (AIC). Non-radio-labelled AIC suppressed the incorporation of radioactivity into nucleic acids but had no effect on the initial rate of uptake of CCRG 81010. It was concluded that all the radioactivity in DNA and RNA was due to the salvage of AIC for *de novo* base synthesis.

### Anti-tumour activity of CCRG 81010 on a panel of transplantable tumours in MRI mice

J.A. Double & M.C. Bibby

*Clinical Oncology Unit, University of Bradford, Bradford BD7 1DP, UK.*

CCRG 81010 is a new anti-cancer drug with a broad spectrum of activity in experimental tumours

(Stevens *et al.*, *Br. J. Cancer*, **48,** 120). In this study this agent has been tested against four transplantable mouse adenocarcinomata of the colon lines (MAC 13, 16, 26 and 15A) of varying growth characteristics and histology and a newly developed mouse lymphoma BML1. These tumour lines are generally non-responsive with useful effects only seen close to maximum tolerated dose. Best responses are seen with the nitrosoureas and cyclophosphamide. MAC 15A and BML1 are assessed by survival duration, MAC 13 by comparison of the ratio of treated tumour weights over control tumour weights and MAC 16 and MAC 26 assessment of growth delays at relative tumour volume 5.

A 73% extension in median survival time was produced in MAC 15A and an 80% extension in BLM1. Ninety-nine percent tumour inhibition was seen in the solid line MAC 13 and a 6 day growth delay was seen in MAC 26. Mac 16 was unresponsive. The anti-tumour responses seen with CCRG 81010 in these tumours are very similar to those seen with MeCCNU, currently the most active agent in the MAC series. Of the 2 slower growing tumours MAC 16 is unresponsive and MAC 26 only poorly responsive to both agents at maximum tolerated dose. Reasons for these variations in response are currently under investigation.

In view of its exciting preclinical activity similar to that previously shown by the nitrosoureas, it is to be hoped that the same disappointing clinical outcome does not follow.

### Mechanism of resistance to methotrexate (MTX) in human tumour xenografts

S.J. Harland, A.H. Calvert, M. Jones, Z. Siddik & K.R. Harrap

*Institute of Cancer Research, Sutton, Surrey, UK.*

Six human tumour xenografts grown in nude mice were tested *in vivo* against MTX. Only one of them, an acute myeloid leukaemia grown as a solid tumour, was sensitive. Groups of xenograft-bearing mice were then given MTX 100 mg kg$^{-1}$ i.p. and the dihydrofolate reductase (DHFR) activity of the cytosol of the tumours was determined at intervals. This apparent enzyme activity rose between 1.7 and 12.0-fold in the resistant tumours, usually peaking at 3–4 days after treatment, but fell in the sensitive tumour to 0.3 of the pretreatment value. These findings concur with studies on patients with acute leukaemia (Bertino, *et al.*, 1977, *Cancer Treat. Rep.* **61,** 667). However, measurement of the total MTX level in the supernatants and determination of Ki's for the inhibition of DHFR by MTX allowed the total DHFR content to be calculated. This rose 1.4–1.8-fold in the sensitive tumour, a value in the same range as those for the resistant tumours which contained very little MTX. Studies of the kinetics of the affinity purified DHFR from a resistant melanoma line suggested the existence of 2-types of DHFR with Ki's for MYX of 2.2 and 295 pM. The Ki of DHFR from the sensitive leukaemia line was 1.1 pM with no evidence of a low affinity form. These studies suggest that intrinsic resistance of human tumours may be mediated by the presence of a form of DHFR which is poorly inhibited by MTX. Short term changes in total DHFR levels did not differentiate the sensitive tumour from the resistant ones. Transport of $^3$H MTX into tumour slices was increased by low temperatures and metabolic inhibitors – the reverse situation from that described for murine systems.

### Methotrexate may be neurotoxic by interference with tetrahydrobiopterin metabolism

J.A. Blair, C. Morar & S.B. Whitburn

*Chemistry Department, University of Aston, Birmingham B4 7ET, UK.*

Tetrahydrobiopterin (THB) is the coenzyme required for the formation within the CNS of the neurotransmitters dopamine, noradrenaline and serotonin. Methotrexate (MTX) inhibits dihydropteridine reductase (DHPR) ($K_i = 6 \times 10^{-5}$ M), the enzyme which maintains THB in its fully reduced form. Thus the neurotoxicity of MTX may be due to interference with THB metabolism in the CNS.

The levels of THB and its oxidation products 7,8-dihydrobiopterin (DHB) and biopterin (B) were measured in whole rat brain using HPLC, acid-iodine oxidation and alkaline-iodine oxidation. In adult male control rats (10) total biopterins were $222.4 \pm 45.8$ (mean $\pm$ s.d.) pmol g$^{-1}$ wet weight, of which 95.3% was THB, 2.8% was DHB and 1.9% was B. Four hours after an oral dose of MTX (1 mg kg$^{-1}$) (6 rats) total brain biopterins were increased to $351.9 \pm 134$ pmol g$^{-1}$ wet weight ($P < 0.01$) and the percentage distribution of THB, DHB and B was unchanged. With an oral dose of 100 mg kg$^{-1}$ (6 rats), after total brain biopterins were similar to controls ($188.6 \pm 34.3$ pmol g$^{-1}$ wet weight) but the distribution of the biopterins was significantly altered. Percent THB was 76.7 ($P < 0.01$ compared to control), DHB was 18.7 ($P < 0.025$) and %B was 4.6 ($P < 0.01$).

Large doses of MTX therefore affect THB metabolism and significantly lower the levels of the active coenzyme while increasing the amounts of its inactive oxidised derivatives.

### The effect of combined cisplatin and methotrexate on two human bladder cancer xenografts

J.H. Hay & W. Duncan

*University Department of Clinical Oncology, Western General Hospital, Edinburgh, UK.*

Cisplatin (CDDP) and Methotrexate (MTX) are two of the most active agents available to treat metastatic transitional cell carcinoma (TCC). A combination of the two could have useful clinical applications if their effects were additive or, preferably, synergistic. We have used two xenografts – WX38 a grade 3] [TCC and WX48 a grade 2] [TCC – growing in immune-deprived mice (Hay *et al.*, 1982, *Br. J. Cancer* **46**, 465) to study the effect of this combination. Specific Growth Delay (SGD) has been used to assess the response of the xenografts to treatment and the effect of combinations of the drugs has been assessed using isobolograms from which the effect of an additive combination may be predicted. A synergistic combination will show effects greater than predicted and vice versa. The results are shown in the table below:

| | *Cisplatin* | | |
|---|---|---|---|
| Dose in $mg\,kg^{-1}$ | 7 | 4 | 2 |
| WX38 SGD values | 1.5 | 0.9 | 0.1 |
| WX48 SGD values | 0.6 | 0.4 | 0.1 |
| | *Methotrexate* | | |
| Dose in $mg\,kg^{-1}$ | 70 | 40 | 20 |
| WX38 SGD values | 2.0 | 1.4 | 0.5 |
| WX48 SGD values | 0.5 | 0.4 | 0.3 |

| | | |
|---|---|---|
| WX38 | $3\,mg\,kg^{-1}$ | CDDP and $30\,mg\,kg^{-1}$ MTX SGD = 2.9 (predicted 1.5–2.0) |
| WX48 | $1\,mg\,kg^{-1}$ | CDDP and $40\,mg\,kg^{-1}$ MTX SGD = 0.7 (predicted 0.5) |
| WX48 | $2.5\,mg\,kg^{-1}$ | CDDP and $25\,mg\,kg^{-1}$ MTX SGD = 0.7 (predicted 0.5) |

Although the SGD values produced by the combinations are all greater than predicted for a purely additive effect, these differences do not reach statistical significance. It is concluded that the combination of Cisplatin and Methotrexate in these xenografts is at least additive and may be showing some synergism.

This work was supported by the Cancer Research Campign.

### Studies on the mechanism of CB3717 induced hepatotoxicity

D.R. Newell, D.L. Alison, A.L. Jackman, A.H. Calvert & K.R. Harrap

*Department of Biochemical Pharmacology, Institute of Cancer Research, Sutton, Surrey, UK.*

CB3717 (N-(4-(N-((2-amino-4-hydroxy-6-quinazolinyl)methyl)prop-2-ynylamino)benzoyl)-L-glutamic acid) is an antifolate inhibitor of thymidylate synthetase currently undergoing clinical evaluation at a number of centres in the UK. Hepatotoxicity is the most prevalent side-effect observed with CB3717; 50% of patients develop elevations of plasma alanine transaminase (ALT) to over twice the upper limit of normal. In mice CB3717 also produced elevations of plasma ALT which were maximal 6 h after a single i.p. dose, returning to normal by 24 h. After $100\,mg\,kg^{-1}$ CB3717, plasma ALT levels of 2–30 × control were observed whilst at $300\,mg\,kg^{-1}$ ALT elevations of 28–50 fold were produced. The administration of thymidine ($2 \times 500\,mg\,kg^{-1}$), 5-methyltetrahydrofolate (1 or $3 \times 10\,mg\,kg^{-1}$) or folinic acid (1 or $3 \times 10\,mg\,kg^{-1}$) did not reduce CB3717 hepatotoxicity in mice. As thymidine and folinic acid can abolish the antitumour activity of CB3717 it is unlikely that hepatotoxicity is mediated through inhibition of liver thymidylate synthesis. Although CB3717 can produce a 40% depletion of liver total non-protein thiols the administration of N-acetyl cysteine ($500\,mg\,kg^{-1}$) did not reduce CB3717 hepatotoxicity. Studies with structurally related quinazolines showed that equimolar doses of the $N^{10}$-ethyl and $N^{10}$-unsubstituted analogues produced no hepatotoxicity. However, pharmacokinetic studies demonstrated that plasma quinazoline levels following the two analogues were considerably lower (< 1/10th) than those observed after CB3717. This suggests that the liver damage produced by CB3717 may limit its hepatic clearance. Furthermore, CB3717 hepatotoxicity is apparently related to the $N^{10}$-prop-2-ynyl substituent.

### A new quinazoline antifolate drug CB3717 which inhibits the growth of human liver cancer cells in athymic mice, cell culture and patients

N. Curtin, A.L. Harris[1], O.F.W. James & M.F. Bassendine

*Departments of Medicine and Clinical Oncology[1], University of Newcastle upon Tyne, UK.*

The prognosis of patients with primary liver cancer (PLC) is poor, despite the currently available chemotherapy. Since thymidylate synthetase activity is high in PLC cells, we have studied a new quinazoline antifolate drug, CB3717, which inhibits this target enzyme (Jones *et al.*, 1981, *Eur. J. Cancer,* **17,** 11). The effect of CB3717 on the growth of human PLC cells, PLC/PRF/5, was studied in tissue culture and as a solid tumour in athymic mice.

Tumour growth was measured by serial serum AFP levels, a reflection of viable tumour mass (Bassendine *et al.*, 1983, *Clin. Sci.*, **64,** 643), and by direct volume estimation. Measurements from mice bearing visible tumours treated for 5 days with either 125 mg or 200 mg CB3717 $Kg^{-1} day^{-1}$ i.p. or two 5-day courses of 200 mg CB3717 $Kg^{-1} day^{-1}$ i.p. separated by 9 days were compared with those obtained from control tumour-bearing mice.

Serum AFP concentrations were significantly reduced compared to controls in low dose ($P<0.05$) and high dose ($P<0.02$) groups, tumour volume was significantly reduced in the high dose ($P<0.05$) group only. A second period of drug administration further reduced tumour growth, as measured by AFP levels. *In vitro* experiments give an ID50 of 1–5 $\mu$M, which is in the range achieved by the dose of 300 mg $m^{-2}$ given to patients. Our study shows that CB3717 is a potent inhibitor of human PLC growth *in vivo* and *in vitro.* Unlike methotrexate it preserves *de novo* purine synthesis, leading to low toxicity and its therapeutic efficiency is reflected in an early clinical evaluation.

### Studies on the pharmacokinetics and metabolism of some dimethylphenyltriazenes

C.J. Rutty, R.B. Vincent, G. Abel, P.M. Goddard & K.R. Harrap

*Department of Biochemical Pharmacology, Institute of Cancer Research, Belmont, Sutton, Surrey, UK.*

A number of dimethylphenyltriazenes have been synthesised in a search for a suitable clinical alternative to DTIC, which have demonstrated activity in experimental tumour systems. It is believed that, like DTIC, such compounds require metabolic activation, i.e. oxidative N-demethylation resulting in the formation of an unstable monomethyltriazene metabolite which on decomposition yields a species capable of methylating cellular macromolecules. 1-p-Carboxyamidophenyl-3,3-dimethyltriazene (CB 10–286) showed marked activity versus a mouse PC6 plasmacytoma (T.I. = 56), and is very rapidly metabolised in the mouse (plasma $t\frac{1}{2}\beta = 3.7 \pm 0.25$ min). This resulted in high plasma levels ($103.5 \pm 3.2\,\mu M$) of 1-p-carboxyamidophenyl-3-monomethyltriazene (CB 10-347), the putative active metabolite (AUC = 1159.2 $\mu$M × min). These levels are comparable to those produced by direct administration of CB 10-347 to mice (AUC = 1184.2 $\mu$M × min) which gave similar inhibition of tumour growth. In the rat plasma clearance of CB 10-286 was slower ($t\frac{1}{2}\beta = 16.9 \pm 0.86$ min), and the peak levels of CB 10-347 were much lower ($26.4 \pm 4.2\,\mu M$), although still some 13 times greater than the levels of the corresponding monomethyl metabolite produced by DTIC metabolism in the rat. Despite significant activity of 1-p-carboxyphenyl-3,3-dimethyltriazene (CB 10-277) against the PC6 tumour (T.I. = 21.5) this compound failed to undergo N-demethylation *in vitro* in contrast to the carboxyamido derivative. The plasma $t\frac{1}{2}\beta$ for this compound in mice ($93.1 \pm 8.9$ min) is also considerably longer than for CB 10-286, and there is no apparent formation of a monomethyl metabolite *in vivo*. Thus the antitumour activity of this drug does not appear to be dependent on oxidative metabolism.

### Derivatives of hydroxymethyltriazenes: Potential prodrugs of the active metabolite of aryldimethyltriazenes

K. Vaughan, R.J. LaFrance, C.M. Hemens, Y. Tang & D.C. Chubb[1]

*Department of Chemistry, Saint Mary's University, Halifax, N.S., Canada, and [1]CRC Experimental Chemotherapy Group, Department of Pharmacy, University of Aston, UK.*

Recent metabolic studies (Rutty *et al.*, 1983, *Brit. J. Cancer,* **48,** 140) suggest that the poor clinical activity of DTIC is due in part to the low level of oxidative metabolism of the drug in man. New α-substituted triazenes are being investigated for anti-tumour properties as potential prodrugs for the oxidative metabolites of DTIC and other dimethyltriazenes. Hydroxymethyltriazenes, ArN = N-$NRCH_2OH$, are synthesised by coupling an aryldiazonium ion to the carbinolamine, $RNHCH_2OH$, generated *in situ* from formaldehyde and the alkylamine. Hydroxymethyltriazenes react with acetic anhydride in pyridine to afford the acetoxymethyltriazenes, ArN = N-$NRCH_2OAc$, and with benzoyl chloride in pyridine to yield the benzoates, ArN = N-NR.$CH_2OCOPh$. These derivatives are suitable prodrugs since they should not require metabolic oxidation to exert a cytotoxic effect. The acetates and benzoates react with methanol to give the methoxymethyltriazenes, ArN = N-$NRCH_2OMe$, which may be suitable prodrugs through O-demethylation. The antitumour activity of a hydroxymethyl-, acetoxymethyl-, and methoxymethyltriazene has been compared with the activity of the corresponding dimethyl- and monomethyltriazenes (see **Table**).

| *Compound* | *Optimum Dose mg Kg*$^{-1}$ | *%T/C*[1] |
|---|---|---|
| ArN = N-$NMe_2$ | 80 | 53 |
| ArN = N-NHMe | 5 | 87[2] |
| ArN = N-NMe.$CH_2OH$ | 5 | 120 |
| ArN = N-NMe.$CH_2OAc$ | 40 | 56 |
| ArN = N-NMe.$CH_2OMe$ | 40 | 68 |
| ArN = N-NMe.$Ch_2O$-COPh | 20 | 31 |

(Ar = p-$MeO_2C.C_6H_4$-)

1. $2 \times 10^5$ TLX5 lymphoma cells S.C. day 0 into CBA mice in groups of 5. Drugs administered days 3 to 7.
2. A. Gescher, 1981 *et al., Biochem. Pharmacol.*, **30**, 89.

### Nitroreduction of the chemosensitizer and radio-sensitizer benznidazole *in vivo* and *in vitro*

M.I. Walton & P. Workman

*MRC Clinical Oncology and Radiotherapeutics Unit, Hills Road, Cambridge CB2 2QH, UK.*

Benznidazole is a lipophilic analogue of misonidazole currently undergoing phase I clinical trial as a chemosensitizer, in combination with the nitrosourea CCNU. Reductive metabolism of nitroimidazoles to their amine derivatives has been implicated in the mechanism of cytotoxicity and chemosensitization, possibly through the production of chemically reactive intermediates. To study benznidazole nitroreduction we have developed a reverse-phase paired -ion HPLC technique for the analysis of the amine, employing low wavelength UV detection and have subsequently investigated its production and distribution in mice, 3 h after a single i.p. dose of 650 mg kg$^{-1}$ benznidazole. This dose produces plasma benznidazole concentrations of 120–130 $\mu$g ml$^{-1}$ with tumour/plasma ratios of 80–100% in C3H mice bearing RIF-1 and KHT tumours, and BALB/c mice bearing EMT6 tumours. Plasma amine concentrations were very low (0.3-0.7 $\mu$g ml$^{-1}$) with similarly low amounts present in the 2 C3H mouse tumours (0.3–1.4 $\mu$g g$^{-1}$). Higher concentrations were detected in EMT6 tumours (4.1 $\mu$g g$^{-1}$ $\pm$ 0.33 SE, n = 16) suggesting that this tumour may be more hypoxic or have greater nitroreductase activity than RIF-1 and KHT tumours. Livers from these C3H mice contained very high levels of amine (51 $\mu$g g$^{-1}$ $\pm$ 2.5, n = 11) implicating this tissue as a major site of nitroreduction. Both strains of mice excreted ~3% of administered benznidazole as the amine in the 24 h urine, with a further 4% as the parent compound. We have also demonstrated *in vitro* nitroreduction of benznidazole using whole liver homogenates. The reaction is inhibited by oxygen and has an absolute requirement for reduced nicotinamide adenine dinucleotides, particularly NADPH. In summary, nitroreduction of benznidazole has been shown to occur in mouse tissue *in vivo* and *in vitro*, and its clinical significance is under investigation.

### Misonidazole enhancement of the action of melphalan in a human breast tumour xenograft

A.M.L. Coombs & J.L. Moore

*Department of Radiobiology, Velindre Hospital, Cardiff, UK.*

A human breast tumour xenograft, HX99 (donated by A.C. Jones, Royal Marsden Hospital, London), implanted in immune suppressed female CBA mice (Steel, 1978 *et al., Br. J. Cancer*, **37**, 224), has been used to study the *in vivo* chemosensitization of melphalan (MEL) by misonidazole (MISO). Mice bearing tumours of approximately 8 mm diameter were injected i.p. with drugs delivered in 0.01 ml$^{-1}$ g. MEL was used in doses of up to and including the $LD_{10}$ value (14 mg$^{-1}$ kg). MISO was administered as total doses of 1 mg$^{-1}$ g, 0.5 mg$^{-1}$ g, 0.25 mg$^{-1}$ g, and 0.1 mg$^{-1}$ g. Tumours were excised after 20 h and cell kill determined by a soft agar assay (Courtenay & Mills, 1978, *Br. J. Cancer*, **37**, 261). MEL at 14 mg$^{-1}$ kg gave a 40% cell kill and MISO alone at 1 mg$^{-1}$ g gave no significant cell kill. However, when combined chemosensitization was seen, for example, with total doses of $1\,mg^{-1}\,g$ and $0.5\,mg^{-1}g$, a cell kill of greater than 96% was produced.

### Inhibition of liver microsomal metabolism of CCNU by nitroimidazoles – correlation with chemosensitisation activity

F.Y.F. Lee & P. Workman

*MRC Clinical Oncology and Radiotherapeutics Units, Cambridge, UK.*

We previously showed that misonidazole (MISO) reduced the clearance of CCNU in mice resulting in a selective increase in tumour CCNU concentration, and we proposed this to be the major mechanism of chemosensitisation and therapeutic gain (Lee & Workman, 1983, *Br. J. Cancer,* **47,** 659). Since both MISO and CCNU undergo extensive metabolism in the liver, the reduced clearance of CCNU is likely to be due to the effect of MISO on hepatic CCNU metabolism. We have therefore studied the effect of a range of nitroimidazoles of varying chemosensitisation activity (Workman & Twentyman, 1982, *Br. J. Cancer,* **46,** 249) on the metabolism of CCNU by mouse liver microsomes. Microsomes were prepared by standard subcellular fractionation. CCNU and its metabolites were separated and individually quantitated using a reversed-phase HPLC method. In the presence of $O_2$, glucose-6-phosphate, glucose-6-phosphate dehydrogenase and NADP, CCNU is metabolised to at least 5 different ring-monohydroxylated active derivatives. Values of $K_m$ and $V_{max}$ for the formation of the major metabolite cis-4-hydroxy-CCNU were 0.028 mM and 2.08 $nmoles\,min^{-1}\,mg^{-1}$ microsomal protein respectively. MISO inhibits the rate of formation of the metabolites with an $ID_{50}$ of 6.4 mM. Values of $ID_{50}$ for the various nitroimidazoles were in the order: benznidazole $<$ Ro07-1902 $<$ MISO $<$ Ro05-9963 $<$ SR-2508. At concentrations similar to the achieved plasma concentrations in mice given 2.5 $mmoles\,kg^{-1}$, the extent of inhibition of CCNU metabolism was 80% for benznidazole, 65% for Ro07-1902, 33% for MISO, and 0% for Ro05-9963 and SR-2508. Therefore these data show that the order of effectiveness of nitroimidazoles in inhibiting microsomal CCNU metabolism directly correlates with the order of efficacy for enhancement of tumour response and provide further evidence for the pharmacokinetic mechanism of chemosensitisation.

### Effect of preheat on intracellular adriamycin concentration and resulting surviving fraction of EMT6 cells *in vitro*

S.H. Chambers, N.M. Bleehen, & K. Wright

*MRC Clinical Oncology and Radiotherapeutics Unit, Hills Road, Cambridge CB2 2QH, UK.*

We have investigated the response of exponentially growing EMT6 tumour cells, *in vitro,* to treatment with hyperthermia (1 h at 43°C) prior to treatment with Adriamycin (ADM) (0–20 $\mu g\,ml^{-1}$ for 1 h at 37°C). Relative intracellular ADM concentration was measured by flow cytometry (FCM) and compared with survival data (clonogenic assay). FCM demonstrated that control cells accumulated twice as much ADM as preheated cells. However, FCM measurements of ADM in cells permeabolised after heat treatment but before drug administration indicated that hyperthermia did not significantly alter the amount of ADM that could be accumulated once the membrane barrier had been removed. Survival data showed that preheated cells had a higher surviving fraction than control cells, 0.3 at 3 $\mu g\,ml^{-1}$ compared to 0.007 for unheated cells, as predicted by FCM. However, comparing surviving fraction data with intracellular drug measurement revealed that preheated cells were less sensitive to a given internal ADM concentration (at an FCM measurement of 100 fluorescence units preheated cells have a surviving fraction of 0.1 compared to 0.01 for non-heated cells.) This suggests that the initial observed difference in surviving fraction is not due simply to a difference in accumulation of ADM but also a change in the intrinsic sensitivity of cells that have been subject to preheating at 43°C. We conclude therefore, in EMT6 cells, preheating for 1 h at 43°C protects the cell from ADM by reducing the amount of drug entering the cell, apparently by altering membrane permeability, and also protects the cell by reducing its sensitivity to the drug that does pass through the membrane.

### The response of V79 and human tumour multicellular spheroids to adriamycin and in MAMSA: Cell survival, growth delay and ultrastructural studies

J.M. Walling[1], S.K. Townsend[1], C.M.L. West[2] & I.J. Stratford[1]

[1]*MRC Radiobiology Unit, Harwell, Didcot, Oxon, U.K.,* [2]*University of Rochester Cancer Center, 601 Elmwood Avenue, Rochester, USA.*

Three-dimensional spheroids can be regarded as good *in vitro* models for solid tumours. It has been suggested that cell to cell contact can be an important mediator of the response of spheroids to damage caused by radiation and cytotoxic drugs. We have been assessing the role of contact effects in the radiation and drug response of V79 and human tumour spheroids (ME/MAR – small cell lung carcinoma cell line and HX 117 – melanoma xenograft). This report describes a comparative study of the ultrastructural and cytotoxic effects of the intercalating agents, adriamycin and mAMSA. Electron microscopic examination shows that V79 spheroids are characterized by the possession of desmosome type junctions as well as tight junctions, HX 117 have many fewer desmosome type junctions, and ME/MAR spheroids lack any recognizable junctions whatsoever. Both adriamycin and mAMSA cause degeneration of cell membranes within spheroids at doses that do not cause any recognizable nuclear aberrations. The membrane effects are most marked near the outside of the spheroid and are not apparent towards the centre, the region containing the quiescent cells and hypoxic cells.

**Relationship between bone marrow glutathione and glutathione S-transferase levels and drug priming**

D.J. Adams, J. Carmichael, J.F. Smyth & C.R. Wolf.

*Imperial Cancer Research Fund, Medical Oncology Unit, Western General Hospital, Edinburgh, UK.*

Drug priming is a phenomenon where a small "priming" dose of a cytotoxic compound will protect patients from the acute toxicity of a much higher dose of the same or a different compound. In the present study we have investigated the relationship between priming as protection against cyclophosphamide (CP)-induced-myelotoxicity, and reduced glutathione (GSH) and glutathione-S-transferase (G-S-T) levels in the mouse bone marrow.

CBA mice survived an otherwise lethal dose of CP ($350\,mg\,kg^{-1}$) when "primed" with an injection of $75\,mg\,kg^{-1}$ CP five days prior to the lethal dose. Our data demonstrate that a lethal dose of CP caused a dramatic depletion of GSH in the bone marrow and that the extent of this depletion was limited by a priming dose of CP. Although a priming dose of CP caused an initial depletion of GSH, the GSH content of the marrow in primed animals was 180–290% that of controls at day 5. The time course of intracellular glutathione levels mirrored the susceptibility of the mice to a lethal dose of cyclophosphamide. This was also the case for glutathione S-transferases which on day 5 were dramatically increased over control values (252% control).

We postulate that survival of primed animals injected with an otherwise lethal dose of CP five days after priming is due, at least in part to the high levels of GSH and G-S-T detected in the bone marrow. That is, that the "overshoot" of GSH and G-S-T allows for the removal of the fatally myelotoxic products of cyclophosphamide metabolism through conjugation with GSH.

**Molecular events in the formation of the tumour marker, gamma glutamyl transpeptidase**

C.A. Power*, J.L. Simpson & M.M. Manson

*MRC Toxicology Unit, Woodmansterne Road, Carshalton, Surrey, UK.*

Gamma glutamyl transpeptidase (GGT), a membrane bound glycoprotein consisting of 2 heterologous subunits, exists as several isosymes, varying in their degree of glycosylation. Enzyme levels are high in fetal rat liver, while the very low activity in adult liver is confined mainly to bile duct epithelia. However, exposure to a wide range of carcinogens leads to the formation of preneoplastic foci of hepatocytes, many of which are GGT positive.

To investigate the role of GGT in carcinogenesis we are studying the molecular events in the formation of the enzyme. Total RNA has been isolated by guanidinium isothiocyanate extraction from rat tissues rich in GGT, i.e. kidney, aflatoxin $B_1$ ($AFB_1$) fed-liver and a tumour cell line derived from such a liver. *In vitro* translation of RNA using the rabbit reticulocyte lysate system and immunoprecipitation of specific translation products using anti-kidney GGT polyclonal antibody** and protein A have been used to identify the non-glycosylated pre-protein form of the enzyme. RNA preparations have been enriched for the mRNA of interest by fractionation on 5–20% sucrose gradients and by oligo-dT-cellulose chromatography.

Such enriched mRNA preparations have been used to synthesize cDNA, which in turn can be used as a probe to investigate the effect of carcinogens on mRNA populations and transcription of the gene(s) for GGT.

---

*Funded by National Foundation for Cancer Research.
**Kindly provided by Dr. S. Murphy, Open University.

### Monoclonal antibody to show heterogeneity in normal and carcinogen fed rat liver

M.M. Manson & J.A. Green

*MRC Toxicology Unit, Woodmansterne Road, Carshalton, Surrey, UK.*

Monoclonal antibodies have been raised against a γ-glutamyl transpeptidase (GGT) positive rat liver tumour cell line (JB1) from an aflatoxin ($AFB_1$) fed F344 rat. BALB/c mice were immunized with JB1 cells and spleen cells fused with NSO myeloma cells. Hybridoma supernatants were screened against JB1 cells by ELISA, and positive cultures further screened by immunohistochemistry on cold acetone-fixed sections of control and $AFB_1$-fed rat liver using anti-mouse γ chain alkaline phosphatase immunoconjugate. Twenty six cultures were selected and one cloned twice in soft agar. This hybridoma MRC TU/J1 secretes IgG1 immunoglobulin which has been used to follow changes in antigen distribution in response to $AFB_1$ administration.

In control neonatal and young male rat liver the membrane bound antigen is evenly distributed throughout hepatocytes. Older control animals (>12 weeks) show a zonal distribution with stronger stain in periportal regions. Bile ducts are stained in all sections. The zonal staining becomes stronger during the first weeks of $AFB_1$ feeding (4 ppm) when GGT-+ve biliary hyperplasia occurs. From 4 wks, when GGT-+ve foci appear, the antigen distribution becomes increasingly heterogeneous with some foci heavily stained, other apparently negative. Areas of biliary hyperplasia are darkly stained. In serial sections stained for GGT, foci positive for this enzyme are often negative or heterogeneous with respect to antigen distribution. This monoclonal antibody demonstrates, more clearly than GGT histochemistry, the heterogeneity among hepatocytes in control and particularly carcinogen treated liver.

### Changes in growth factor receptor function during chemical hepatocarcinogenesis

B.I. Carr[1], A. Lev-Ran, Z. Yosefsberg, G. Barseghian & D. Hwang

[1]*Departments of Medical Oncology and Endocrinology and Diabetes, City of Hope Natl. Medical Center, Duarte, California, 91010, USA.*

Insulin and epidermal growth factor (EGF) are known hepatic mitogens. We have measured the binding of insulin and EGF to liver during chemical carcinogenesis to identify growth regulatory changes that may be of importance. Young adult male F344 rats were fed either a basal diet or one supplemented with 2-acetylaminofluorene 0.02%(AAF). After sacrifice, livers were perfused, excised and microsomal and Golgi membranes were prepared. Binding was performed using $^{125}$I-insulin or -EGF *in vitro* at +4°C. A decrease in the binding to the Golgi membranes was found for both EGF and insulin after AAF feeding of 2–85 days. The % specific binding per 0.1 mg protein for EGF was 15.2 (control), 9.4 (2d AAF), 0.8 (21d AAF). For insulin: 10 (control), 6 (2d AAF), 1.5 (21d AAF). A similar decrease was noted for microsomes, but was slower and less pronounced. Competition studies with both Golgi and microsomal membranes showed an AAF-induced decrease in the number but not affinity of both EGF and insulin receptors. To determine whether this decrease was a direct toxic effect, rats were fed AAF for 3 mo. and returned to a basal diet for a further 2 mo. The livers of such rats off all carcinogen also demonstrated a lower binding to EGF and insulin. Direct incubation of normal membranes *in vitro* with AAF or its active metabolites hydroxy-AAF or acetoxy-AAF produced no significant decrease in the binding for EGF and insulin. Thus, carcinogen feeding induced a rapid, apparently stable decrease in the number of insulin and EGF receptors by a mechanism other than toxic damage. This could be due to altered receptor structure, synthesis, or breakdown; or production of a factor which binds to the insulin and EGF receptors.

### Tumorigenicity and cytotoxicity of 5-azacytidine and analogues

B.I. Carr[1] A. Riggs, J.G. Reilly & S. Dickman[1]

[1]*Department of Medical Oncology and Division of Biology, City of Hope Natl. Medical Center, Duarte, California, 91010, USA.*

5-azacytidine (Aza-C) is known to inhibit methylation in newly synthesized DNA and to induce gene activation. It has been used in the experimental treatment of acute leukaemia, thalassaemia and sickle cell anaemia. This report shows that it is also a carcinogen. Young adult male F344 rats were given Aza-C 2.5–10 mg $kg^{-1}$ i.p. in saline twice weekly for 9 months and examined 9 months later. About 30% of the rats died before the end of the experiment and could not be evaluated. Of the survivors, the number of rats with tumours/number of rats examined were 17/20. Controls 0/12. The tumour types were Leydig, Sertoli and interstitial cell testis (10); invasive squamous cell skin (3); adeno-carcinoma of lung (1) and reticuloendotheliosis (1). A follow-up experiment using Zza-C analogues has been initiated. The cytotoxicity of Aza-C and analogues (all at $10^{-4}$M) was examined using a 24h viability assay with normal rat hepatocytes. Results (% survival/controls) were (av. of 6 expts. = cytosine 85; cytidine 77; 5-fluorocytosine 69; 5-fluorocytidine 47; 5-fluoro-2-deoxycytidine 62; 5-azacytidine 32; 5-aza-2-deoxycytidine 63; 5,6-dihydro-5-azacytidine 50; 6-azacytidine 67; pseudoisocytidine 70. Tetra-hydrouridine, a deamination inhibitor, was not toxic and did not increase the toxicity of any of the above analogs at $10^{-6}-10^{-2}$M. Thus, Aza-C is a carcinogen in the rat. Aza-C, 5-fluorocytidine and 5,6-dihydro-Aza-C are toxic *in vitro*. The deoxyribose analogues are less toxic than the ribose analogues, suggesting that cytotoxicity may not be mediated solely through DNA. In current *in vivo* expts. a similar toxicity is observed as weight loss with Aza-C and fluorocytidine but not with equi-molecular amounts of their deoxy analogues.

**Radiation-induced activation of 4-NQO and its chemical consequences for DNA**

C.L. Greenstock

*Paterson Laboratories, Christie Hospital & Holt Radium Institute, Manchester M20 9BX, UK.*

Many chemicals undergo metabolic activation to ultimate carcinogens. The subsequent interactions of these reactive intermediates with critical cellular targets are important precursors to malignant cell transformation, and studies of the kinetic and molecular processes may help in understanding the overall mechanisms of carcinogenesis.

Irradiation of calf thymus DNA in the presence of 4-NQO leads to the formation of trichloroacetic acid precipitable, presumably covalent DNA-4-NQO, complexes. When 4-NQO is present in excess, the nucleophilic radiolysis species $e^-_{aq}$ and $O_2^{\cdot -}$. are principally responsible for adduct formation, suggesting the radical anion 4-NQO$^{\cdot -}$ and other reductive metabolites are toxic. The 4-NQO also contributes to the enhancement of radiolytic destruction of pyrimidine and purine nucleotides in DNA, as monitored by HPLC following enzyme hydrolysis. Pulse radiolysis studies show that 4-NQO$^{\cdot -}$ reacts with nucleic acid, fatty acid and amino acid moieties, by free radical redox reactions which may be involved in the carcinogenic and cytotoxic effects of 4-NQO.

**Metabolic activation of chrysene: Comparative studies with rodent and human skin**

A. Weston, R.M. Hodgson, A. Hewer & P.L. Grover

*Institute of Cancer Research: Royal Cancer Hospital, Fulham Road, London SW3 6JB, UK.*

In an attempt to assess the sensitivity of human skin to polycyclic hydrocarbon carcinogenesis, the metabolism and activation of hydrocarbons in susceptible and resistant rodent skin *in vivo* and *in vitro* is being examined and compared with that occurring in human skin in short-term organ culture. Chrysene, an environmental contaminant, is weakly carcinogenic to mouse skin and is converted by human and mouse skin in organ culture and by mouse skin *in vivo* to chrysene-1,2-, 3,4- and 5,6-dihydrodiols: A chrysene-triol that is believed to be 9-hydroxychrysene 1,2-diol was also found in some experiments. Chrysene-1,2-diol is a precursor of both the "bay-region" diol-epoxide, chrysene-1,2-diol 3,4-oxide, and a triol-epoxide in mouse skin; chrysene-1,2-diol is also more active than the parent hydrocarbon as a tumour initiator in this mouse tissue. The stereochemistry of the 1,2-diol and of the other diols that are formed as metabolites of chrysene by mouse skin was investigated by normal phase hplc using columns that contained an optically-active stationary phase and it was found that (−)-enantiomers were formed almost exclusively. When chrysene-treated mouse skin was maintained in short-term organ culture, dihydrodiols were released into the medium, however, the enantiomeric purity of the 1,2-diol did not vary as it accumulated over a 40h incubation period. Levels of the chrysene-triol appeared to reach equilibrium after 20–24h. The formation and the enantiomeric purity of the chrysene-triol that is formed by metabolism from optically-pure samples of the chrysene-1,2-diol is being examined together with the hydrocarbon-deoxyribonucleoside adducts that are formed in human skin that has been treated in short-term organ culture with chrysene.

### Frequency of exposure is more important than total dose in determining carcinogenic response

R.M Hicks, J.A. Turton, K. Nandra, J. Gwynne, M. Pedrick & E. Chrysostomou

*School of Pathology, Middlesex Hospital Medical School, London W1P 7LD, UK.*

It is well known that in many tissues cancer incidence is directly proportional to the dose of carcinogen. This dose-response relationship is substantially modified by the frequency of dosing.

F344 rats were given 150, 300 or 600 mg of the bladder carcinogen N-butyl-N-(4-hydroxybutyl) nitrosamine (BBN) in 3, 6, 9 or 12 weekly aliquots. The incidence of bladder tumours and hyperplasias was assessed at 1, 13, 26 and 52 weeks post-dosing. With equal numbers of aliquots there was a clear dose-related response, but for each total dose more and smaller aliquots produced a greater response than fewer larger aliquots. The frequency with which the tissue was exposed to BBN was more important than the total dose; for example, tumour incidence after 600 mg split into 3 doses was lower than after 300 mg split into 6 or 150 mg split onto 12 aliquots.

These results emphasise the potential danger of multiple low dose exposure to carcinogens when attempting to assess risk factors. The results also indicated that the urothelial hyperplasias were precursors of the tumours.

### Binding of benzo(a)pyrene to regions of DNA with different concentrations of active and inactive genes

N.M. Mironov, K. Pal & P.L. Grover

*Chester Beatty Laboratories, Institute of Cancer Research: Royal Cancer Hospital, Fulham Road, London SW3 6JB, UK.*

The binding of benzo(a)pyrene (BP) to different regions of DNA was investigated in nuclei prepared from rat liver or hamster embryo cells. After digestion of DNA in the nuclei by either endogenous nuclease activity or restriction endonuclease HaeIII, chromatin was fractionated by suspending the nuclei in low ionic strength TM buffer (10 mM Tris-HC1, pH 7.6 containing 0.2 mM $MgCl_2$): this treatment resulted in the solubilization of about 60% of the chromatin. After centrifugation, the insoluble material was resuspended in TM buffer containing 2M NaCl, which solubilized most of the remaining DNA and left 0.5-5% of the nuclear DNA as insoluble, matrix-bound material. Concentrations of active albumin and inactive globin genes in fractions from rat-liver nuclei were determined by dot-blot hybridization. The ratio of albumin to globin genes in the matrix-bound fraction was 50–100 fold higher than in the low ionic strength soluble fraction. When rat-liver nuclei were incubated with $^3$H-benzo(a)pyrene (BP) before fractionation, the concentration of BP bound to DNA was 3-fold higher in the fraction enriched in active genes. Using hamster embryo cells, we found that the concentration of adducts was 16-fold higher in matrix-bound DNA than in DNA soluble in low ionic strength buffer and appeared to persist longer. The results suggest that active DNA sequences may be some 100–1000 fold more extensively modified by BP metabolites than inactive sequences.

### A preliminary report on the presence of reverse transcriptase in canine milk

T.D. Littlewood, F.M. Tomley & L.N. Owen

*University of Cambridge, Department of Genetics, Downing Street, Cambridge and Department of Clinical Veterinary Medicine, Madingley Road, Cambridge, UK.*

The epidemiology of several mammalian retroviruses has been studied and there is evidence of milk-borne transmission. The possibility of such a route of infection for a canine virus was investigated in a population of eighty clinically normal Beagle bitches. Significant poly (rC):oligo(dG)-templated reverse transcriptase activity was detected in milk from seven animals following preparation with the gradient material Metrizamide (Sigma), which is known to reduce the influence of inhibitors present in the milk (Sanner, 1976, *Cancer Res.*, **36**, 405). This activity was abolished in the presence of untreated milk and was not due to deoxy nucleotidyl transferase. No activity was detected in the cellular fraction of these samples following short-term culture with IUdR, and no virus-like particles were seen on electron microscopic examination.

The activity present in milk from clinically normal dogs requires further characterization and is relevant to any survey of milk-borne transmission of virus in diseased animals.

### Promotion of cell phenotypes by interleukin 3: Evidence for different interaction mechanisms for basophil and lymphoid lineages

J.M. Garland[1] & R. Palacios[2]

[1]*Department of Immunology, Medical School, Manchester, UK.*, [2]*Basel Institute for Immunology, Basel, Switzerland*

Interleukin 3 is recognised for its ability to (i) induce 20*a* steroid dehydrogenase a T-cell associated enzyme in nu/nu spleen cells and (ii) promote cell lines *in vitro*. Studies have been done to determine the phenotypes of cells promoted by IL-3. They include most basophils, G/M-CFU and CFU-S. Recently, a cloned pre-B IL-3 dependent line has been isolated (Palacios, R. *et al.*, submitted 1983) and we have investigated whether the mechanism of dependency is the same as that for a cloned basophil line. We have found: (i) both lines respond to IL-3 withdrawal by rapidly reducing lactic acid production; (ii) the basophil line does not measurably absorb IL-3, but absorption is associated with the lymphoid line; (iii) the basophil line responds to exogenous ATP by maintaining proliferation whereas the lymphoid line does not and (iv) for the basophil line, exogenous ATP suppresses lactic acid production. We conclude firstly that in both lines IL-3 ultimately affects cell respiration, but only in the basophil line is exogenous ATP able to feedback regulate lactic acid, supporting a role for IL-3 in cell respiration; secondly, there are different mechanisms whereby IL-3 interacts with basophils and lymphoid precursors and thirdly that IL-3 may regulate normal poesis through different interaction mechanisms expressed by different lineages.

### Cell promotion by interleukin 3: Inferences for its involvement in a leukaemic process

J.M. Garland[1] B. Pleuvry[2] & N.J.F. Dodd[3]

[1]*Department of Immunology,* [2]*Department of Pharmacology, Medical School, Manchester and* [3]*Paterson Laboratories, Manchester, UK.*

Interleukin 3, a murine 28Kd glycoprotein derived from stimulated T-cells and a myelomonocytic leukaemic cell line WEHI-3b, promotes survival and proliferation of a number of *in vitro* cell lines. We have investigated the mechanism of action of IL-3 by measuring changes in cell metabolism in a cloned dependent line when it is withdrawn. We have found: (i) changes in cell membrane integrity as measured by ESR and trypan blue exclusion are late events; (ii) cells arrest preferentially in G2/M; (iii) as determined by $pO_2$ and lactic acid production, cell respiration in both WEHI and IL-3 dependent cells is mainly by anerobic glycolysis, which is the first parameter to be affected by IL-3 withdrawal; (iv) IL-3 does not appear to affect $Ca^{++}$ metabolism. These observations suggest that IL-3 promotes directly or indirectly cell respiration; for example by affecting perhaps glucose transport. IL-3 is found in avascular nodules of WEHI-3b cells growing in leukaemic mice. Garland *et al.*, 1983, *Br. J. Cancer* **48,** 247. We hypothesise that WEHI cells are leukaemic because they constitutively produce a factor which promotes anerobic respiration.

### Studies of the mechanism of enhancement of natural killer cell activity by a streptococcal preparation

S.E. Christmas & M. Moore

*Immunology Department, Paterson Laboratories, Christie Hospital & Holt Radium Institute, Manchester M20 9BX, UK.*

The streptococcal immunopotentiator OK432 has been found to enhance the natural killer (NK) activity of human peripheral blood mononuclear cells (PBMC) *in vitro* (Wahangi *et al.*, 1982, *JNCI*, **69,** 807). Clinical trials in patients with carcinomatous pleural effusions gave promising results possibly due to the enhancement of NK activity *in vivo* (Uchida & Micksche, 1982, *Int. J. Cancer* **31,** 1). The aims of the present experiments were to investigate the cellular requirements for the **activation of NK cells by OK432 and the mechanism** of its action. Depletion of monocytes to as low as 0.3% of PBMC using plastic and nylon wool adherence failed to prevent the enhancement of NK activity by OK432. Supernatants from OK432-treated unseparated and non-adherent PBMC were able to enhance the NK activity of fresh cells following overnight preincubation. Supernatants were assayed for interleukin-2 (IL-2) and interferon (IFN) activities and it was found that IL-2 production was not tightly linked to NK enhancing ability as in most experiments non-adherent cells produced little or no IL-2 while still producing significant amounts of NK enhancing activity. The presence of IFNs in supernatants was more tightly linked to NK enhancing capacity but there were indications that an additional factor(s) was involved as supernatants with equal IL-2, IFN-α and IFN-γ activities had differing NK enhancing abilities. An anti-IFN-α antiserum slightly decreased NK enhancing activity and overnight treatment at pH 2 followed by re-neutralisation almost completely abolished activity. If a factor(s) other than IFN-α and -γ and IL-2 is involved in the enhancement of NK activity by OK432 it appears to be labile at pH 2.

**Metastases and surface glycoproteins in relationship to tumour implantation site**

G.A. Turner, W.-S. Chan & A. Jackson

*University Department of Clinical Biochemistry and Metabolic Medicine, Royal Victoria Infirmary, Newcastle upon Tyne, UK.*

A previous study (Chan *et al.*, 1984, *Br. J. Cancer*, in press) of a lymphosarcoma and its liver metastases suggested that the site of tumour growth is important in affecting the surface expression of certain Wheat Germ Agglutinin (WGA) binding proteins. This finding has been further investigated by directly implanting tumour into different sites, examining the subsequent patterns of tumour spread, and analysing the composition of the proteins extracted from the local tumour by using electrophoresis and $^{125}$I-WGA labelling. The sites tested were kidney, liver, muscle, peritoneal cavity (p.c.), spleen, subcutaneous and lung/thorax. The tumour grew in all sites, but its metastatic ability and distribution varied. Extensive liver metastases along with lymph node enlargement were a common finding for all sites, except for p.c. and thorax where liver involvement occurred infrequently. Extra-hepatic metastases also varied, being virtually non-existent for liver and spleen, fairly frequent for kidney and muscle and occasionally found for the subcutaneous site. Site also affected the extent of WGA binding. Strong binding patterns were seen for tumour extracts from kidney, muscle and the subcutaneous site, i.e the sites that formed the most extra-hepatic metastases. These results emphasize the importance of site in influencing metastatic properties and suggest that site-modulated tumour surface composition may have a role to play in this process.

***In vitro* studies of fibroblasts derived from patients with carcinoma of the breast**

P. Durning, S. Schor & R.A. Sellwood

*Department of Medical Oncology, Christie Hospital & Holt Radium Institute, Wilmslow Road, Manchester, UK.*

The epithelial cells in a tumour are regarded by most authorities to be responsible for the malignant properties of a tumour. There is considerable evidence that stromal cells, in particular fibroblasts, influence the behaviour of epithelial cells. Fibroblasts derived from some tumours have been shown recently to exhibit some of the properties used to define the transformed phenotype *in vitro*.

Fibroblasts derived from 24 carcinomas of the breast were examined in several assays commonly used to define transformation *in vitro*. No abnormalities of morphology, growth, serum requirement or the ability to contract a gel of hydrated collagen were found. We have demonstrated that normal and transformed cells show a consistent difference in their ability to migrate into a collagen gel. Apparently normal fibroblasts from eight patients with carcinomas of the breast showed a normal pattern but 16 patients showed a transformed pattern of migration. The relationship between the transformed pattern of migration and clinical indices of prognosis such as the presence of lymph node metastasis was statistically significant at the 3% level.

Fibroblasts derived from apparently normal skin of each of these patients were examined and also showed the same patterns of migration.

The implications of this finding in relation to the systemic nature of malignancy will be discussed.

**Patterns of cross sensitivity in the responses of clonal subpopulations isolated from RIF-1 mouse sarcoma to selected nitrosoureas and nitrogen mustards**

J.G. Reeve, K.A. Wright & P. Workman

*MRC Clinical Oncology and Radiotherapeutics Unit, MRC Centre, Hills Road, Cambridge CB2 2QH, UK.*

Clonal subpopulations isolated from the RIF-1 mouse sarcoma differ markedly in their response to *in vitro* treatment with melphalan and CCNU. For melphalan treatment drug sensitivity is independent of the ploidy level of individual clones. For CCNU treatment a clear relationship exists between drug sensitivity and ploidy with tetraploid and octoploid clones being markedly more resistant to drug treatment than diploid clones. In the present study RIF-1 clones growing in log phase culture were treated for 1 h at 37°C with appropriate concentrations of nitrogen mustard, aniline mustard and chlorambucil, and with nitrosoureas BCNU, MeCCNU and chlorozotocin, in order to evaluate whether or not the different physiochemical and biological activities of these agents would affect the patterns of drug sensitivity described above. Irrespective of the different lipophilicities and transport mechanisms of the nitrogen mustards used in the present study, RIF-1 clones showed the same pattern of sensitivity when exposed to either melphalan, nitrogen mustard, aniline mustard or chlorambucil. Similarly RIF-1 clones when exposed to BCNU, MeCCNU and chlorozotocin showed the same pattern of sensitivity as that observed for CCNU treatment. These data suggest (a) that the variation in the sensitivity of RIF-1 clones to treatment by the nitrogen mustards is unlikely to reflect differences in either membrane permeability or in drug transport mechanisms within the cells (b) that the ploidy dependent nitrosourea responses shown by RIF-1 clones similarly do not reflect differences in membrane permeabilities or in cell sensitivities to relative alkylation versus carbamoylation. Furthermore, it is likely that a common mechanism may predominantly govern response to the different nitrosoureas, whereas a different common mechanism may govern response to the nitrogen mustards.

### Nitrogen mustard selectively inhibits rubidium influx into murine L1210 leukaemia cells

C. Wilcock, J.A. Hickman & S.B. Chahwala

*CRC Experimental Chemotherapy Group, Department of Pharmacy, University of Aston, Birmingham B5 7ET, UK.*

The interaction of antineoplastic drugs with the plasma membrane may contribute or be causative to their cytotoxicity. It has been shown that nitrogen mustard (HN2) inhibited the net accumulation of $^{86}Rb^+$ (a potassium congener) into mouse plasmacytoma cells (Baxter *et al.*, 1982, *Biochem. Pharmacol.*, **31**, 1773). This inhibition was suggested to result from the inactivation of the enzyme $Na^+K^+$ATPase, which plays an important role in the control of cell proliferation. In order to define more fully these observations on net $^{86}Rb^+$ accumulation, the effects of HN2 ($10^{-5}$ M ≡ ID99) on the component influx and efflux of $^{86}Rb^+$ were examined in L1210 cells incubated in Krebs-Ringer bicarbonate buffer (pH 7.4) for various times. The table shows the percentage decrease in equilibrium loading and initial rates of uptake and efflux of $^{86}Rb^+$ in treated cells compared to controls.

| *Time (h)* | Δ *Viability* | $^{86}Rb^+$ *loading* | $^{86}Rb^+$ *uptake* | $^{86}Rb^+$ *efflux* |
|---|---|---|---|---|
| 1 | 4 | 7 | 37 | 3 |
| 2 | 6 | 19 | 63 | 3 |
| 3 | 14 | 52 | 83 | 17 |
| 4 | 51 | 74 | 89 | 73 |

After 3 h of incubation, cell viability, as estimated by trypan blue exclusion, was >86% of control. The equilibrium loading of the cells with $^{86}Rb^+$ was reduced at this time by 52% as a result of an 83% inhibition of $^{86}Rb^+$ influx. The negligible effect on $^{86}Rb^+$ efflux at 3h increases to 73% inhibition at 4 h concomitant with a loss in cell viability. These results suggest that the inhibition of $^{86}Rb^+$ equilibrium loading is not due to generalised membrane damage, but rather to a specific lesion.

### Structural requirements for the cytotoxicity of CB 1954 (2,4 dinitro-5-aziridinyl benzamide) towards Walker cells

K.J. Hunter[1], J.M. Walling[1], I.J. Stratford[1] & D. Wilman[2]

[1]*MRC Radiobiology Unit, Harwell, Didcot, Oxon and* [2]*Institute of Cancer Research, Sutton, Surrey, UK.*

The Walker 256 mammary carcinoma has been found to be uniquely sensitive to the monofunctional alkylating agent CB 1954 (Cobb *et al.*, 1969, *Biochem. Pharmacol.*, **18**, 1519). We have investigated the *in vitro* sensitivity of a pair of cell lines derived from this tumour towards CB 1954 and a range of structural analogues. The parent cell line (WS) is sensitive to bifunctional alkylating agents, whereas the derived cell line (WR) is resistant. Both cell lines show the identical exquisite toxicity to CB 1954 but towards other monofunctional agents the two cell lines show similar sensitivities to other normal, cultured mammalian cell lines.

Progressive methyl substitution of the amide results in a concomitant loss of cytotoxicity towards the Walker cells, e.g. 5 $\mu$M CB 1954 for one hour reduced survival to $10^{-2}$ whereas 50 $\mu$M of the methyl amide was required to give the same degree of cell kill. (N.B. >2 mM is required for both compounds in V79 cells). Replacement of one of the nitro groups with an amine resulted in a complete loss of toxicity as did opening of the aziridine to give a dimethyl-amine. We have evidence to suggest that at least part of the cytotoxicity of CB 1954 is due to mitotic inhibition and NAD depletion.

**Fluoroacetate production by fluoroethylnitrosoureas**

R.A. Brennan & M.J. Tisdale

*CRC Experimental Chemotherapy Group, Department of Pharmacy, University of Aston, Birmingham B4 7ET, UK.*

The role of fluoroacetate in the toxicity and antitumour activity of the fluoroethylnitrosoureas, BFNU [N, $N^1$-bis (2-fluoroethyl)-N-nitrosourea] and FCNU [N-(2-fluoroethyl)-N-cyclohexyl-N-nitrosourea] has been studied in CBA mice bearing the TLX5 lymphoma either sensitive (TLXS) or resistant (TLX RT) to nitrosoureas. Any fluoroacetate formed might be expected to be converted into fluorocitrate, which has a blocking effect on aconitase. This leads to an accumulation of citrate in the affected tissue and cell death.

Treatment of mice bearing the TLXS tumour with either BFNU or FCNU caused an elevation in the citrate levels of heart, kidney and tumour, but not the liver 24 h after drug administration. Heart citrate levels were maximally elevated 10-fold, while the levels in kidney and tumour were increased 3 to 6-fold. A similar differential response of liver and kidney to fluoroacetate poisoning has previously been reported (Spencer & Lowenstein, 1967, *Biochem. J.*, **103,** 342), and may be a function of the different specificities of acetyl-CoA synthetase of liver and kidney. Deproteinised supernatants from heart, kidney and tumour inhibited purified aconitase.

Treatment of mice bearing the resistant tumour (TLX RT) with either BFNU or FCNU also caused an elevation of citrate levels in heart, kidney and tumour. Since these agents have no antitumour activity against this tumour it suggests that fluoroacetate formation is not related to therapeutic efficacy, but may be associated with generalised toxicity of the fluoroethylnitrosoureas.

**Potentiation of the effects of bleomycin and BCNU on A549 cell cultures by inhibitors of poly(ADP-ribose) polymerase**

D.A. Gray, J.M. Lunn & A.L. Harris

*Cancer Research Unit, University of Newcastle upon Tyne, UK.*

Poly(ADP-ribose) polymerase has been implicated in the process of DNA repair, specifically at the ligation step (Creissen & Shall, 1982, *Nature,* **296,** 271).

Exposure of A549 cells (derived from a human lung carcinoma) to Bleomycin (BLM,5–500 $\mu g\, ml^{-1}$) for 30 min caused extensive DNA strand breaking, as revealed by alkaline sucrose gradient centrifugation. Rejoining of the strands was inhibited by 3-acetamidobenzamide (3-AAB, 2.5 mM). BLM (50–500 $\mu g\, ml^{-1}$) also stimulated poly(ADP-ribose) polymerase activity in the cells, as measured by incorporation of $^3$H-NAD into TCA-insoluble material, and this stimulation was suppressed by 3-aminobenzamide (3-AB, 3 mM) or 3-AAB (2.5 mM). Cell survival at 5 days, following a 30 min exposure to BLM (50 $\mu g\, ml^{-1}$), was decreased 90%. The presence of 3-AAB (2.5 mM) in the growth medium potentiated this effect approximately two-fold.

BCNU (50 $\mu$M, 30 min) produced the same level of cytotoxicity as BLM (50 $\mu g\, ml^{-1}$, 30 min), but the effect of BCNU was potentiated much more markedly (5–10-fold) by 3-AAB (2.5 mM). However, it failed to produce either DNA strand breaks, detectable by alkaline sucrose gradient centrifugation, or a stimulation of poly(ADP-ribose) polymerase activity.

These results suggest that poly(ADP-ribose) polymerase inhibitors have other biochemical effects.

**Clonogenicity of human lung cancer cells**

P. Twentyman & G. Walls

*MRC Clinical Oncology and Radiotherapeutics Unit, Hills Road, Cambridge CB2 2QH, UK.*

We are interested in studying the biological and therapeutic heterogeneity of cells from human lung cancers and to do this we wish to isolate clonal subpopulations of cells either directly from clinical material or from very early *in vitro* cultures. We have, therefore, carried out experiments to determine the optimal *in vitro* cloning conditions for human lung cancer cells. A number of established human cancer cell lines have been used, growing either *in vitro* or as xenografts in nude mice. In addition we have used several early cultures established from marrow or lymph node biopsy specimens taken from small cell lung cancer patients and grown in defined HITES medium (Simms *et al.*, 1980, *Cancer Res.*, **40**, 4356) with or without 2.5% foetal calf serum, as well as specimens directly from the clinic. In a comparison of two soft agar cloning assays for a wide range of specimens we found a higher plating efficiency in the method of Courtenay & Mills, (*Br. J. Cancer.*, 1978, **37**, 261) than in the double-layer technique described by Carney *et al.*, 1980, (*Cancer Res.*, **40**, 1820). In addition the colonies were larger and easier to isolate using the former technique, probably due to the repeated medium replenishment involved. Varying the conditions of the Courtenay assay produces different effects for different cell types. For a small cell lung cancer line, the presence of rat red cells in the agar plug was a critical factor whereas it was less important for an adenocarcinoma cell line and a large cell anaplastic cell line. In general, growth in atmospheres containing 5% or less of oxygen was better than in 20% oxygen. Addition of irradiated feeder cells was of variable value. These factors are closely interrelated and do not operate as independent variables.

### Two human ovarian adenocarcinoma cell lines derived before and after clinical resistance to cytotoxic drugs

C.R. Wolf[1], S.S. Lawrie[1], D.J. Adams[1], A.R.R. Laudonio[1], I.P. Hayward[1], M.A. McIntyre[2], K.E. Buckton[3] & J.F. Smyth[1]

[1]*Imperial Cancer Research Fund, Medical Oncology Unit,* [2]*Dept. of Pathology and* [3]*MRC Clinical and Population Cytogenetics Unit, Western General Hospital, Edinburgh, UK.*

Two human ovarian cell lines, PEO1 and PEO4, have been established from ascitic fluid samples of a patient before and after the development of clinical resistance to a combination of 5-fluorouracil, chlorambucil and cis-platinum. The two cell lines exhibited certain similarities but also some significant differences. Both lines contained vesicular nuclei with prominent and occasional multiple nucleoli and vacuolated cytoplasm. "Signet ring" mucin containing cells were also present. A significant number of rearranged chromosomes were observed in cell lines PEO1 and PEO4. Abnormal chromosomes in common were 3, 9, 13 and 22. PEO4 had a chromosome 8 and 17 not identifiable in PEO1. Significant differences between PEO1 and PEO4 – in their doubling times (33 h *vs* 24 h), plating efficiency in plastic (0.8% *vs* 5.7%) and in agar (0.1% *vs* 4.0% early passage, 4.6% *vs* 14.0% later passage), contact inhibition and sensitivity to trypsin treatment were observed. In addition glutathione and glutathione S-transferase activities (components intimately involved with the deactivation of alkylating agents) were approximately 2–5 fold higher in PEO4 than PEO1. Mean glutathione concentrations were 0.8 and 5.9 nmol $10^6$ cells$^{-1}$ and transferase activities were 35 *vs* 159 nmol conjugate formed min$^{-1}10^6$cells$^{-1}$. Preliminary experiments indicate a much higher susceptibility of PEO1 to cis-platinum than PEO4. For drugs which require metabolic activation the clonogenic Courtenay assay has been modified to incorporate an activating system. In this system cyclophosphamide was highly active against PEO4.

### Phenotypic analysis of cell lines grown from Hodgkins Disease (HD) biopsy material

D.B. Jones[1], S. Scott[2], H. Stein[3] & D.H. Wright[1]

[1]*Departments of Pathology, Southampton and* [3]*Berlin and* [2]*Department of Haematology, Corkridge Hospital, Leeds, UK.*

The origin of the Reed-Sternberg cell (RS) and Mononuclear Hodgkins cell (Mhc) characteristic of HD remains a mystery. Previous studies have suggested an origin from T cells, B cells or macrophages. Cell lines grown in Southampton and Germany from HD Biopsy tissue have enabled us to revise opinions on the RS/MHc lineage. Two tetraploid cell lines (CO and L428 have been examined in detail. The cells do not synthesize immunoglobulin and are negative both for the Epstein Barr Nuclear antigen (EBNA) and EB transformed lymphoblastoid line antigens. When examined with a panel of moncolonal antibodies the cells do not express a phenotype characteristic of T cells, B cells or macrophages though staining (sometimes intracellular) is observed with some myeloid/macrophage antibodies and with a minority of anti-T cell antibodies. Histiocyte markers (Isaacson & Jones, 1983, *Histochem. J.*, **15**, 621) are absent. The unique phenotype is confirmed by the observation that a monoclonal antibody ki-1, raised to L428 cells which stains both cell lines and RS and MHC cells also identifies a unique population of cells present in reactive lymph nodes and tonsils. Isoelectric focussing shows that CO exhibits a unique esterase isoenzyme pattern with major bands of PI less than 5.4. The data suggest that RS and MHC are derived from a unique cell population. The ability of both lines to bind T cells may be relevant to the stromal reaction observed in HD.

### Human tumour cell lines established from xenografts in immunodeficient mice or rats. Characterisation by horse-radish peroxidase visualisation of Con-A binding sites on Western blots of 2-D PAGE

J. Walton, D. Winterbourne, A. Fiennes,
J. Hermon-Taylor & A. Grant

*Department of Surgery, St. George's Hospital Medical School, London SW17 0RE, UK.*

Four primary tumour explants obtained from patients with pancreatic (WAD), colonic (CAS, CAU; Davies *et al.*, 1981, *Br. J. Can.* **43,** 53), and renal cancer (GYL; Matthews *et al.*, 1981, *Urol. Res.*, **10,** 293) which initially grew only as xenografts in nude mice (nu/nu) or rats (rnu/rnu) have been established in tissue culture. Xenografts had to be passaged at least 4 times *in vivo* before *in vitro* growth was maintained. All of the cell lines had a human karyotype with no evidence of infiltrating mouse stroma. Each retained its tumourgenicity, could be regrown as a xenograft and showed a characteristic morphology in culture.

Glycoproteins on 2-dimensional polyacrylamide gel electrophoresis of Urea/NP-40 extracts from tumour cells were detected on Western blots with Con-A visualised with horseradish peroxidase. The majority of the Con-A binding glycoproteins were common to all cell lines but the intensity of labelling varied and each cell line exhibited characteristic marker glycoproteins.

### Astrocytoma xenografts in the choice of a second generation triazene

D.E.V. Wilman[1], N.J. Bradley[2] & S.G. Richardson[2]

[1]*CRC Laboratory, Institute of Cancer Research, Sutton, Surrey, SM2 5PX, and* [2]*Chester Beatty Laboratories, Institute of Cancer Research, Fulham Road, London, SW3 6JB, UK.*

We have reported several times in the past on our investigations related to the search for an aryltriazene as a suitable second-generation analogue of 5-(3,3-dimethyl-1-triazeno)imidazole-4-carboxamide (DTIC, Dacarbazine). Our original approach, which included a detailed structure-activity study (Connors *et al.*, 1976, *Biochem. Pharmacol.*, **25,** 241), was aimed at overcoming problems which reputedly arose from the photodecomposition of DTIC. As this is no longer regarded as a problem, though not losing sight of this objective, we have now become interested in the response of astrocytomas to these compounds. Grade IV astrocytoma xenografts grown as s.c. flank implants in immune deprived mice are sensitive to a number of clinical drugs, e.g. nitrosoureas, procarbazine, DTIC and a number of aryltriazenes, whereas the Grade III tumours show relatively little response. We have chosen one of these sensitive Grade IV astrocytomas (508) for a more detailed study. When implanted intracerebrally this tumour is sensitive only to nitrosoureas and aryltriazenes, compounds which meet Levin's criteria for compounds to cross the blood-brain barrier (*J. Med. Chem.*, 1980, **23,** 682). The flank implant of this tumour is sensitive to a range of aryldialkyltriazenes resulting in the "cure" of a number of implants. Testing of these compounds in the intracranial system will assist the choice of a suitable second-generation triazene to recommend for preclinical toxicology.

### The malignant transformation of mouse fibroblasts in the presence of human tumour xenografts

S. Sparrow[1], M. Jones[2], S. Billington[1] & B. Stace[1]

[1]*MRC Toxicology Unit, Woodmansterne Road, Carshalton, Surrey, &* [2]*Institute of Cancer Research, Clifton Avenue, Sutton, Surrey, UK.*

A human tumour xenograft programme designed to study chemotherapeutic agents has involved the transplantation of 75 different tumours into athymic mice over a period of 4 years. Some of these tumours have been passaged more than 20 times. During this period two tumours, an oat cell carcinoma of the lung and an ovarian carcinoma showed striking changes in their biology. There was a shortening of the passage interval from 259 days to 17 days and 274 days to 20 days respectively (tumours were passaged when they had reached a mass of 1–2 g), the karyotype changed from human to mouse, the LDH isoenzymes of the tumour developed mouse characteristics and the tumours became invasive with ulceration of the skin and metastases to regional lymph nodes and the lung, which is a feature rarely seen in xenografts. Histological examination of the tumours over three passages showed a gradual replacement of the human xenografts by proliferative fibroblastic tissue. Transformation of mouse fibroblasts when human tumour xenografts are propagated *in vitro* was demonstrated some time ago (Tveit *et al.*, 1980, *Br. J. Cancer,* **41,** 725) and it was recently shown that a similar phenomena could occur *in vivo* (Beattie *et al.*, 1982, *PNAS,* **79,** 3033).

**Preliminary results with new human tumour xenograft lines grown in immune-deprived mice**

J.A. Hanson, A. Pritchard & J.L. Moore

*Department of Radiobiology, Velindre Hospital, Cardiff, UK.*

CBA/Lac mice were immune-deprived by thymectomy and irradiation (Steel *et al.*, 1978, *Br. J. Cancer,* **37,** 224) and were kept for up to 6 weeks post-irradiation for subcutaneous tumour implantation. Thymectomised mice were housed in animal isolators. Xenografting attempts from 25 patient tumour samples, using either cell aggregates from fluid samples (7/25), tissue fragments from solid samples (11/25), or occasionally, cells from short-term culture (7/25) resulted in 7 tumour "takes" of which 4 have been passaged a number of times. Three of these new lines have been partially characterised. V2 is an adenocarcinoma of the colon. V7 and V24 are both from primitive cell type ovarian tumours. Quinacrine dihyrochloride fluorescence (Muller, 1976, *Stain Technol.,* **51,** 287), karyology or isoenzyme studies have confirmed the human origin of V2, V7 and V24. Sensitivity *in vitro* of V7 to vinblastine and cisplatin has been measured periodically over 16 months, using a soft agar assay (Courtenay & Mills, 1978, *Br. J. Cancer,* **37,** 261) and was found to be moderately stable, though the original patient sample obtained after chemotherapy and radiotherapy was markedly more resistant to vinblastine.

**The generation of monoclonal antibodies against human pancreatic exocrine cancer**

A. Grant, P. Harris, B. Pym[1] & J. Hermon-Taylor

*Department of Surgery, St. George's Hospital Medical School, London SW17 0RE, and [1]ICRF, Lincoln's Inn Fields, London, UK.*

Five different immunization methods for the stimulation of mouse spleen cells to produce antibodies selective for human pancreatic cancer cells (GER) have been investigated. Spleen cells from mice immunized with (i) viable GER cells, (ii) pancreatic tumour xenografts, (iii) serum from nude mice bearing pancreatic tumour xenografts, (iv) reconstituted chimeric animals and (v) spleens taken directly from nude mice bearing tumour xenografts, have been fused with mouse myeloma cells NSO/1. Supernatants from hybrid containing wells were tested for immunoglobulin (Ig) production by screening against a pannel of glutaraldehyde fixed human tumour cells and pooled normal human lymphocytes using sheep anti-mouse Ig urease-conjugated antibody in a modified ELISA assay.

Using all methods, 10–20% of the hybrid wells produced Ig which bound to pancreatic tumour cells but only 2–3% (160/849 wells) showed some selectivity for these cells. Ten of these wells have retained their selectivity on cloning. The antigens to which these antibodies (mainly $IgG_1$) bind are being characterised by immunofluorescence and immunoperoxidase staining of fresh and frozen tissue sections and 2-D PAGE. Mice immunized with tumour-bearer serum or reconstituted chimeric animals yielded fewer hybrid containing wells and fewer colonies per well, but proportionally more of these were selective for GER cells. Successful fusions were obtained from the spleens of nude mice bearing pancreatic tumour despite their incomplete T-cell system.